# Self-Assembling Cyclic Peptide Nanotubes for the Delivery of Doxorubicin into Drug-Resistant Cancer Cells

**DOI:** 10.1021/acsami.5c05264

**Published:** 2025-08-28

**Authors:** Marcos Vilela-Picos, Eva González-Freire, Federica Novelli, Yeray Folgar-Cameán, José Brea, Manuel Amorín, Juan R. Granja

**Affiliations:** † Centro Singular de Investigación en Química Biolóxica e Materiais Moleculares (CiQUS), Departamento de Química Orgánica, 16780 Universidade de Santiago de Compostela, 15782 Santiago de Compostela, Spain; ‡ Centro Singular de Investigación en Medicina Molecular e Enfermidades Crónicas (CiMUS), Departamento de Farmacología, Farmacia y Tecnología Farmacéutica. Facultad de Farmacia, 16780 Universidade de Santiago de Compostela, 15782 Santiago de Compostela, Spain

**Keywords:** cyclic peptides, self-assembly, peptide nanotubes, drug delivery, anticancer, cell resistance

## Abstract

Synthetic antimicrobial cyclic peptides conjugated to an antitumoral drug are used against drug-resistant cancer cells for a combined drug delivery strategy. The antimicrobial peptides are based on nanotube-forming cyclic peptides of alternating chirality, whose amphipathic and cationic characteristics determine their propensity to mainly interact with cell membranes rich in anionic phospholipids. This affinity triggers the formation of a supramolecular structure capable of destabilizing cell membranes such as those present in endosomes, thereby facilitating the delivery of the therapeutic agent to the cell nucleus and circumventing the cellular resistance mechanisms associated with efflux pumps.

## Graphical abstract

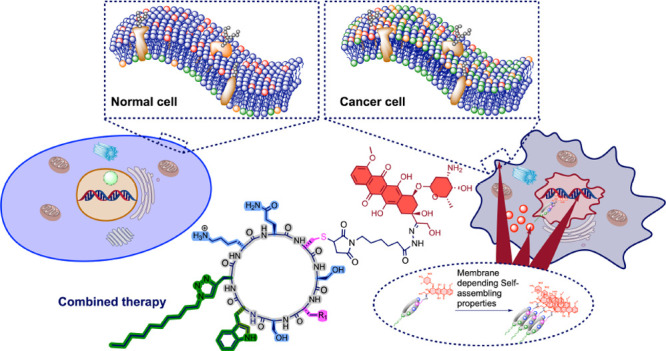

## Introduction

Cancer is a disease marked by the uncontrolled proliferation of cells. Despite significant advancements in recent years, it remains a leading cause of mortality worldwide.^1^ The efficacy and effectiveness of current therapeutic strategies are constrained by several factors.^2^ For instance, drug access to the entire tumor volume is often limited by the complexity and heterogeneity of the tumor. In addition, the development of resistance to chemotherapy may be associated with the tumor’s microenvironment.^3^ Cancer cell resistance is typically the result of the overexpression of membrane proteins that expel the drug from the cell, preventing the drug from exerting its therapeutic effect.^4^ This limitation, the resistance to cancer chemotherapy, is closely related to another important challenge to the health of advanced societies: the infectious diseases caused by multidrug-resistant superbugs.^5^ In recent years, immunotherapy, among others, has emerged as a highly promising alternative in the “fight” against neoplastic diseases by training the immune system of patients to seek, identify and target the abnormal cells within their own body to eliminate them, in a similar way to how it acts against invasive diseases.^6^ However, it is still necessary to delve deeper into the search for new strategies based on chemotherapies.^2^ In this sense, peptides have emerged as a promising alternative. In particular, host defense peptides (HDPs), also known as antimicrobial peptides, exhibit characteristics that have the potential to address the limitations of conventional therapeutic agents.^7^ The HDPs are essential natural compounds of the host’s innate immune system found in almost all living organisms. Furthermore, they are capable of modulating immune responses and reducing inflammatory responses to control infections.^8^ They are generally small cationic amphipathic peptides whose electrostatic interactions with highly anionic bacterial membranes are one of the driving forces of their antimicrobial mode of action (Figure 1).^9^ In recent years, several discoveries have suggested their potential anticancer activity.^10^ Although the membranes of cancer cells do not contain as many anionic components as those of bacteria, phosphatidylserine has been shown to accumulate on the outer leaflets of many cancer cells (Figure 1). Furthermore, these cells exhibit augmented negative potential within the cell^11,12^ and most of them contain altered *O*-glycosylated mucins.^13^ All these features give rise to an additional negative charge on the surface of the cancer cells, providing different properties to their cell membranes.^14^ Several HDPs are currently under investigation for this purpose, in which the drug must reach the tumor cell surface and disrupt the membrane function.^15^

**Figure 1:**
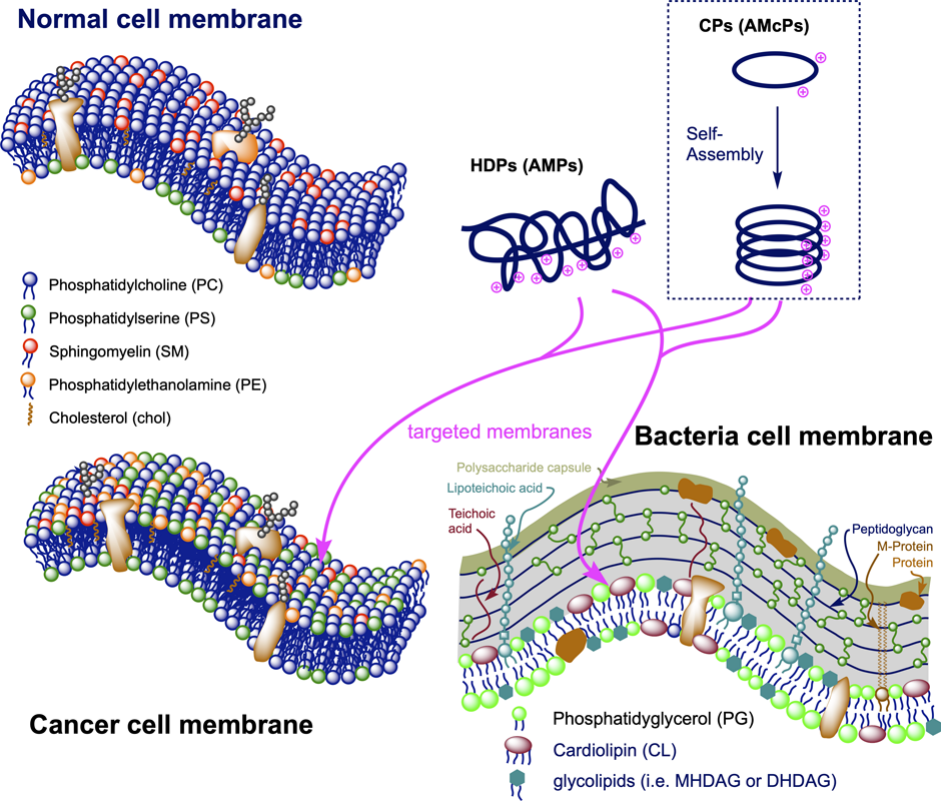
Schematic representation of some features of normal, tumoral, and bacteria membranes with respect to lipid composition and the presence of anionic phospholipids (e.g., PS, PG) as one of the driving forces for the selective interaction of antimicrobial peptides.

Targeted drugs and nanocarriers for delivering anticancer drugs into tumor cells also provide new tools to overcome the limitations of current treatments.^16–18^ In this context, single- or multistimulus responsive drug delivery systems have emerged as a promising alternative to enhance the activity of existing drugs.^19,20^ These systems are usually designed to prevent internalization in healthy cells, while ensuring the release of the entrapped drug inside cancer cells in response to intrinsic stimuli, such as variations in pH, redox conditions, or enzyme-mediated action. Alternatively, the use of certain external stimuli, such as the application of magnetic fields or light irradiation, has also been proposed.^19,20^

In this context, self-assembling cyclic peptide nanotubes (SCPNs) represent an attractive approach for the development of innovative delivery systems, due to their versatile properties.^21^ SCPNs are hollow supramolecular polymers formed by the stacking of disc-shaped cyclic peptides (CPs) through the formation of β-sheet-like hydrogen bonds.^22,23^ Specifically, membrane-interacting SCPNs are of particular interest as they can be used for different biomedical applications by tuning their orientation, aggregation state, or selectivity.^24–26^ In this regard, cyclic peptide-based antimicrobial agents represent an attractive alternative for the development of artificial derivatives that can substitute the natural HDPs.^27,28^ Recently, our group reported a new class of amphipathic SCPNs with antimicrobial activity using cyclic peptides bearing an alkyl chain.^29^ These cyclic peptides were generated by using a synthetic strategy based on two orthogonal click-type reactions, which allow the modification of the peptide scaffolds at the end of the synthesis by incorporating either hydrophilic or hydrophobic moieties. In particular, different hydrocarbon chains were attached by a *copper-catalyzed alkyne–azide cycloaddition* (CuAAC) to modulate the hydrophobic character and the antimicrobial activity. Furthermore, an *O*-alkyl oxime was used to attach saccharides in order to improve their biocompatibility by reducing their hemolytic side effects.^30^ The proposed mechanism of action involves the electrostatic interaction of cyclic peptides with the anionic surface of bacterial membranes, which facilitates the insertion of their alkyl moiety into the lipophilic part of the phospholipid bilayer, triggering its assembly into nanotubes.^31–33^ The resulting CPs showed remarkable activity and selectivity with a fast-killing mechanism. In this context, we envisage the development of a new kind of hybrid material by conjugation of a cytotoxic drug to SCPNs. This strategy could provide a combined therapeutic effect of the peptide and the drug.^34^ In this strategy, the cyclic peptide would not only act as a drug delivery system but would also have its own therapeutic activity based on its membrane-disrupting function. Examples of this include transmembrane nanotubes^35–37^ and cyclic peptide-polymer conjugates,^21,38–40^ but the challenge remains of extending this function to other types of SCPNs.

Here, we describe the conjugation of the anticancer drug doxorubicin to nanotube-forming cyclic peptides (Figure 2). Doxorubicin is a chemotherapeutic drug that has been in clinical use for many years. It is a member of the anthracycline group of drugs and has been employed in the treatment of different cancers.^41^ It is also widely used as an antitumoral agent in nanoscale delivery systems.^42–44^ However, there are several limitations of its use in clinical practice, including cardiotoxicity and resistance. In this work, we propose using a dynamic covalent bond, a hydrazone, for the incorporation of the drug into the cyclic peptide scaffold. The hydrazone is a dynamic covalent bond, exhibiting a significantly faster hydrolysis in acidic conditions when compared to oximes.^45,46^ We therefore speculate that the more acidic pH of tumor cells compared to healthy cells would facilitate the cleavage of this bond and the subsequent release of the drug within the cancer cell. To this end, we propose to utilize the nucleophilic properties of the thiol group of Cys to link the CP to a drug-substituted maleimide derivative. In addition, the incorporation of a propargylglycine residue should also allow the tuning of the hydrophobicity through the incorporation of an alkyl chain of appropriate length via the CuAAC reaction. For this study, we have used hydrocarbon tails of 6, 10, or 14 carbons (**CP1Tn** and **CP2Tn**).

**Figure 2:**
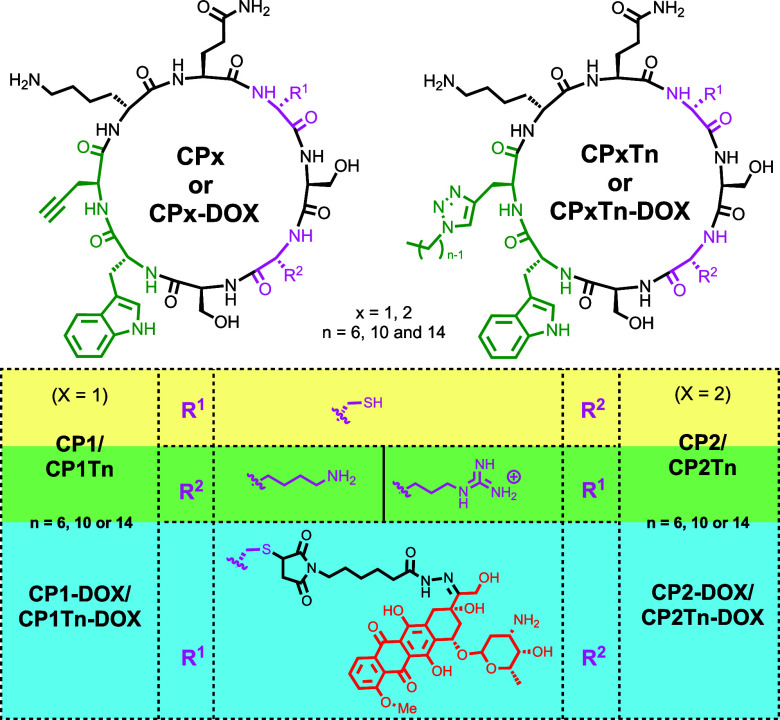
Structures of the cyclic peptides (**CPx** or **CPxTn**) and their corresponding conjugates with doxorubicin (**CPx-DOX** and **CPxTn-DOX**) studied in this work. Peptides **CP1** and **CP2** differ in the position where Cys is placed (magenta), while **Tn** indicates the different alkyl chains in which “*n”* provides information regarding the number of carbon atoms of this chain. In the table, the basic residues that are different in both types of CPs (*R* ^1^/*R* ^2^) are highlighted in green, the substitution and location of Cys is marked in yellow, while the side chain carrying DOX and its connector are highlighted in blue.

## Materials and Methods

### Materials

Chemical reagents were purchased from Sigma-Aldrich, Iris Biotech, TCI, Alfa Aesar, Fisher Scientific, Carbolution, and Sphere Fluidics. All solvents used were HPLC or synthesis grade. In the case of dry CH_2_Cl_2_, it was distilled over CaH_2_. NMR spectra were performed on a Bruker AVIII 500 MHz or a Varian 300 MHz spectrometer using the specified solvent, concentration, and temperature. Chemical shifts (δ) are reported in parts per million (ppm) relative to TMS (δ = 0) or the solvent signals. ^1^H NMR signals were assigned as singlet (s), doublet (d), triplet (t), multiplet (m), or broad (br). The coupling constants (*J*) are given in Hz. ^1^H NMR spectra of the peptides were assigned using 2D COSY and TOCSY. The water signal was suppressed when required. ^13^C NMR spectra were assigned using Distortionless Enhancement by Polarization Transfer 135 (DEPT-135). Peptide purification was performed on a semipreparative HPLC Hitachi D-7000 equipped with a Phenomenex Luna 5 μm-C18 column (100 Å, 250 × 10 mm) and using H_2_O (0.1% TFA)/MeCN (0.1% TFA) gradients as eluents. Ultrahigh-pressure liquid chromatography coupled with mass spectrometry (uHPLC–MS) analyses were carried out on an Agilent Technologies 1260 Infinity II with a 6120 Quadrupole LC–MS using an Agilent SB-C18 column. High Resolution Mass Spectrometry (HR–MS) was performed using ESI–MS in a Bruker MicroTof II mass spectrometer. Data are expressed in units of mass per unit of load (*m*/*z*).

### Synthesis

For detailed descriptions of the preparation methods of cyclic peptide derivatives and precursors (Figures S1, S14–S15 and S40), see the accompanying supporting materials. All CPs, after the corresponding solid phase synthesis, were purified by RP-HPLC and were obtained with purities higher than 98% (Figures S16–S49 with characterization data).

### ThT Fluorescence Assay

Thioflavin T (ThT)^47,48^ fluorescence experiments were performed in a Varian Cary Eclipse spectrophotometer equipped with a temperature-controlled cell chamber. Spectra were recorded at 20 °C in a Hellma fluorescence quartz cuvette (10 × 4 mm), using an excitation wavelength of 440 nm. Samples containing ThT (20 μM) and the corresponding peptide at the specified concentration ranges and buffers were prepared. The resulting solutions were allowed to equilibrate for 30 min before measuring fluorescence.

### Fluorescence Assay

Fluorescence emission experiments were carried out with a Varian Cary Eclipse spectrophotometer equipped with a temperature-controlled cell chamber and by using a Hellma fluorescence quartz cell (10 × 4 mm). Spectra were recorded from 500 to 800 nm at 20 °C by using an excitation wavelength of 480 nm and the specified pH and concentration. The solutions were allowed to equilibrate for 30 min before measurements were performed.

### Circular Dichroism

Circular dichroism (CD) measurements were acquired in a Jasco J-1100 CD Spectrometer equipped with a Jasco MCB-100 mini Circulation Bath for controlling the temperature. Measurements were carried out in a 0.5 cm quartz cuvette at 20 °C and the specified pH and concentration. The equipment was configured for 100 nm.min^–1^ scanning speed, 1 s response time, 1 nm bandwidth, and 0.2 nm data pitch. Each spectrum corresponds to the average of 10 scans, and the solvent background was corrected.

### Scanning Transmission Electron Microscopy and Transmission Electron Microscopy (STEM and TEM)

STEM images were obtained using a ZEISS FESEM ULTRA Plus with EDX operating at an extra-high tension of 20 kV. TEM images were acquired with JEOL JEM-2010 equipment. Samples were prepared by the deposition of a peptide solution (200 μM, 10 μL) in PBS (10 mM, 107 mM NaCl, pH 7.4) over a 400-mesh carbon-coated copper grid (Electron Microscopy Sciences). After 10 min, an excess of the sample was removed with a filter paper. Then, samples were stained with an aqueous solution of phosphotungstic acid (2% w/v, 10 μL) for 3 min and washed with Milli-Q H_2_O (10 μL) for 1 min. Samples were allowed to air-dry overnight before imaging.

### Atomic Force Microscopy (AFM)

Atomic force microscopy measurements were performed at room temperature and ambient atmosphere using a Park Systems XE-100 in non-contact mode. ACTA tips were used (silicon tips; nominal values: spring constant = 40 N/m, frequency = 300 kHz, ROC less than 10 nm). Briefly, 10 μL of CP aqueous solutions at the specified pH and concentration were dropped on a silicon wafer or mica substrate. After 5 min, the sample was thoroughly washed with Milli-Q-H_2_O and dried under argon flow. Image analysis was carried out with Gwyddion.^49^

### Cell Viability Assays

The cell growth inhibition of each compound was evaluated using the MTT (3-[4,5-dimethylthiazol-2-yl]-2,5-diphenyltetrazolium bromide) assay.^50^ MCF-7 and MRC-5 cells were grown on a Minimum Essential Medium Eagle, while NCI-H460 and NCI/ADR-RES cells were grown on RPMI 1640 culture medium. All culture mediums were supplemented with 10% FBS (fetal bovine serum) and 1% penicillin-streptomycin-glutamine mix. The incubation conditions were an atmosphere of 95% air and 5% CO_2_ at 37 °C. MCF-7 (100,000 cells/mL), NCI-H460 (150,000 cells/mL), NCI/ADR-RES (150,000 cells/mL), or MRC-5 (100,000 cells/mL) cells were seeded the day before in sterile 96-well plates (100 μL/well). Then, cells were incubated with each concentration of the compound (100 μL/well) for the specified time for each cell line. The compounds were previously dissolved in DMSO and diluted at the time of the experiment with complete medium to the concentration to be tested. The DMSO content of each well was maintained at less than 1%. All concentrations were carried out with triplicate points, and controls with DMSO at the same proportion in which the compounds were dissolved were included in all experiments. After the incubation time, the samples were removed, and 100 μL of complete medium with 0.5 mg/mL MTT was added to each well. Cells were incubated for 4 h, and the medium was removed. The resulting formazan crystals were dissolved in DMSO (100 μL/well). The absorbance of each well at 595 nm was acquired with a Tecan Infinite F200Pro plate reader. A blank subtraction was performed (cells treated with Triton X-100), and the data were normalized to the value of the untreated cells (100% viability). Data were analyzed with GraphPad Prism software.^51^

### Confocal Microscopy Experiments

NCI/ADR-RES cells were seeded at 150,000 cells/mL in a 96-well plate (100 μL/well) the day before. The medium was RPMI 1640 with 10% FBS and a 1% penicillin-streptomycin-glutamine mix. Then, medium from cells was eliminated, and peptide solutions at the desired concentration in complete medium were added (100 μL/well). Cells were incubated with the compound during the specified time in an atmosphere of 95% air and 5% CO_2_ at 37 °C. For nuclear staining, the cell medium was removed after incubation with the cyclic peptide, and 50 μL of a Hoechst 33342 solution (1 μM) in cell medium was added per well. Cells were incubated for 30 min, and the medium was subsequently replaced with a complete medium solution (100 μL/well). For Lysotracker Deep Red staining, the cell medium was removed after incubation with the cyclic peptide, and 50 μL of a Lysotracker Deep Red solution (50 nM) in cell medium was added to each well. The cells were incubated for 30 min, and then the medium was replaced with 100 μL/well of complete medium. Cells were analyzed with an Andor DragonFly spinning disc confocal setup mounted on a Nikon Eclipse Ti-E inverted microscope. Images were analyzed using ImageJ.^52^

### Endocytosis Inhibition Experiments

NCI/ADR-RES cells were seeded the day before on a 96-well plate (150000 cells/mL). The next day, cells were treated with the corresponding solutions (100 μL/well) of Wortmannin (100 μM), Dynasore (80 μM), EIPA (50 μM), or chlorpromazine (30 μM), all of them diluted in RPMI 1640 medium with 10% FBS and a 1% penicillin-streptomycin-glutamine mix. These inhibitors were incubated for 30 min at 37 °C in an air atmosphere containing CO_2_ (5%). Then, these solutions were replaced by the samples containing the studied CPs (100 μL/well), **CP1-DOX**, **CP1T10-DOX**, **CP1T**_**NBD**_ **-DOX**, **CP2-DOX**, or **CP2T10-DOX** (50 μM), and the same amount of the corresponding inhibitor in RPMI 1640 without serum. After incubation for 1 h, cells were washed twice with PBS and detached with Trypsin (100 μL/well, incubated for 10 min). Once the cells were in suspension, Trypsin was neutralized with PBS containing 2% FBS and 5 mM EDTA (100 μL/well). The cell uptake was quantified with a Guava easyCyte BG HT flow cytometer. DOX was excited at 488 nm (blue laser), and the emission was collected at 580 nm (yellow channel). The median fluorescence intensity (MFI) was calculated for each sample. Each condition was measured in triplicate, and data were normalized to untreated controls. Data were analyzed with the InCyte analysis mode included in GuavaSoft 3.2 software.

### Cell Uptake Experiments

NCI/ADR-RES cells (10^6^) were seeded in T-25 Flasks. After 24 h, the medium was removed, and a solution of **DOX** or **CP1T**_**NBD**_ **-DOX** (100 μM) in culture medium (7 mL) was added. The cells were incubated for 120 min at 37 °C under a CO_2_ atmosphere (5%). Then, the medium was removed, and the cells were washed with cold PBS (3 × 3 mL). After washing, a mixture of ACN/H_2_O (1.5 mL, 80:20) was added to the flask and incubated overnight at 4 °C. Solvent samples were transferred to 1.5 mL Eppendorf and centrifuged at 12,000 *g* for 15 min at 4 °C, and the supernatant was evaporated to dryness. Analogous experiments were carried out by incubating the compounds at 4 °C for 120 min to determine the nonspecific binding to the cell membrane, since the uptake is decreased at this temperature. To evaluate the efficiency of solvent extraction, 10^6^ cells were seeded in a T-25 Flask. Twenty-four hours later, the medium was replaced by culture medium (7 mL) and incubated for 120 min at 37 °C under a CO_2_ atmosphere (5%). Then, the medium was removed, the cells were washed with cold PBS (3 × 3 mL), and finally, a solution of the corresponding compound (10 μM) in a mixture of ACN/H_2_O (15 mL, 80:20) was added. The cells were incubated overnight at 4 °C. Solvent samples were transferred to 1.5 mL Eppendorf and centrifuged at 12000 g for 15 min at 4 °C. The supernatant was evaporated to dryness. Experiments in the absence of cells were performed to determine the nonspecific binding to cell culture plates. The resulting lysates were analyzed by RP-HPLC by dissolving the Eppendorf samples in an aqueous solution of TFA (1%, 100 μL). All of the intracellular compound quantities were calculated from the detected area and divided by the efficiency of solvent extraction. Cell uptake was calculated by subtracting the intracellular compound quantity from the assay carried out at 37 °C from the quantity obtained at 4 °C and normalized to 100% vs the initial concentration incubated.

## Results and Discussion

### Synthesis

The synthesis of the different doxorubicin-cyclic peptide conjugates was carried out following the scheme illustrated in Figure 3. In this regard, we developed a synthetic strategy in which the final step is a Michael-type addition between a maleimide-based doxorubicin linker (**1**) and the thiol group of the corresponding cyclic peptide. The synthesis of **1** was accomplished in three steps following a previously described protocol (Figure S1).^53–55^ On the other hand, the synthesis of the cyclic peptide scaffolds was carried out using a Fmoc/^t^Bu solid-phase peptide synthesis on a Rink amide resin (Figure 3A).^29^ To facilitate subsequent cyclization on the solid support, the synthesis was initiated by attaching Fmoc-Glu-OAll through its side chain. This was followed by seven cycles of *N*-terminal (Fmoc) deprotection with piperidine/DMF (1:3) and coupling of the corresponding amino acid using *N*-HBTU as coupling reagent.^56^ Once the synthesis of the linear peptide was completed, the cyclization on the solid support was carried out using PyAOP as the coupling reagent. For designs without an alkyl chain, the resin was cleaved by treatment with a cocktail of TFA/DCM/H_2_O/EDT (90:5:2.5:2.5) to provide either **CP1** or **CP2**. For the other designs, the corresponding cyclic peptides were subjected to a solid-supported CuAAC reaction to incorporate the corresponding alkyl tail.^29^ Finally, the side chain deprotection and release of the CPs from the resin were performed by treatment with a cocktail of TFA/DCM/H_2_O/EDT (90:5:2.5:2.5). The different peptide scaffolds were purified by reversed-phase HPLC. Finally, the maleimide linker containing doxorubicin (**1**) was conjugated to the corresponding CP in DMF (Figure 3B).^53,54,57,58^ The resulting conjugates (**CP1-DOX**, **CP2-DOX**, **CP1T6-DOX**, **CP1T10-DOX**, **CP1T14-DOX**, **CP2T6-DOX**, **CP2T10-DOX,** and **CP2T14-DOX**) were finally purified by Sephadex LH-20.

**Figure 3:**
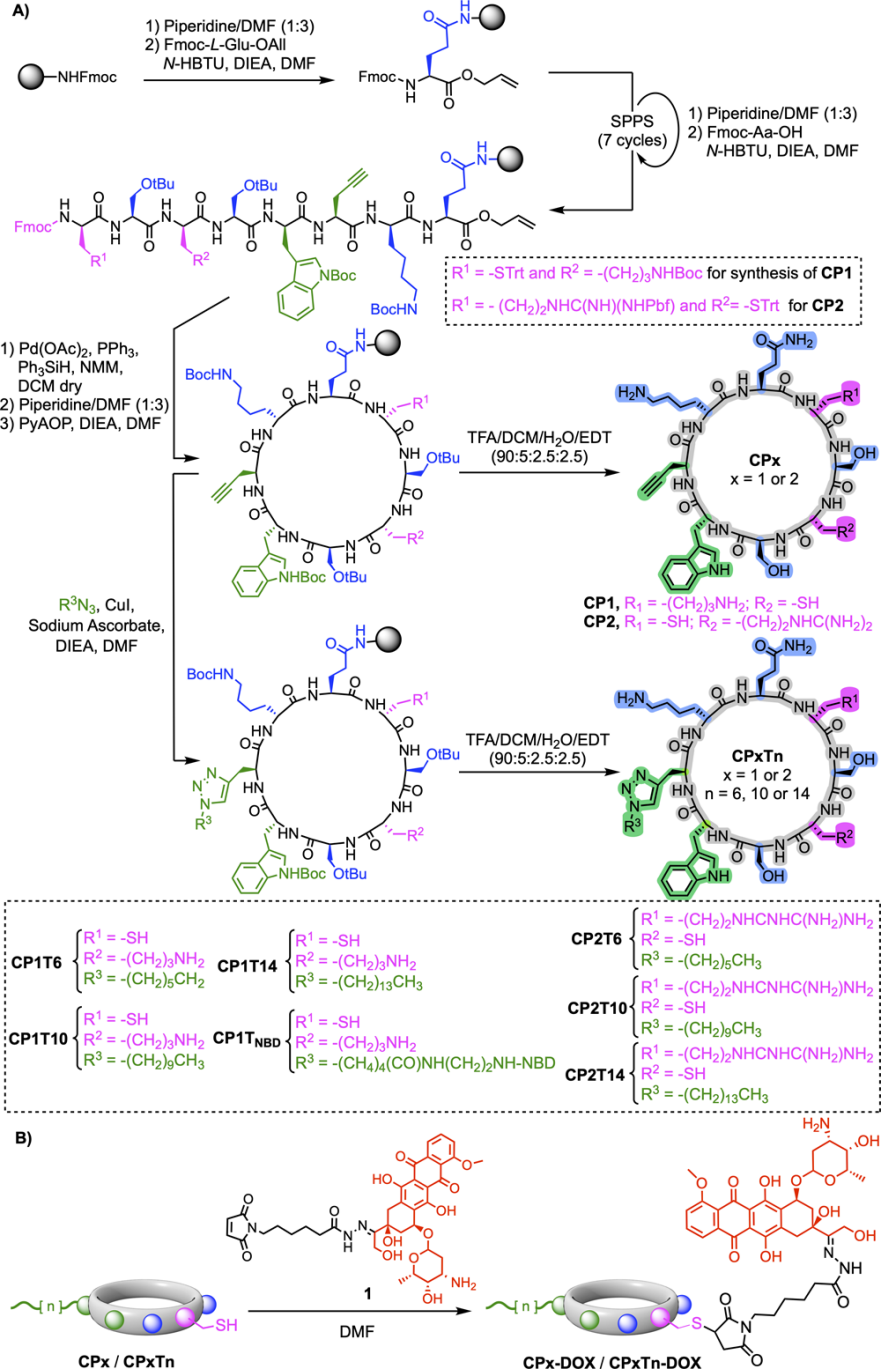
(A) Solid-phase synthesis on a Rink Amide resin of the cyclic peptide scaffolds of this work (**CP1**, **CP1Tn**, **CP2**, and **CP2Tn**). (B) Reaction of the doxorubicin linker (**1**) and the cyclic peptides scaffolds to provide the corresponding conjugates (**CP1-DOX**, **CP1Tn-DOX**, **CP2-DOX**, **CP2Tn-DOX**).

### Self-Assembly Experiments

Once the different peptides were synthesized, we proceeded with an evaluation of their self-assembly properties. Initially, we focused on studying the influence of the alkyl tail on the peptides without the conjugated drug (**CP1**, **CP2**, **CP1T10**, **CP2T10**). For this purpose, fluorescence experiments were performed in the presence of Thioflavin T (ThT), which is a dye that emits fluorescence upon interaction with β-sheet structures (Figure S2A–D).^47,48^ In all the cases, the spectra revealed the growth of ThT emission with increasing peptide concentrations, thus confirming the ability of the peptide scaffolds to form β-sheet structures. As expected, the critical aggregation concentration (*cac*) was found to be lower for the derivatives incorporating the decyl chain (8–10 μM for **CP2T10**, **CP1T10** compared with 38–41 μM for those lacking an alkyl chain, respectively, Figure S2 and Table S1), confirming that hydrophobic effects contribute to the stabilization of SCPNs. The morphology of the CP assemblies was evaluated by scanning transmission electron microscopy (STEM) (Figure S2E–H) by drop casting aqueous solutions of these CPs (200 μM) at pH 7.0 on copper grids. The images of **CP1** and **CP2** showed fibrils with lengths of several micrometres. In contrast, large 2D structures were obtained for the peptides containing the decyl moiety (**CP1T10** and **CP2T10**).^59,60^ These structures are likely formed by a hierarchical self-assembly process of cyclic peptides in a similar way as reported previously for other amphipathic cyclic peptides.^59,60^ Initially, they must form peptide nanotubes in which the alkyl chains are aligned along the nanotube, forming a hydrophobic area on the nanotube surface that would promote their subsequent aggregation, driven by hydrophobic effects, to form 2D sheets. These structures, as in previous studies with other amphipathic CPs,^59,60^ must be formed by bilayers of cyclic peptide nanotubes. The lateral growth of the SCPNs to form 2D structures is likely promoted to avoid contact of the aforementioned hydrophobic surface with the aqueous medium. Therefore, the resulting sheets have a central hydrophobic core consisting of alkyl chains and indole moieties of Trp. Cyclic peptides and their polar side chains are exposed on both sides of the bilayer and oriented toward the external medium.

Next, the impact of doxorubicin conjugation on the cyclic peptide assembly and nanotube formation was also investigated. This was studied for doxorubicin-CP conjugates (**CPx-DOX** or **CPxTn-DOX**) using fluorescence and circular dichroism (CD) to obtain the corresponding *cac*. First, doxorubicin fluorescence was measured at neutral pH as a function of the peptide concentration (Figures 4A and S3A–E). In all cases, the corresponding increase in fluorescence was observed up to a critical concentration, after which the emission started to decrease. This change was attributed to peptide self-assembly and aggregation. Concentration-dependent circular dichroism experiments at neutral pH showed an increase in negative CD signals for doxorubicin at 290 nm and 500–550 nm, together with a positive CD signal around 440 nm. These bands were not found in the spectrum of DOX (Figures 4B and S3F–J). The concentration dependence of these spectra suggests that the assembly of cyclic peptides must be associated with a DOX-DOX helical organization around the cyclic peptide nanotube. As expected, both fluorescence and CD experiments yielded a lower *cac* for the derivatives with the alkyl moiety than for those lacking it. Overall, the incorporation of the DOX moiety reduces the self-assembly properties of the resulting derivatives, as evidenced by the observed increase in *cac* (Figure S3 and Table S1). In addition, FT-IR spectra of the peptides as a powder revealed in the amide I region a band around 1629–1625 cm^–1^ and a shoulder around 1672–1668 cm^–1^ (Table S2, see also spectra in the characterization sections (Figures S20, S22, S24, S26, S28, S33, S35, S37, S39, S41–S49) corresponding to each derivative). These bands, together with the lack of high-frequency components above 1680 cm^–1^, suggest the formation of parallel β-sheet structures.^61^ In all cases, a strong amide A band between 3270 and 3276 cm^–1^ confirms the formation of strong hydrogen bonding interactions.^62^

**Figure 4:**
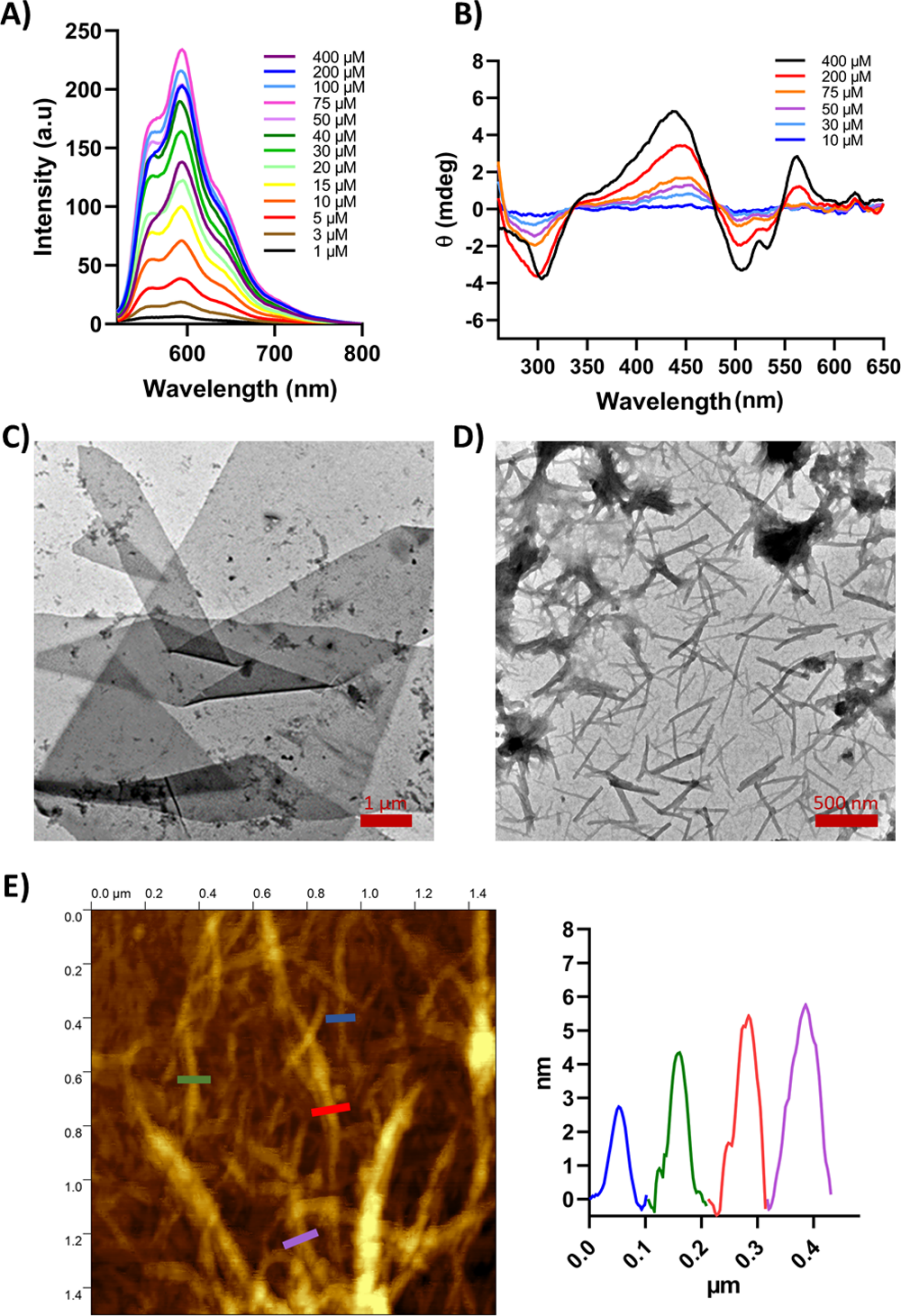
(A) Fluorescence spectra of **CP1T10-DOX** solutions in PBS (10 mM, 107 mM NaCl, pH 7.4) at different peptide concentrations (1–400 μM, λ_ex_ = 480 nm). (B) CD spectra of **CP1T10-DOX** in PBS (10 mM, 107 mM NaCl, pH 7.4) at different concentrations (10–400 μM). (C) TEM micrograph of a sample of **CP1-DOX** (200 μM) in PBS (10 mM, 107 mM NaCl, pH 7.4) deposited on carbon-coated Cu grids. Scale bar: 1 μm. (D) TEM micrograph of a sample of **CP1T10-DOX** (200 μM) in PBS (10 mM, 107 mM NaCl) at pH 7.4, deposited on carbon coated Cu grids. Scale bar: 500 nm. (E) AFM of **CP2T10-DOX** (200 μM) in PBS (10 mM, 107 mM NaCl, pH 7.4) deposited on mica. The height profiles were obtained along the color lines shown in the image.

STEM and TEM were also used to assess whether the doxorubicin-conjugated cyclic peptides still formed fibrillar and/or sheet-like structures analogous to their drug-free counterparts (Figures 4C,D and S4). For this purpose, a solution of **CP1-DOX** or **CP2-DOX** (200 μM) in phosphate buffer (10 mM PBS, pH 7.4) was drop-cast onto the carbon-coated Cu grids, and the resulting micrographs revealed the formation of large 2D structures with dimensions of several square μm, together with other smaller ones most probably resulting from the cracking of these sheets as a consequence of their manipulation. On the other hand, the grids of the analogues with the decyl pendants (**CP1T10-DOX** or **CP2T10-DOX**) showed nanotubes of several hundred nanometers in length. This result confirms that amphipathic cyclic peptides tend to form 2D structures, as previously discussed, through a hierarchical process. A nanotube bilayer is probably formed with the alkyl chains arranged in the inner part of the bilayer, driven by the hydrophobic effect. In order to stop the hierarchical processes that lead to the formation of these 2D laminas and obtain the precursor nanotubes, different depositions were made both in neutral and basic conditions (pH 9.0), even in the presence of increasing amounts of acetonitrile (up to 20%) in order to reduce the hydrophobic effects that drive the formation of the 2D structures (Figure S4C–D). However, in any of these conditions, the nanotubes were obtained, observing again the presence of sheets, which confirms the high propensity of these hybrids to form this type of 2D structure. The nanotube and sheet dimensions were further assessed by atomic force microscopy (AFM) after deposition of a solution of some of the CP conjugates (200 μM) on a mica surface (Figures 4E and S5). The images obtained for **CP2T10-DOX** revealed the presence of polydisperse fibers with a diameter ranging from 2.7 to 5.7 nm (Figure 4E), confirming the formation of different types of nanotube bundles. On the other hand, AFM images of **CP2-DOX** confirmed the presence of long sheets with an average height of 7.2 ± 0.9 nm (Figure S5), which correlates quite well with the proposed bilayer of nanotubes. It is worth noting that, in this case, the DOX conjugates possessing the alkyl chain form fibers instead of the sheets formed by the precursors (**CPxTn**) without DOX. These results emphasize the observed ability of this type of amphipathic cyclic peptides to form these 2D structures. In this case, the incorporation of DOX on the side opposite to the hydrophobic residues distorts the amphipathic balance of the CPs and thus their tendency to form the aforementioned nanotube bilayers.

### *In Vitro* Activity

The cytotoxic activity of these peptides was evaluated in three different cancer cell lines: a breast cancer cell line (MCF-7),^63,64^ a lung cancer cell line (NCI-H460),^65^ and a doxorubicin-resistant ovarian cancer cell line (NCI/ADR-RES).^66^ Also, the maleimide linker with doxorubicin (**1**), free doxorubicin, and cisplatin were used as controls (Table 1). First, the peptides were examined in the two nonresistant cell lines, breast (MCF-7) and lung (NCI-H460). The drug-free peptide scaffolds showed only moderate or no activity, except for **CP1,** which exhibited a maximum inhibition of more than 90% in both cell lines. In contrast, the cyclic peptide-doxorubicin conjugates had high activity, as evidenced by their high maximum cell growth inhibition (75–93%) and IC_50_ values in the range of 1–4 μM for all compounds. These values are similar or better than those obtained for the control cisplatin or compound **1**, as well as comparable to those obtained for free doxorubicin. No major differences were observed between the derivatives with and without the 10-carbon alkyl tail.

**Table 1:** *In Vitro* Peptides Activity in MCF-7 (Breast Cancer Cell Line) after Incubation for 96 h, NCI-H460 (Lung Cancer Cell Line) after 48 h, and NCI/ADR-RES (Doxorubicin-Resistant Ovarian Cancer Cell Line) after 48 h.^a^

	MCF-7 (96 h)		NCI-H460 (48 h)		NCI/ADR-RES (48 h)	
compound	*E*_max_ (% inhibition)	IC_50_ (μM)	*E*_max_ (% inhibition)	IC_50_ (μM)	*E*_max_ (% inhibition)	IC_50_ (μM)
**CP1**	92 ± 1	17.7 ± 0.3	94 ± 2	37.9 ± 0.4	90 ± 1	55.8 ± 0.7
**CP1-DOX**	81 ± 1	3.5 ± 0.3	75 ± 1	2.8 ± 0.1	2 ± 2	
**CP1T10**	59 ± 1	78.8 ± 0.1	28 ± 1		70 ± 2	68.9 ± 2.1
**CP1T10-DOX**	89 ± 1	3.4 ± 0.4	93 ± 1	3.1 ± 0.1	87 ± 2	38.4 ± 0.7
**CP2**	48 ± 1		8 ± 1		7 ± 3	
**CP2-DOX**	80 ± 1	1.8 ± 0.2	92 ± 1	2.9 ± 0.3	16 ± 3	
**CP2T10**	63 ± 1	50.2 ± 0.6	80 ± 2	>100	75 ± 2	68.2 ± 4.6
**CP2T10-DOX**	88 ± 1	2.6 ± 0.3	93 ± 1	1.7 ± 0.1	86 ± 1	33.4 ± 0.3
**Linker-DOX (1)**	80 ± 1	2.5 ± 0.6	58 ± 1	13.7 ± 0.1	35 ± 3	
**DOX**	86 ± 1	0.82 ± 0.1	56 ± 1	9.3 ± 1.7	36 ± 4	
**Cisplatin**	78 ± 1	7.8 ± 0.6	53 ± 1	5.1 ± 0.6	90 ± 1	11.5 ± 0.6

a *E*_max_ is the maximum effect, which corresponds to the inhibition obtained at the highest concentration tested (100 μM). IC_50_ is the concentration at which 50% growth inhibition is obtained, and it was calculated for only those compounds with an *E*_max_ value higher than 50%.

Considering these findings, we decided to study the activity of these hybrids in a doxorubicin-resistant ovarian cancer cell line (NCI/ADR-RES) to clarify whether the conjugation of DOX to cyclic peptides could overcome the resistance of these cells to this drug. Remarkably, these resistant cells overexpress the P-glycoprotein (P-gp), a transporter capable of expelling doxorubicin from the cell cytoplasm, thereby preventing its therapeutic action.^66,67^ CPs by themselves showed only moderate activity after 48 h, except for **CP2,** which presented a very low cytotoxicity. These compounds exhibited a maximum inhibition of approximately 75–95%, although their IC_50_ values were within the range of 55–69 μM. The alkyl chain appears to play a pivotal role, as evidenced by the absence of activity observed in the doxorubicin-conjugated derivatives without the hydrocarbon pendant (**CP1-DOX** and **CP2-DOX**). Conversely, the designs incorporating the 10-carbon alkyl chain (**CP1T10-DOX** and **CP2T10-DOX**) showed maximum cell growth inhibitions exceeding 85%. IC_50_ values were between 33 and 38 μM, which, while lower than the activity found with cisplatin, is a promising result given the resistance of these cells to doxorubicin, whose own activity in this cell line at 100 μM is only 36%. In addition, compound **1** demonstrated no activity. These results with cyclic peptide/DOX conjugates confirm the importance of the cyclic peptide and the hydrocarbon chain in overcoming cellular resistance to the drug, suggesting an alteration in the mechanism of drug entry and release. Once again, the relatively high cytotoxicity of **CP1** is surprising, with an IC_50_ of 55.8 μM, confirming that its mode of action is not related to that of DOX. Therefore, as expected, the presence of the transporters responsible for drug efflux (P-gp) in the NCI/ADR-RES cell line does not dramatically alter its activity.

Considering the potential significance of the hydrocarbon tail in overcoming cellular resistance, we decided to explore how the length of the alkyl moieties affects the drug activity (Table 2).^29^ Consequently, cyclic peptide derivatives with shorter (6 carbons) and longer (14 carbons) alkyl tails were prepared and evaluated in NCI/ADR-RES cells. In neither case did these new derivatives show a higher maximum cell growth inhibition than the homologues with the ten-carbon alkyl chain. Furthermore, the influence of incubation time on the activities of these compounds was also addressed in an attempt to determine the time dependence of the cytotoxic effect of the conjugates.^68^ In all cases, an increase in the potency was observed when the incubation time was extended from 48 to 72 h. As previously mentioned, after 48 h of incubation, only **CP1T10-DOX** and **CP2T10-DOX** demonstrated effective cell growth inhibition. However, good activity was also observed for **CP1T14-DOX**, **CP1T**_**NBD**_ **-DOX**, and **CP2T**_**14**_ **-DOX** at 72 h. These results demonstrate that conjugation with CPs not only sustains the effect over time but also enhances it. This can be attributed to the fact that DOX cannot be expelled by efflux pumps, and therefore its effect is seen with a single administration up to 72 h later.

**Table 2:** Effect of the Tail Length on the *In Vitro* Peptide Activity in NCI/ADR-RES (Doxorubicin-Resistant Ovarian Cancer Cell Line) after Incubation for 48 or 72 h.^a^

	NCl/ADR-RES (48 h)		NCl/ADR-RES (72 h)	
compound	*E*_max_ (% inhibition)	IC_50_ (μM)	*E*_max_ (% inhibition)	IC_50_ (μM)
**CP1-DOX**	2 ± 2		22 ± 4	
**CP1T6-DOX**	26 ± 2		22 ± 3	
**CP1T10-DOX**	87 ± 2	38.4 ± 0.7	84 ± 3	10.8 ± 1.7
**CP1T14-DOX**	44 ± 4		63 ± 2	5.7 ± 2.7
**CP1T**_**NBD**_ **-DOX**	30 ± 3		52 ± 2	3.7 ± 0.2
**CP2-DOX**	16 ± 3		13 ± 1	
**CP2T6-DOX**	22 ± 2		23 ± 0	
**CP2T10-DOX**	86 ± 1	33.4 ± 0.3	64 ± 7	21.8 ± 6.4
**CP2T14-DOX**	39 ± 9		51 ± 4	8.2 ± 0.8

a *E*_max_ is the maximum effect, which corresponds to the inhibition obtained at the highest concentration tested (100 μM). IC_50_ is the concentration at which 50% growth inhibition is obtained, and it was calculated only for those compounds with an *E*_max_ value higher than 50%.

In addition, **CP1T**_**NBD**_ **-DOX**, which has an NBD moiety attached to the triazole pendant and was prepared for subsequent study of the mechanism of action, *vide infra*, showed the lowest IC_50_ of all the peptides tested after 72 h (3.7 μM). Also, **CP1T14-DOX** showed an IC_50_ of 5.7 μM, while that of **CP2T14-DOX** was 8.2 μM. The derivatives without a tail or with a 6-carbon tail were poorly active, even after 72 h of incubation (*E*_max_ ∼ 25% at 100 μM), confirming the importance of incorporating an alkyl chain with an adequate length to facilitate the interaction with membranes.

The cytotoxicity of the peptides was also evaluated in a noncancerous cell line (fibroblasts). Specifically, the different peptides were incubated in a human embryonic lung cell line (MRC-5) for a period of 7 days (Table S3). As expected, doxorubicin demonstrated a significant cytotoxicity against these healthy cells. However, the binding of doxorubicin to the cyclic peptides is able to increase the IC_50_ in all the cases tested. This result suggests that the binding of doxorubicin to a cyclic peptide can enhance the potency of the drug in cancer cells while reducing its impact on healthy cells.

### Mechanism of Action Studies

We evaluated the cell internalization mechanism of the peptides in the NCI/ADR-RES cancer cells using confocal microscopy to understand the ability of the derivatives with the appropriate tail to overcome resistance. For this purpose, the cells were incubated with free **DOX**, **CP1-DOX**, **CP1T10-DOX**, **CP2-DOX**, and **CP2T10-DOX** at 50 μM for 2 h (Figure 5A). Images of **CP1-DOX** showed its internalization, although it appeared to be mainly trapped in small compartments of the cytoplasm, probably lysosomes or endosomes, as denoted by the dotted pattern observed inside the cell. An extended incubation time or higher peptide concentration was ineffective in overcoming this vesicular entrapment (Figure S6). Alternatively, **CP2-DOX** showed a minimum cell internalization after 2 h of the incubation period. Increasing the concentration or incubation time slightly improved its internalization, although again, it remained entrapped in cytoplasm vesicles (Figure S7). These observations suggest that the derivatives that lack the alkyl tail cannot promote endosomal escape, a finding that is consistent with their low toxicity in this cell line. On the other hand, images obtained with the CP derivatives containing the 10-carbon pendant (**CP1T10-DOX** and **CP2T10-DOX**) showed cells with a colored nucleus, thereby confirming the ability of the drug to reach the nucleus after 2 h and exert its therapeutic action after the same period (Figure 5A). Fluorescence with these derivatives was predominantly accumulated at the nuclear membrane. Finally, controls performed on NCI/ADR-RES cancer cells treated with free doxorubicin showed, as expected, no significant internalization (Figure 5A).

**Figure 5:**
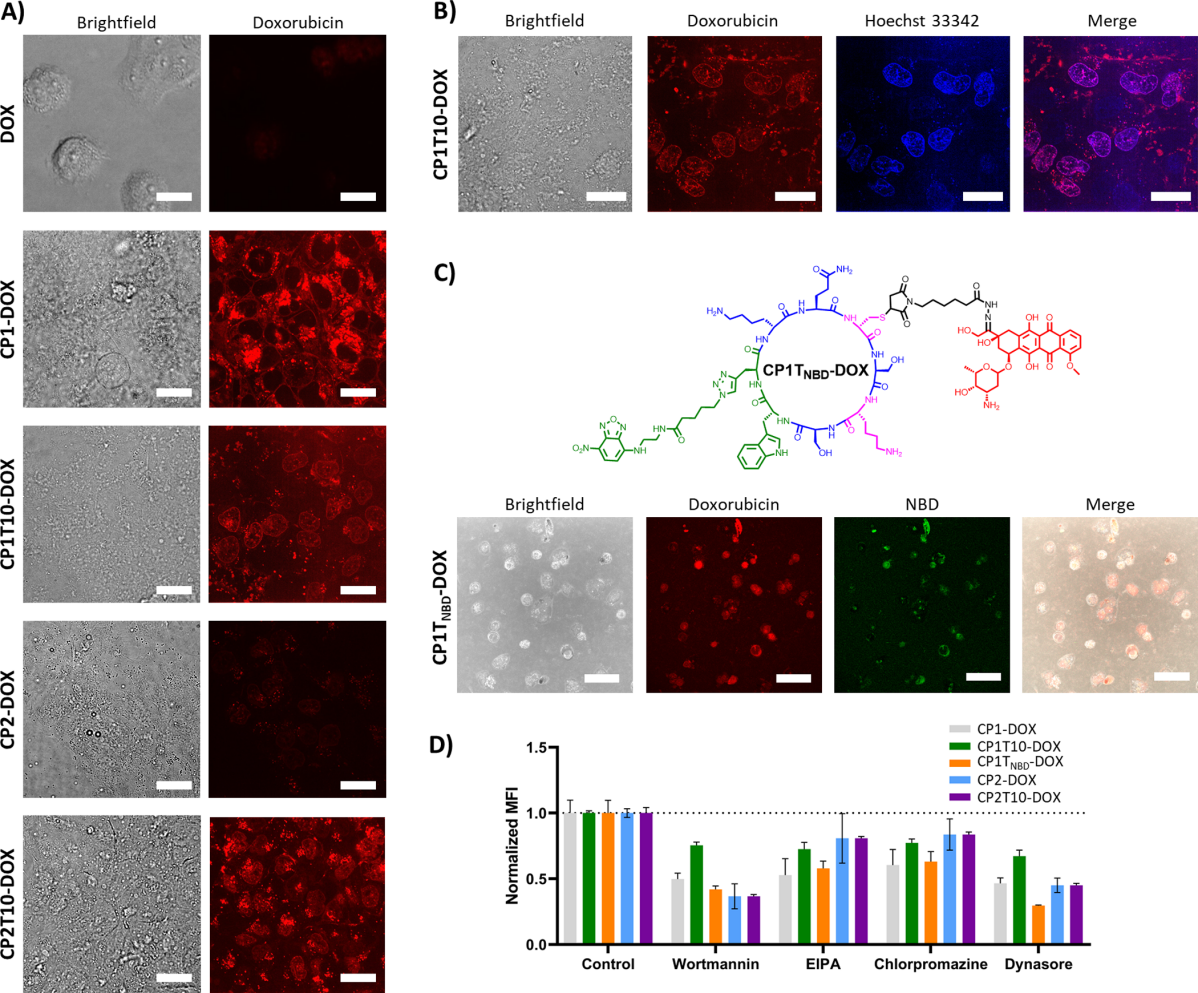
(A) Confocal images of NCI/ADR-RES cells incubated during 2 h with **DOX**, **CP1-DOX**, **CP1T10-DOX**, **CP2-DOX,** or **CP2T10-DOX** (50 μM). (B) Colocalization by confocal microscopy of **CP1T10-DOX** (50 μM) with the NCI/ADR-RES nucleus after incubation for 2 h. The nuclei of the cells were stained with Hoechst 33342. (C) Confocal images of the colocalization of DOX and NBD fluorescence when the cells NCI/ADR-RES were treated with **CP1T**_**NBD**_ **-DOX** (100 μM) for 2 h, with a calculated **Pearson correlation coefficient (PCC)** of 0.66 ± 0.01. (D) Internalization of peptide samples (50 μM) after incubation for 1 h when NCI/ADR-RES are treated with endocytosis inhibitors: Wortmannin (100 μM), EIPA (50 μM), Chlorpromazine (30 μM) or Dynasore (80 μM). The quantification was carried out by flow cytometry. MFI is the median fluorescence intensity. Cells treated only with the peptide and without inhibitors were used as controls. All scale bars are 25 μm.

Consequently, colocalization experiments were carried out to confirm the nuclear localization of the drug when the cells were incubated with the carbon tail derivatives. For this purpose, the cells were treated with the corresponding conjugate for 2 h, and subsequently, nuclei were stained with Hoechst 33342 (Figures 5B and S8). A confocal microscopy analysis of cells treated with hydrocarbon-tailed derivatives revealed partial nuclear colocalization of doxorubicin with the blue channel emission of the Hoechst reagent, although the drug (red channel) was also localized in other parts of the cells. This outcome supports that these derivatives facilitate drug delivery, possibly by stimulating their endosomal escape and overcoming the resistance of this cell line. In contrast, no such nuclear colocalization was observed with the chainless derivatives. Consequently, it can be inferred that active compounds not only prevent efflux pumps from expelling the drug from the cell, but also assist its transport to the nucleus by facilitating its exit from the endosomes as a species that cannot be recognized by the efflux pumps.

To elucidate the role of the cyclic peptide in this whole process, which includes internalization, transport to the nucleus, and drug release, the derivative containing the NBD dye attached at the end of the hydrophobic tail (**CP1T**_**NBD**_ **-DOX**, Figure 5C) was also studied. This derivative, which is visible in different channels, the green channel for NBD and the red for DOX, would allow the localization in a single experiment of both DOX and CP on their way to the nucleus to induce the biological function. As mentioned above, this derivative emerged as the most potent, exhibiting an IC_50_ of 3.7 μM after 72 h (Table 2). Incubation of NCI/ADR-RES cancer cells with this derivative was also carried out at different times (Figures 5C and S9), confirming that colocalization of both components (**PCC** ∼ 0.6) did not change over time. These coefficient values might suggest that part of the DOX is released and may be expelled or colocalized in the nucleus. These experiments demonstrated that both fluorescence emissions mostly coincided in the cell. The colocalization of CP and DOX emissions suggests that the drug remains mainly bound to the peptide during the entire route to the nucleus. To confirm this hypothesis and to clarify whether the drug was released under the more acidic conditions of the cellular environment of cancer cells or endosomes, the stability of **CP1-DOX** and **CP1T10-DOX** (200 μM) at pH 5.0 was addressed. The stability of these conjugates was followed over time, monitoring DOX release by mass spectrometry (Figures S10 and S11). In both cases, drug release was observed after 72 h under these conditions, although part of the cyclic peptide conjugate survived after this time. This is especially striking with **CP1T10-DOX**, in which the molecular ion corresponding to the drug is still very minor after this time. These results suggest greater stability when tubular aggregates are formed by locating the hydrazone bonds protected by alignment along the tube of the doxorubicin moieties. As expected, these conjugates are much more stable at physiological pH, with a small release of the drug after 72 h under these conditions. Overall, this result also suggests that the drug is only partially detached from the CP and that both drug and peptide remain bound throughout the pathway to the cell nucleus. This is most likely the way CP conjugation overcomes cellular resistance. In addition, no evidence of DOX release from CP to interact with the target DNA was found once the conjugate reaches the cell nucleus, as confirmed by the mentioned high PCC values.

Finally, we address the study of the internalization mechanism. For this purpose, cells were incubated with **CP1T**_**NBD**_ **-DOX** for 2 h, followed by the staining of lysosomes and endosomes with Lysotracker (Figure S12). This approach allowed us to observe the colocalization of the peptide fluorescence with endosomes and lysosomes (PCC = 0.54 ± 0.06), suggesting a modification of the drug entry mechanism from, among others, its characteristic passive diffusion to an endocytic pathway.^69^ It is worth mentioning that previously it has been reported that doxorubicin, due to its basic p*K*_a_, becomes sequestered in the acidic conditions of late-stage endosomes and lysosomes,^70^ something that does not seem to happen with our cyclic peptide conjugates. However, it should be noted that after this time, most of the CP is already localized in the nucleus. Cell uptake experiments on NCI/ADR-RES were also carried out. The uptake detected for cells treated with DOX was only 21.56%, while for those treated with **CP1T**_**NBD**_ **-DOX** was 56.34% (Figure S13). These results confirm that the higher intracellular levels of DOX in the cells treated with are related to the decrease in the DOX efflux, which is a characteristic of the NCI/ADR-RES cell line. Furthermore, the higher hydrolysis rates of tailless derivatives could also explain their lower activity in NCI/ADR-RES cells. These derivatives must be hydrolyzed in endosomes or lysosomes, allowing their cellular release and the expulsion of DOX by the P-gp protein, thus inactivating its antitumor activity in drug-resistant cell lines.

To confirm the endocytic mechanism, we quantified the internalization of the drug by flow cytometry in the presence of endocytosis inhibitors (Figure 5D). Endocytosis is a process that begins when endocytic coat proteins from the cytosol group together on the inner side of the plasma membrane, promoting membrane bending until a scission process provides a vesicle.^71^ Multiple different endocytic pathways have been described depending on the type of cytosolic proteins involved. Therefore, inhibition of these proteins should result in a reduction in CP internalization in cases where its entry is mediated by endocytosis. To this end, the inhibitors Wortmannin, EIPA, Chlorpromazine, and Dynasore were selected. For instance, Wortmannin is known to function as an inhibitor of macropinocytosis and clathrin-mediated endocytosis, EIPA prevents macropinocytosis, while Chlorpromazine is employed to inhibit the clathrin-mediated endocytosis, and finally, Dynasore is used to inhibit all the dynamin-dependent pathways.^72^ The results obtained from the analysis via flow cytometry revealed a substantial decrease in the internalization after 1 h for all inhibitors (Figure 5D), especially for Wortmannin and Dynasore. These results, when considered in conjunction with Lysotracker colocalization and the dotted pattern observed inside cancer cells by confocal microscopy, suggest a mechanism of internalization in which endocytosis plays a significant role, although partial internalization through membrane diffusion is not ruled out. We speculate that this endocytosis pathway together with the stability of the CP/DOX junction must prevent the action of the P-gp protein that causes doxorubicin resistance (Figure 6).^66,67^ Furthermore, the incorporation of the alkyl chain to the cyclic peptide facilitates its permeability through the membrane, helping with endosomal escape and, as a result, the transport of the drug to the nucleus. This endosome escape must follow a mechanism similar to the one proposed for the antimicrobial activity of the original cyclic peptides (**CP1Tn and CP2Tn**). After initial electrostatic interactions between the CP and the membrane, the hydrophobic tail starts to interact with the membrane, which is accompanied by a great distortion and final collapse of the membrane system, facilitating the escape of the conjugate.

**Figure 6:**
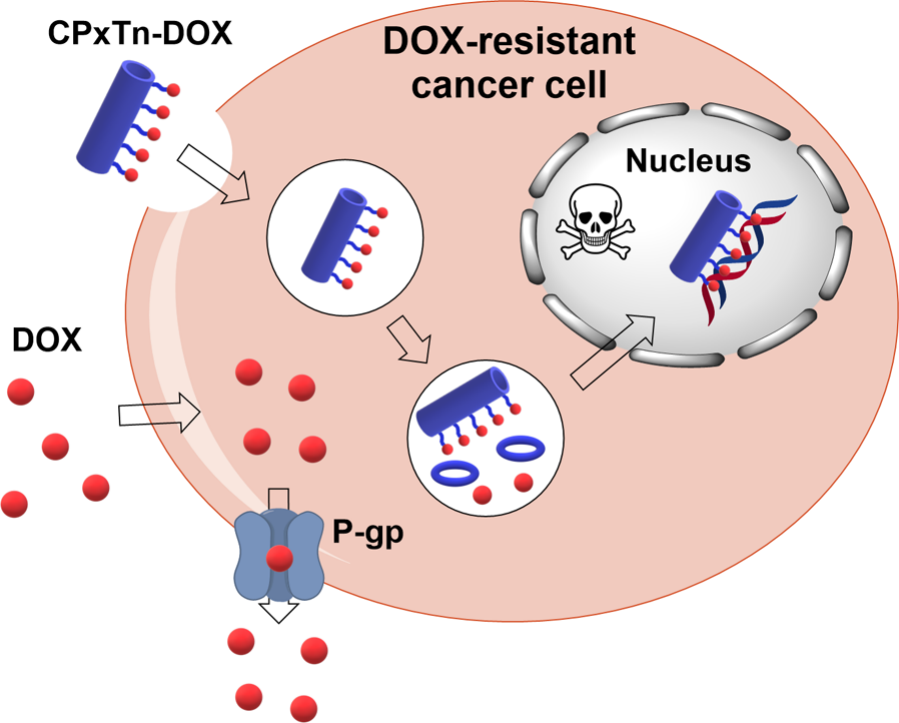
Proposed mechanism of action of the cyclic peptides containing doxorubicin studied in this work.

## Conclusions

In summary, we have shown that the use of membrane-active antimicrobial cyclic peptides conjugated to an anticancer drug such as doxorubicin provides a novel strategy for both the delivery and targeting of drug-resistant cancer cells. In this work, using different techniques, we have demonstrated the self-assembly properties of amphipathic cyclic peptides containing doxorubicin to form nanotubes, bundles, or 2D structures. Most likely, these structures are formed specifically in the presence of anionic membranes, which provide some selectivity for cancer cell lines due to the abnormal concentration of phosphatidylserine lipids in the outer leaflet of their membranes. These conjugates have been shown to modify the mechanism of drug internalization and transport to the cell nucleus, thus circumventing resistance mechanisms, such as efflux pumps. The internalization mechanism appears to be via endocytosis, where the membrane-disrupting properties of the cyclic peptide allow for endosomal escape. Remarkably, not only the drug but also the cyclic peptide reaches the cell nucleus. The synergistic effect of conjugation of the drug with the cyclic peptides is clearly demonstrated, which not only greatly enhances the cytotoxic effect of DOX in resistant cells but also makes its antineoplastic action more sustained over time. We hypothesize that this combined drug delivery therapy could open new opportunities for the treatment of drug-resistant cancers. Optimization of the peptide sequence should provide new derivatives with improved properties. The results obtained in this work suggest that the combination of these supramolecular drugs^73^ with other conventional ones could allow the development of new therapeutic tools that can be adapted to the intrinsic characteristics of tumors.

## Supplementary Material



## Notes

Figure S1 showing synthetic route to obtain the maleimide-based linker with doxorubicin; Figure S2 showing fluorescence spectra, critical aggregation concentration calculation and scanning transmission electron microscopy micrographs of solutions of **CP1**, **CP1T10**, **CP2,** and **CP2T10** in PBS; Figure S3 showing fluorescence and CD spectra and estimation of critical aggregation concentration of solutions of **DOX**, **CP1-DOX**, **CP1T10-DOX**, **CP2-DOX,** or **CP2T10-DOX** in PBS; Figure S4 showing STEM or TEM micrographs of samples of **CP1-DOX**, **CP1T10-DOX**, **CP2-DOX,** and **CP2T10-DOX** in different aqueous media or pH; Figure S5 showing AFM micropraphs of an aqueous solution of **CP2-DOX** deposited on mica with the corresponding height profiles; Figure S6 showing confocal images of NCI/ADR-RES cells at different incubation times and concentrations of **CP1-DOX**; Figure S7 showing confocal images of NCI/ADR-RES cells at different incubation times and concentrations of **CP2-DOX**; Figure S8 showing colocalization by confocal microscopy of **CP1-DOX**, **CP1T10-DOX**, **CP1T14-DOX**, **CP2-DOX**, **CP2T10-DOX,** and **CP2T14-DOX** with the NCI/ADR-RES nucleus after incubation for 2 h; Figure S9 showing confocal images of DOX and NBD fluorescence colocalization when the NCI/ADR-RES cells were treated with **CP1T**_**NBD**_ **-DOX** at different times; Figure S10 showing MS analysis for evaluating hydrazone hydrolysis of samples of **CP1-DOX** or **CP1T10-DOX** incubated at pH 5.0; Figure S11 showing time-course DOX release of **CP1-DOX** or **CP1T10-DOX** incubated at pH 5.0 or pH 7.0; Figure S12 showing confocal fluorescence colocalization images of NBD and LysoTracker Deep Red when NCI/ADR-RES cells were treated with **CP1T**_**NBD**_ **-DOX** for 2 h; Figure S13 showing RP-uHPLC analysis of the cell uptake experiments; Figure S14 showing the synthetic scheme for preparation of **NBD-N**_**3**_; Figure S15 showing the synthetic scheme for preparation of CPs; Figure S16 showing RP-uHPLC chromatogram and MS of **CP1**; Figure S17 showing ^1^H NMR of **CP1**; Figure S18 showing COSY of **CP1**; Figure S19 showing TOCSY of **CP1**; Figure S20 showing FT-IR spectrum of **CP1**; Figure S21 showing RP-uHPLC chromatogram and MS of **CP1T6**; Figure S22 showing FT-IR spectrum of **CP1T6**; Figure S23 showing RP-uHPLC chromatogram and MS of **CP1T10**; Figure S24 showing FT-IR spectrum of **CP1T10**; Figure S25 showing RP-uHPLC chromatogram and MS of **CP1T14**; Figure S26 showing FT-IR spectrum of **CP1T14**; S27 showing RP-uHPLC chromatogram and MS of **CP1T**_**NBD**_; Figure S28 showing FT-IR spectrum of **CP1T**_**NBD**_; Figure S29 showing RP-uHPLC chromatogram and MS of **CP2**; Figure S30 showing ^1^H NMR of **CP2**; Figure S31 showing COSY of **CP2**; Figure S32 showing TOCSY of **CP2**; Figure S33 showing FT-IR spectrum of **CP2**; Figure S34 showing RP-uHPLC chromatogram and MS of **CP2T6**; Figure S35 showing FT-IR spectrum of **CP2T6**; Figure S36 showing RP-uHPLC chromatogram and MS of **CP2T10**; Figure S37 showing FT-IR spectrum of **CP2T10**; Figure S38 showing RP-uHPLC chromatogram and MS of **CP2T14**, Figure S39 showing FT-IR spectrum of **CP2T14**; Figure S40 showing the procedure to obtain CP-DOX conjugates; Figure S41 showing the ESI-MS and FT-IR of **CP1-DOX**; Figure S42 showing the ESI-MS and FT-IR of **CP1T6-DOX**; Figure S43 showing the ESI-MS and FT-IR of **CP1T10-DOX**; Figure S44 showing the ESI-MS and FT-IR of **CP1T14-DOX**; Figure S45 showing the ESI-MS and FT-IR of **CP1T**_**NBD**_ **-DOX**; Figure S46 showing the ESI-MS and FT-IR of **CP2-DOX**; Figure S47 showing the ESI-MS and FT-IR of **CP2T6-DOX**; Figure S48 showing the ESI-MS and FT-IR of **CP2T10-DOX**; Figure S49 showing the ESI-MS and FT-IR of **CP2T14-DOX;** Table 1 showing summary of the critical aggregation concentrations (*cac*) of CPs; Table 2 showing FT-IR data of the different peptides; and Table 3 showing *in vitro* activity data in MRC-5 (human embryonic lung cell line) after 7 days incubation of different cyclic peptide-doxorubicin conjugates (PDF)
