# Supplementary material for: Self-Assembling Cyclic Peptide Nanotubes for the Delivery of Doxorubicin into Drug-Resistant Cancer Cells
Source: ACS Appl Mater Interfaces. 2025 Aug 28;17(36):50191–202. doi: 10.1021/acsami.5c05264 (PMC12442009; doi:10.1021/acsami.5c05264)
Supplement: Supplementary file 1 [file am5c05264_si_001.pdf]

SUPPORTING INFORMATION

Self-Assembling Cyclic Peptide Nanotubes for the Delivery of Doxorubicin into Drug-resistant cancer cells

Marcos Vilela-Picos,[a] Eva González-Freire,[a] Federica Novelli,[a] Yeray Folgar-Cameán,[a] José Brea,[b] Manuel Amorín,[a] Juan R. Granja[a,*]

[a] Centro Singular de Investigación en Química Biolóxica e Materiais Moleculares (CiQUS), Departamento de Química Orgánica, Universidade de Santiago de Compostela, 15782, Santiago de Compostela, Spain

[b] Centro Singular de Investigación en Medicina Molecular e Enfermidades Crónicas (CiMUS), Departamento de Farmacología, Farmacia y Tecnología Farmacéutica. Facultad de Farmacia, Universidade de Santiago de Compostela, 15782, Santiago de Compostela, Spain

* Email: juanr.granja@usc.es

Table of contents:

1. Supporting figures (Fig. S1-S13) S3-S11

   Supporting tables (Tables S1-S3) S12

2. Synthesis S13-S43

   2.1 Synthesis of the maleimide-based doxorubicin linker S13-S14

   2.2 Synthesis of NBD-N_3 .... S14-S15

   2.3 Solid-phase peptide synthesis S16-S34

   2.4 Maleimide-thiol doxorubicin conjugation S34-S43

3. References S44

1. Supporting figures and tables (Fig. S1-S13 and Tables S1-S3)

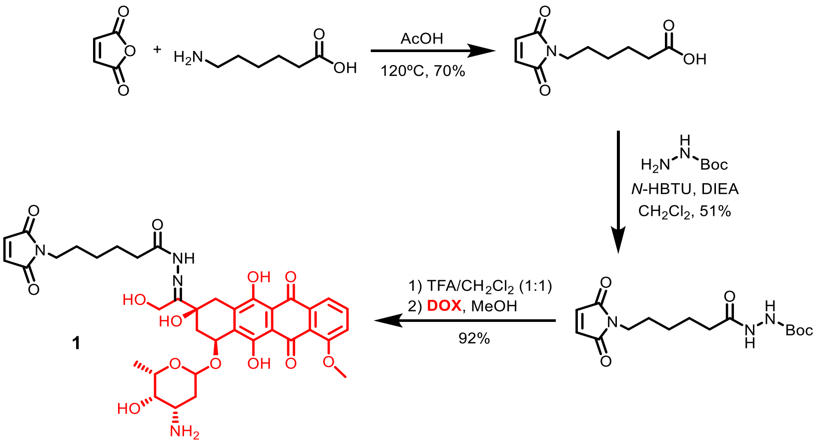

Figure S1. Synthetic route for obtaining the maleimide-based linker with doxorubicin incorporated through a hydrazone bond (**1**).

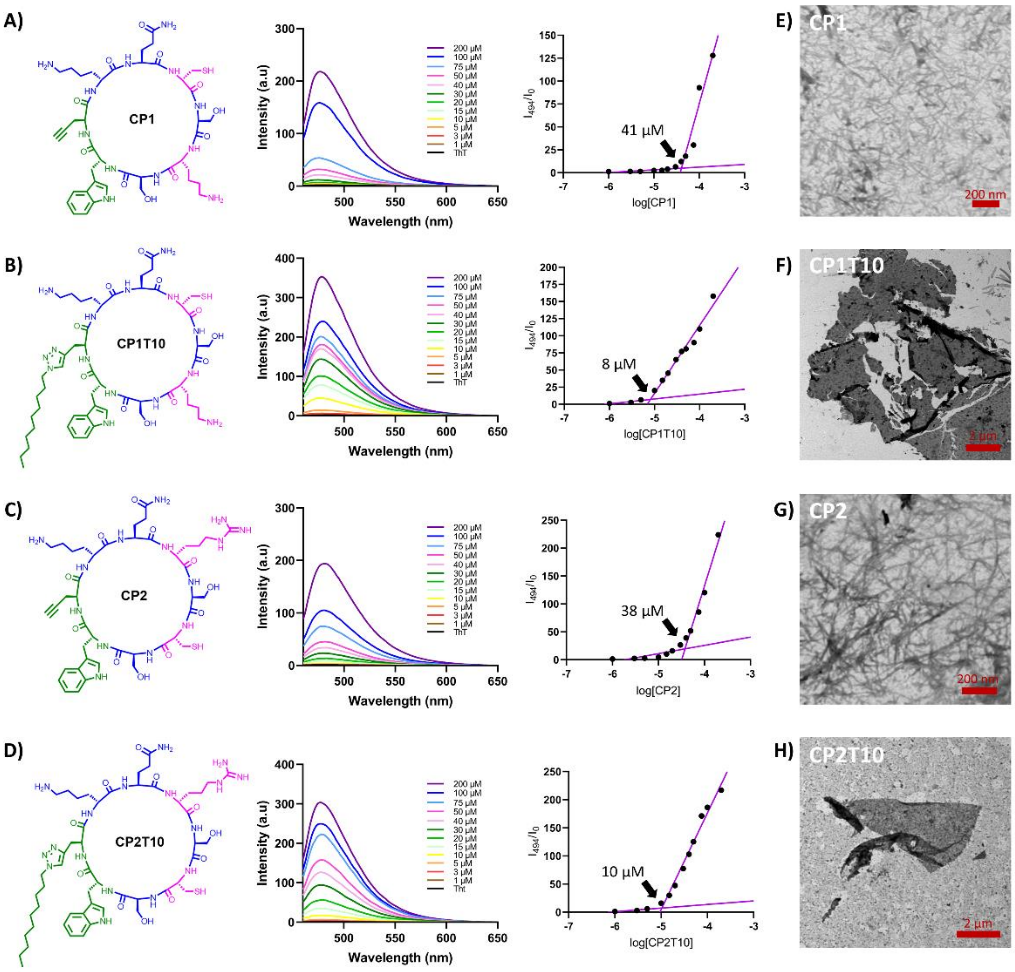

Figure S2. (A-D) ThT (20 µM) fluorescence spectra at different concentrations (λ_{ex} = 440 nm) and critical aggregation concentrations (*cac*) of solutions of (A) **CP1**,

(B) **CP1T10**, (C) **CP2** or (D) **CP2T10** in PBS (10 mM, 107 mM NaCl, pH 7.4). (E-H) Scanning transmission electron microscopy micrographs of (E) **CP1**, (F) **CP1T10**, (G) **CP2** or (H) **CP2T10** at 200 µM and pH 7.0.

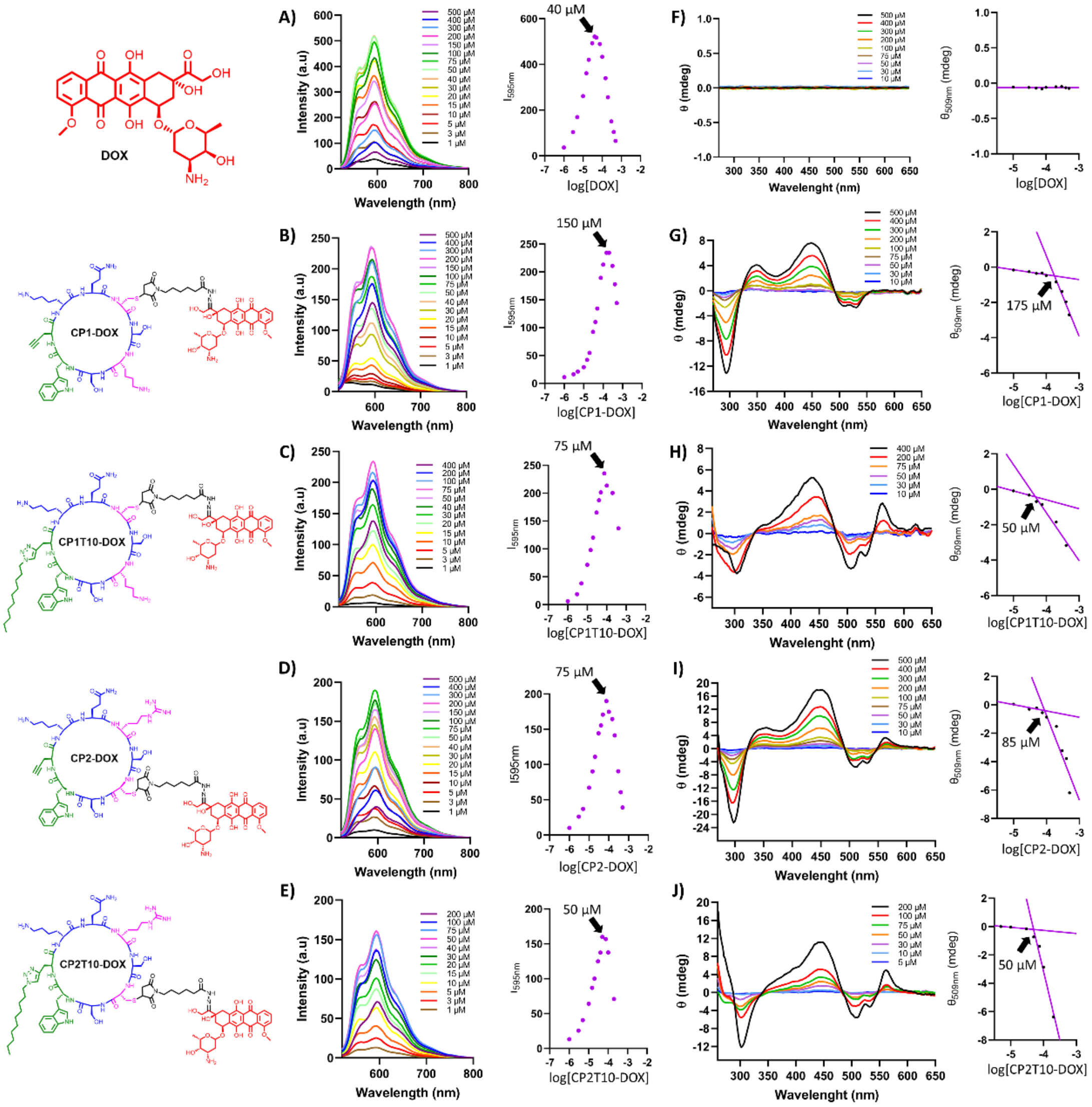

Figure S3. (A-E) Fluorescence spectra of solutions of (A) **DOX**, (B) **CP1-DOX**, (C) **CP1T10-DOX**, (D) **CP2-DOX** or (E) **CP2T10-DOX** in PBS (10 mM, 107 mM NaCl, pH 7.4) at different peptide concentrations (λ_{ex} = 480 nm) and determination of the corresponding critical aggregation concentration (*cac*). (F-J) CD spectra of (F) **DOX**, (G) **CP1-DOX**, (H) **CP1T10-DOX**, (I) **CP2-DOX** or (J) **CP2T10-DOX** in PBS (10 mM, 107 mM NaCl, pH 7.4) at different concentrations and determination of the corresponding critical aggregation concentration (*cac*).

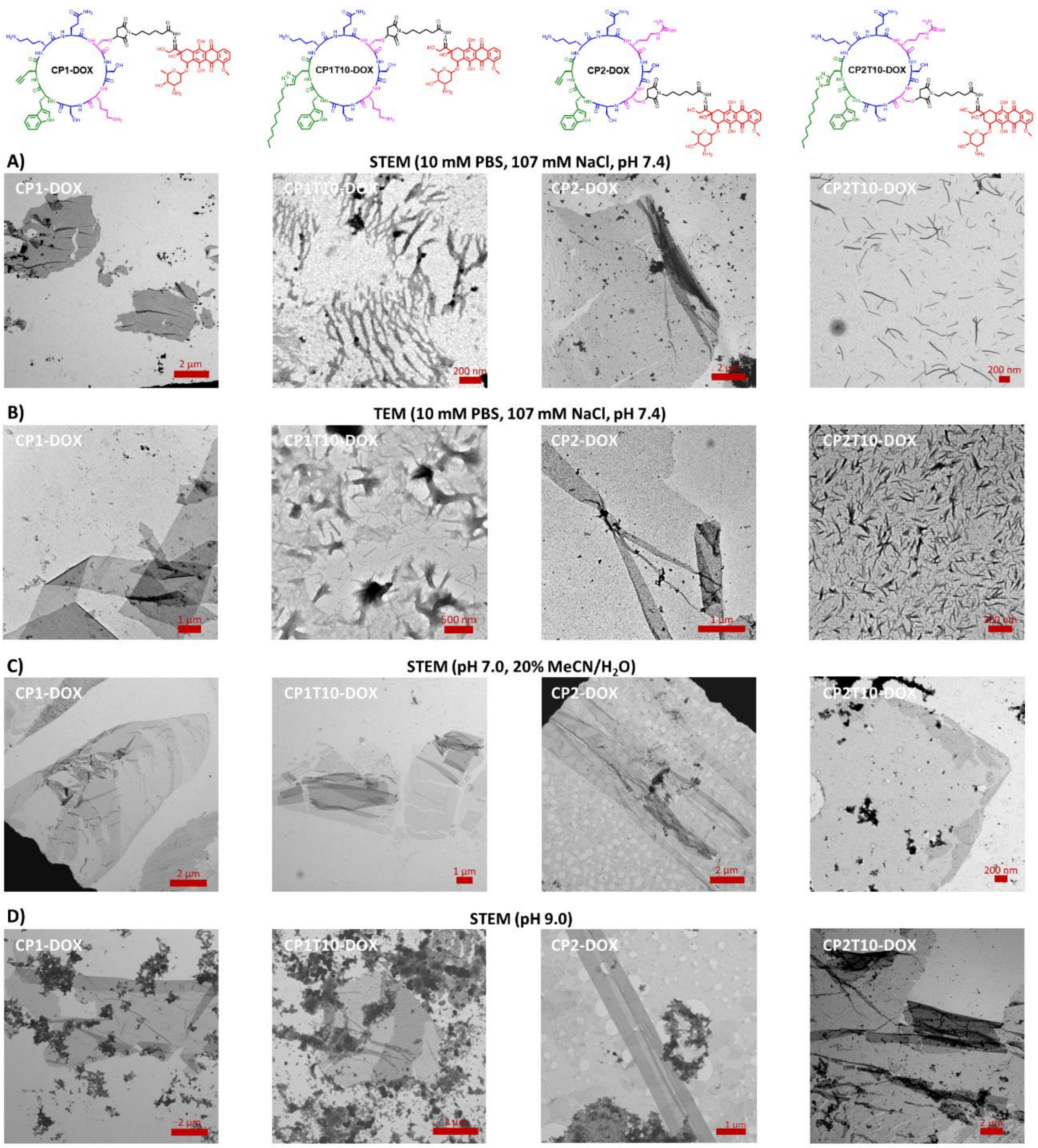

Figure S4. STEM or TEM micrographs of 200 µM samples of **CP1-DOX**, **CP1T10-DOX**, **CP2-DOX** and **CP2T10-DOX** in (A and B) PBS (10 mM, 107 mM NaCl) at pH 7.4, (C) pH 7.0 in 20% MeCN/H_2O and (D) an aqueous solution at pH 9.0.

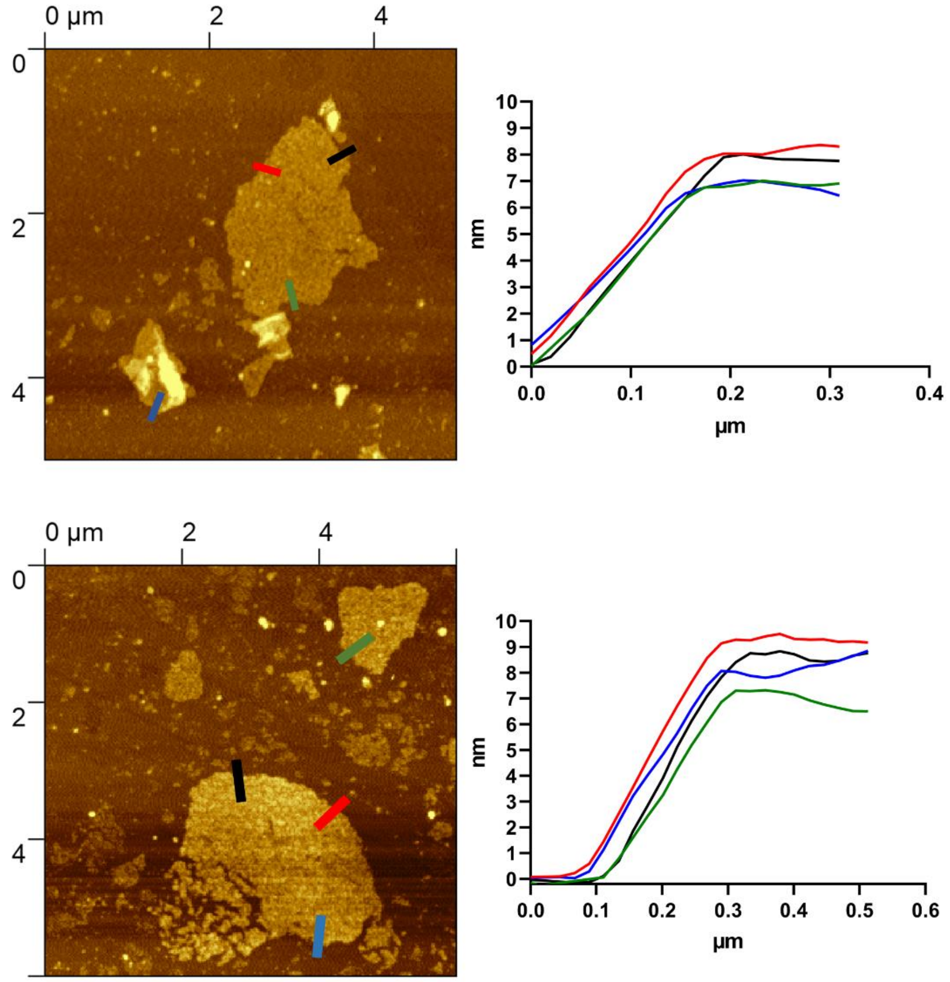

Figure S5. AFM images of samples derived from a solution of **CP2-DOX** (200 μM) in PBS (10 mM, 107 mM NaCl, pH 7.4) deposited on mica. The height profiles were obtained along the colour lines indicated in the image.

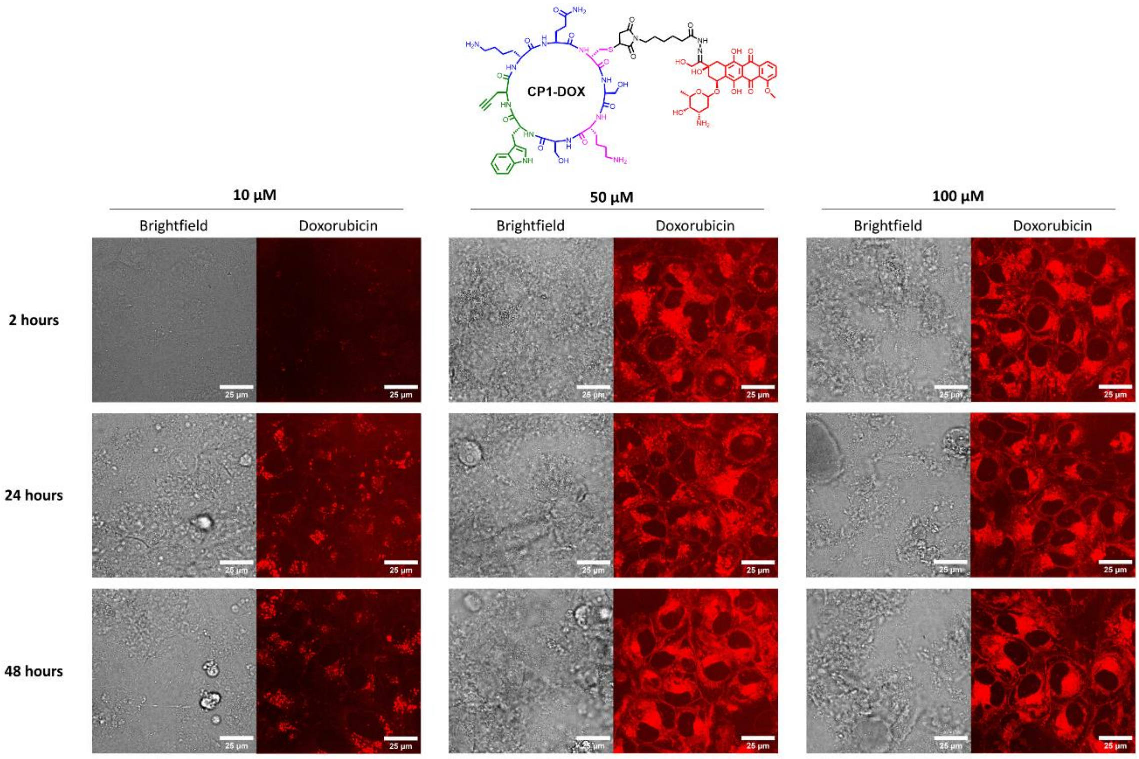

Figure S6. Confocal images of NCI/ADR-RES cells incubated with **CP1-DOX** at different incubation times and peptide concentrations. Scale bars: 25 µm.

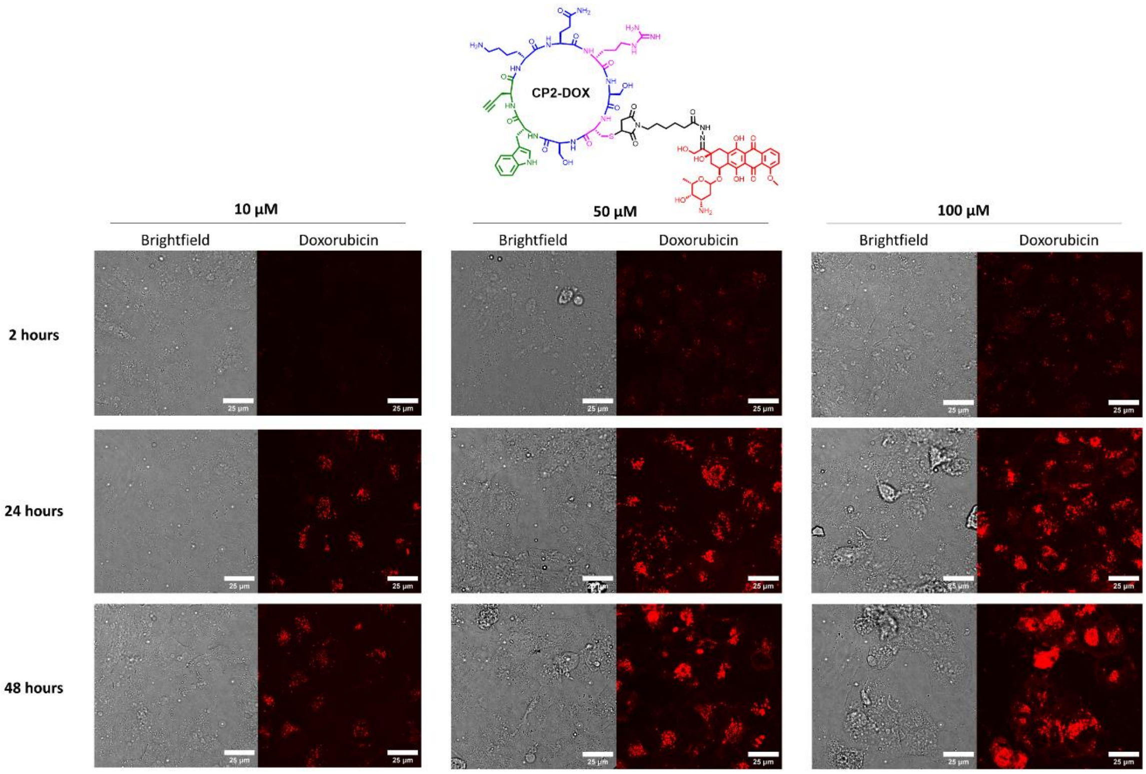

Figure S7. Confocal images of NCI/ADR-RES cells incubated with **CP2-DOX** at different incubation times and peptide concentrations. Scale bars: 25 µm.

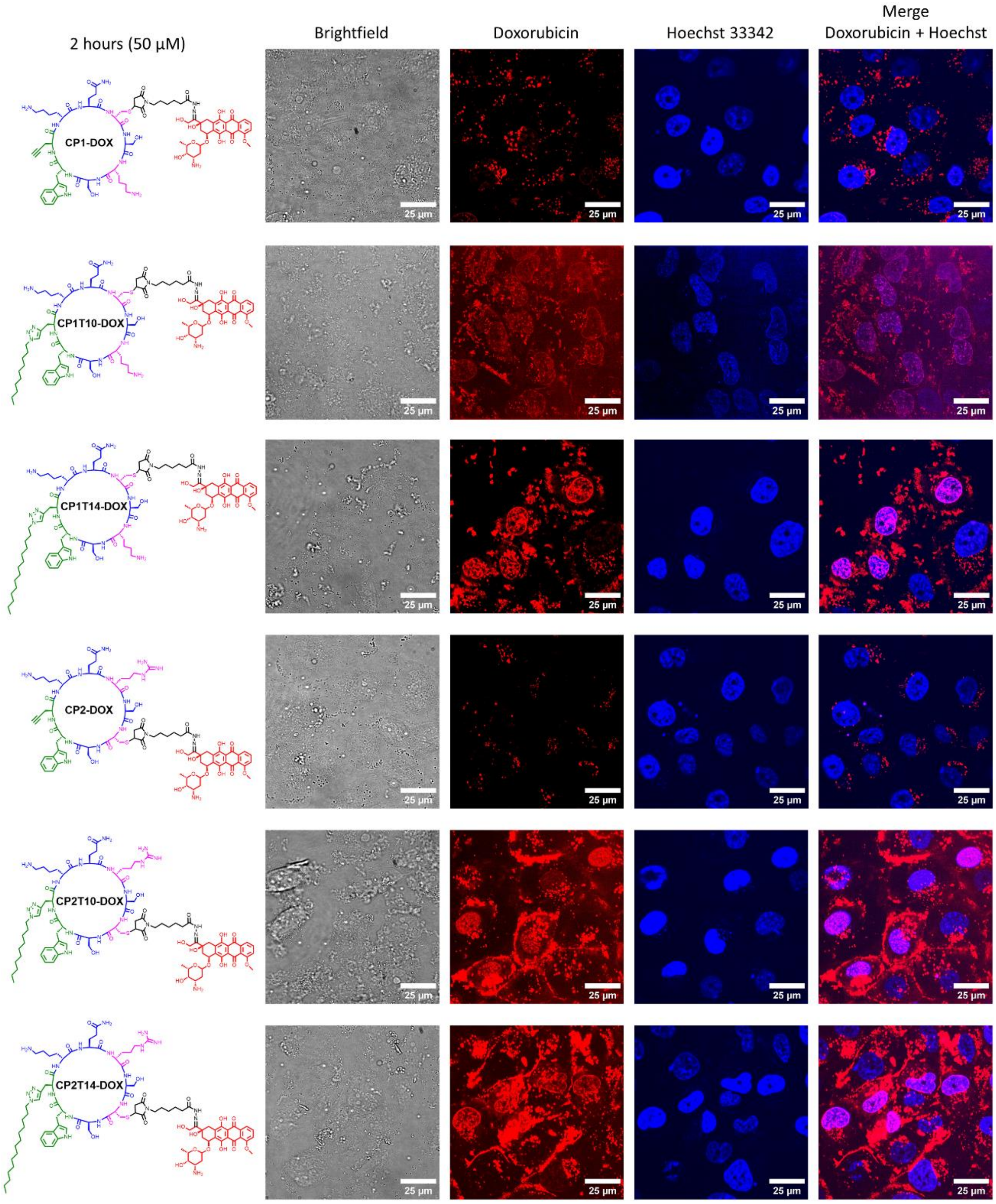

Figure S8. Colocalization by confocal microscopy of **CP1-DOX**, **CP1T10-DOX**, **CP1T14-DOX**, **CP2-DOX**, **CP2T10-DOX** and **CP2T14-DOX** with the NCI/ADR-RES nucleus after incubation for 2 hours at a peptide concentration of 50 µM. The nuclei of the cells were stained with Hoechst 33342. Scale bars: 25 µm.

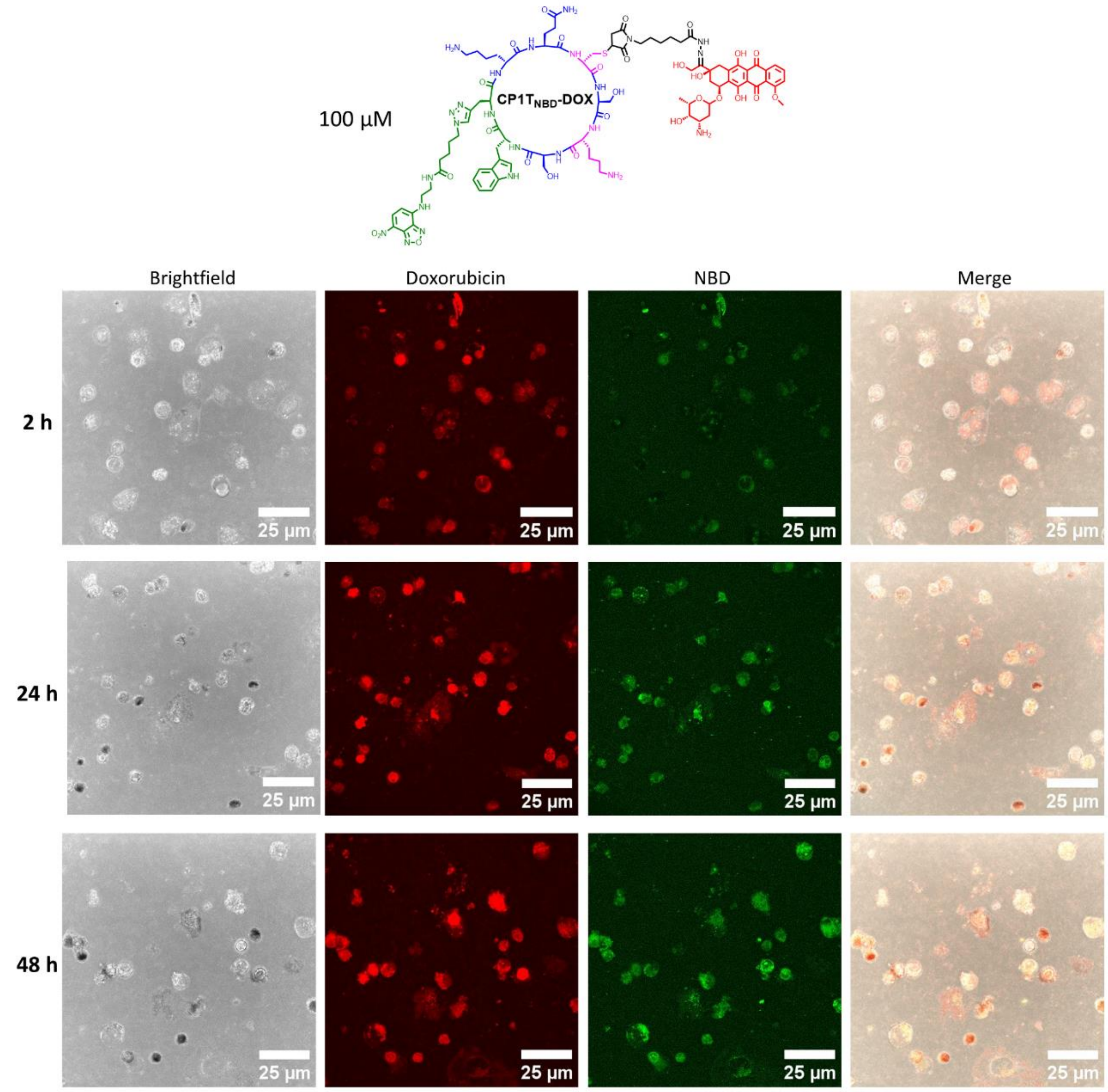

Figure S9. Confocal images of DOX and NBD fluorescence colocalization when NCI/ADR-RES cells are treated with **CP1T$_{NBD}$-DOX** at a concentration of 100 µM for 2, 24 or 48 hours, providing Pearson correlation coefficients (**PCC**) of 0.66±0.01, 0.61±0.11, 0.62±0.02, respectively. Scale bars: 25 µm.

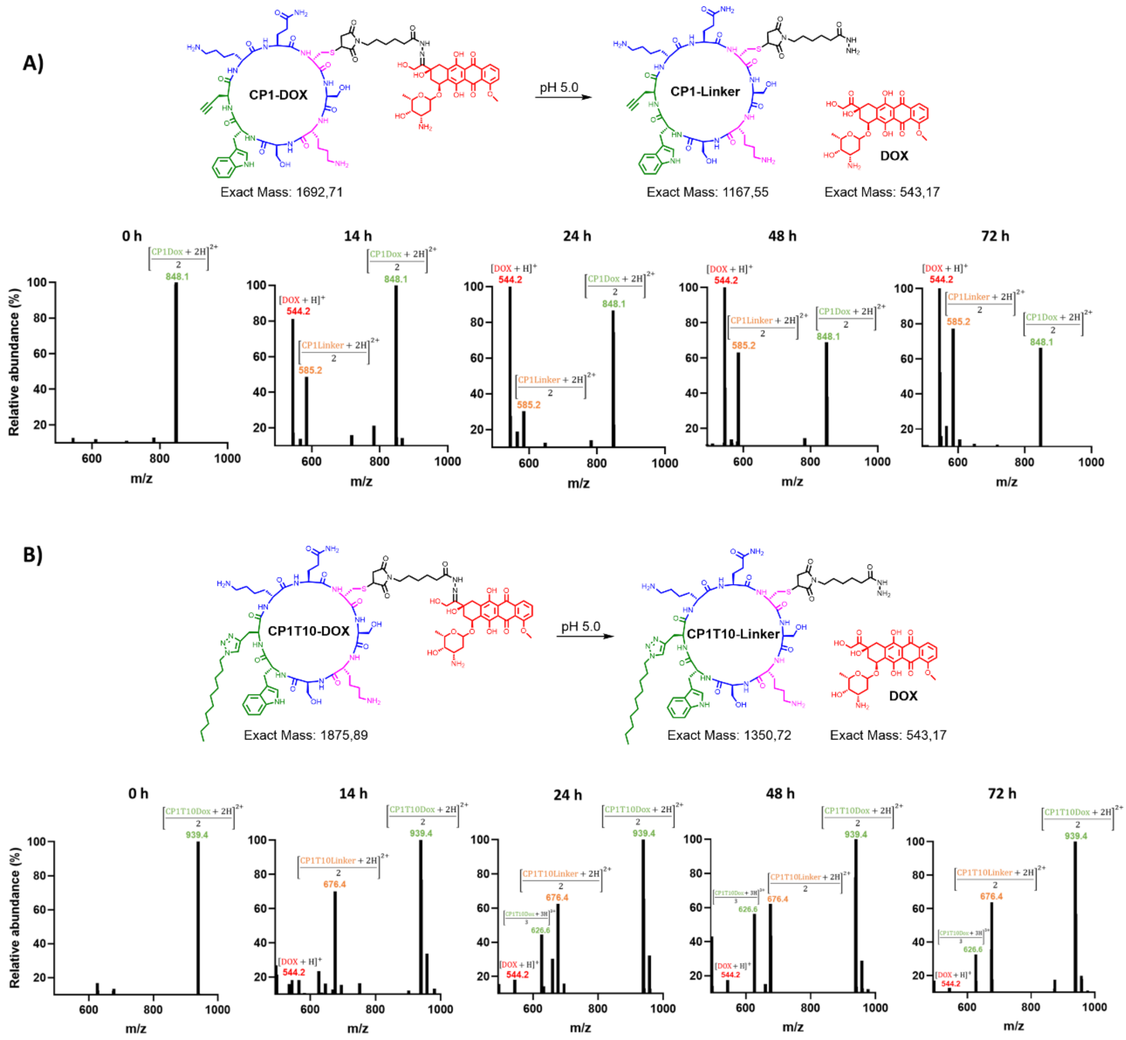

Figure S10. MS analysis of an aqueous solution incubated at pH 5.0 of (A) **CP1-DOX** or (B) **CP1T10-DOX** at 200 µM.

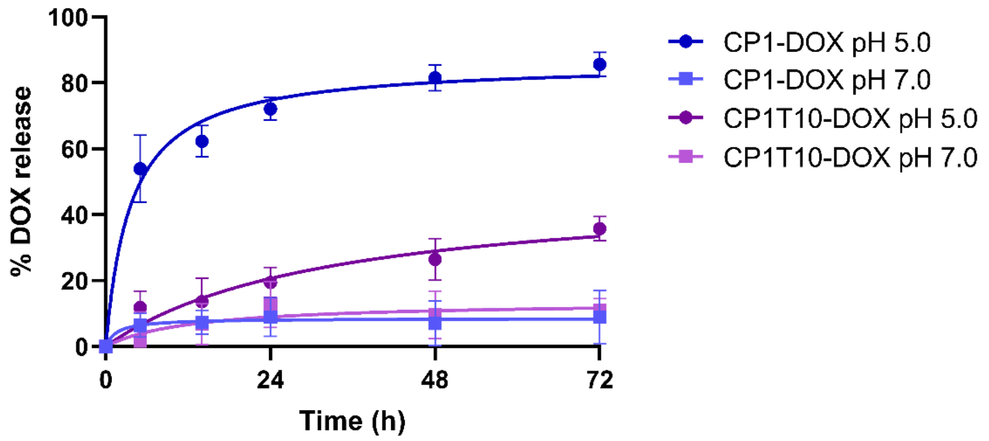

Figure S11. Time-course DOX release of **CP1-DOX** or **CP1T10-DOX** (200 µM) incubated at pH 5.0 (10 mM Citrate buffer) or pH 7.0 (H_2O).

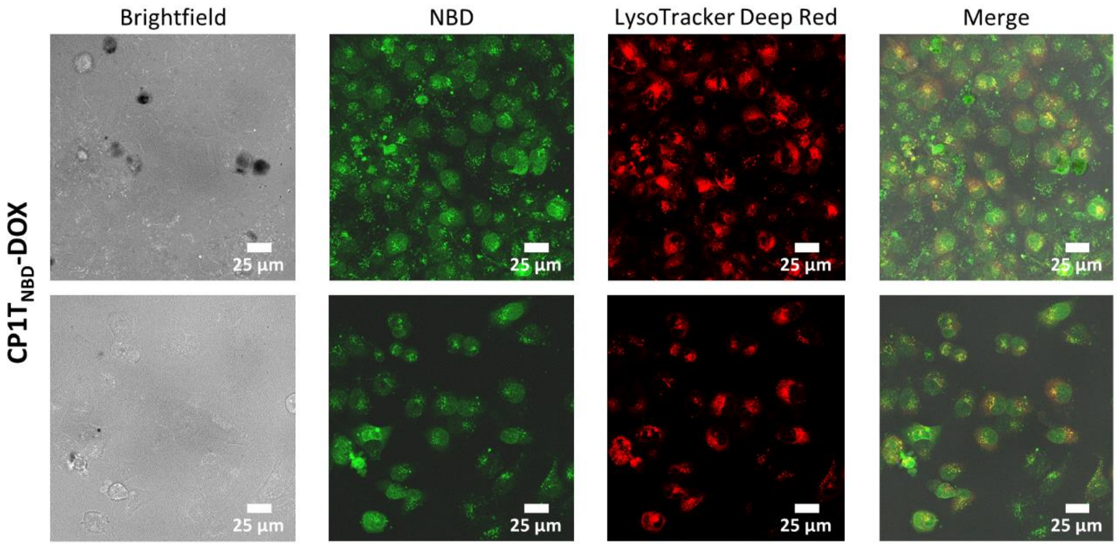

Figure S12. Confocal fluorescence colocalization images of NBD and LysoTracker Deep Red when NCI/ADR-RES cells are treated with **CP1T$_{NBD}$-DOX** at a concentration of 100 µM for 2 hours, from which a PCC of 0.54±0.06 was calculated. Scale bars: 25 µm.

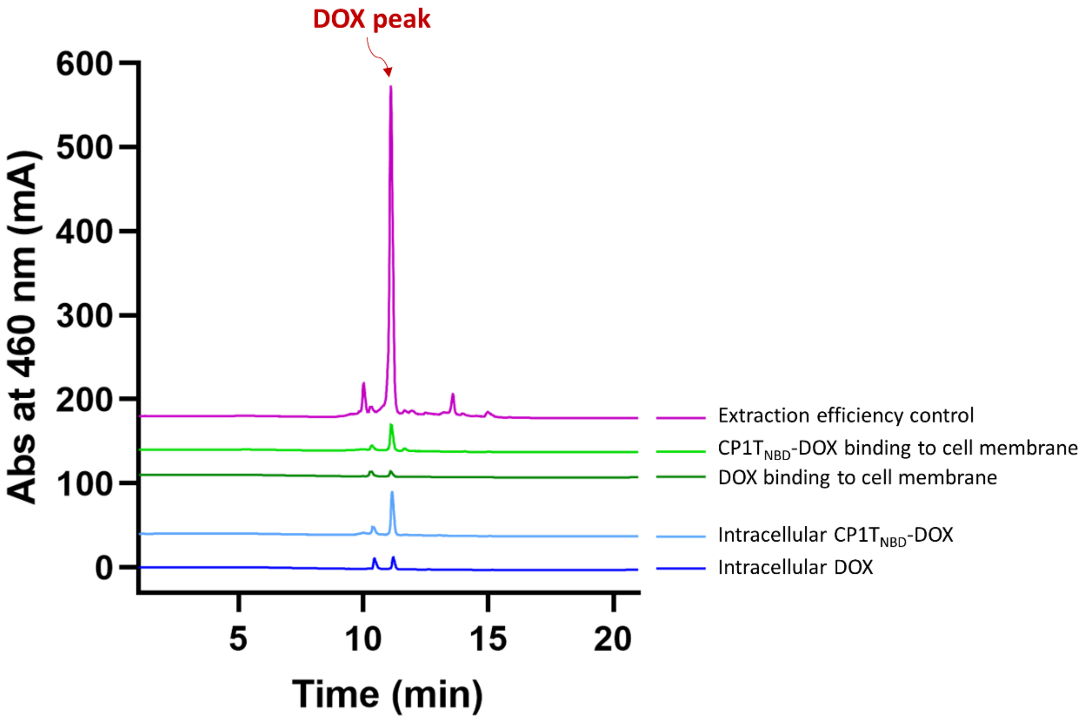

Figure S13. RP-uHPLC analysis of the cell uptake experiments [Agilent SB-C18 column, H_2O (0.1% TFA)/MeCN (0.1% TFA), 100:0 →100:0 (2 min) and 100:0 → 5:95 (21 min), $\lambda_{detection}$ = 460 nm], R_t (DOX) = 11.1 min. Samples were dissolved in H_2O +0.1% TFA before injection to ensure that all DOX was released from the peptide.

CP	**cac** (µM)
**CP1**	41
**CP1-DOX**	150-175
**CP1T10**	8
**CP1T10-DOX**	50-75
**CP2**	38
**CP2-DOX**	75-85
**CP2T10**	10
**CP2T10-DOX**	50

Table S1. Summary table of the critical aggregation concentration (*cac*) of CPs.

	Frequency (cm^{-1})			
**CP**	Amide A	Amide I_a	Amide I_b	Amide II
**CP1-DOX**	3270.3	1629.4	1670.8	1540.6
**CP1T6-DOX**	3270.3	1625.4	1670.8	1536.7
**CP1T10-DOX**	3274.2	1627.4	1672.8	1540.6
**CP1T14-DOX**	3272.3	1629.4	1670.8	1536.7
**CP1T$_{NBD}$-DOX**	3274.2	1627.4	1672.8	1536.7
**CP2-DOX**	3276.2	1627.4	1670.8	1538.7
**CP2T6-DOX**	3272.3	1627.4	1668.8	1538.7
**CP2T10-DOX**	3272.3	1625.4	1670.8	1538.7
**CP2T14-DOX**	3270.3	1629.4	1668.8	1534.7

Table S2. Summary table of peptide FT-IR data.

	MRC-5 (7 days)	
**Compound**	*Emax* (%inhibition)	*IC$_{50}$* (µM)
**CP1-DOX**	89 ± 3	6.5 ± 0.3
**CP1T10-DOX**	91 ± 2	2.2 ± 0.1
**CP1T$_{NBD}$-DOX**	93 ± 1	4.7 ± 0.1
**CP2-DOX**	90 ± 3	3.7 ± 0.2
**CP2T10-DOX**	92 ± 3	4.3 ± 0.3
**Linker-DOX**	89 ± 3	2.5 ± 0.2
**DOX**	87 ± 1	1.5 ± 0.1
**Cisplatin**	93 ± 3	6.2 ± 0.1

Table S3. *In vitro* activity in MRC-5 (human embryonic lung cell line) after incubation for 7 days of different cyclic peptide-doxorubicin conjugates. *Emax* corresponds to the maximum effect, which is the inhibition obtained at the highest concentration tested (100 µM). *IC$_{50}$* is the concentration at which 50% growth inhibition is obtained. The *IC$_{50}$* was only calculated for those compounds with an *Emax* greater than 50%.

2. Synthesis

2.1. Synthesis of the maleimide-based doxorubicin linker (1). The synthetic route to obtain the maleimide-based doxorubicin linker (**1**) is illustrated in Figure S1.

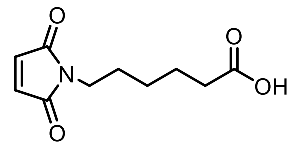

6-(2,5-dihydro-2,5-dioxo-1*H*-pyrrol-1-yl)hexanoic acid (6-maleimidocaproic acid). A solution of maleic anhydride (1.44 g, 14.64 mmol) and 6-aminocaproic acid (1.60 g, 12.20 mmol) in acetic acid glacial (40 mL) was refluxed for 6 hours at 120ºC. Then, the reaction mixture was diluted with H_2O and extracted with AcOEt (3 x 20 mL). The organic phase was dried over anhydrous $MgSO_4$, filtered and concentrated under reduced pressure. The resulting residue was purified by flash chromatography (5-10% $MeOH/CH_2Cl_2$) to afford the product as a crystalline white solid [1.88 g, 70%, R_f = 0.61 (10% $MeOH/CH_2Cl_2$)]. **^{1}H NMR** ($CDCl_3$, 300 MHz, δ): 11.30 (br, 1H, COOH), 6.65 (s, 2H, CH=CH), 3.46 (t, *J* = 7.2 Hz, 2H, N-CH_2), 2.29 (t, *J* = 7.4 Hz, 2H, CH_2-CO), 1.57 (m, 4H, 2 CH_2), 1.29 (m, 2H, CH_2). **^{13}C NMR** ($CDCl_3$, 75 MHz, δ): 179.7 (CO), 170.9 (CO), 134.1 (CH), 37.6 (CH_2), 33.8 (CH_2), 28.1 (CH_2), 26.1 (CH_2), 24.1 (CH_2). **ESI-MS** *m/z* (%): 234.1 (100) $[M+Na]^+$, 212.1 (46) $[M+H]^+$. **HRMS (ESI)** *m/z*: $[M+Na]^+$ calculated for $C_{10}H_{13}NNaO_4$: 234.0737, found 234.0736. The characterization data are consistent with those reported in the literature.[1,2]

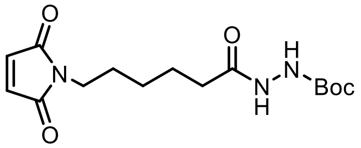

tert-butyl 2-(6-(2,5-dihydro-2,5-dioxo-1*H*-pyrrol-1-yl) hexanoyl)hydrazinecarboxylate. A solution of 6-maleimidocaproic acid (826 mg, 3.91 mmol) in CH_2Cl_2 (40 mL) was treated with *N*-HBTU (1.85 g, 4.89 mmol), DIEA (2.7 mL, 15.64 mmol) and *tert*-butyl carbazate (517 mg, 3.91 mmol). The reaction mixture was stirred for 1.5 hours under argon atmosphere and then washed with aqueous solutions of HCl (5%, 3 x 20 mL) and $NaHCO_3$ (sat., 3 x 20 mL). Then, the organic phase was dried over anhydrous $MgSO_4$, filtered and concentrated under vacuum. The residue was purified by flash chromatography (1-3% $MeOH/CH_2Cl_2$) to afford the product as a light-yellow oil [645 mg, 51%, R_f = 0.53 (5% $MeOH/CH_2Cl_2$)]. **^{1}H NMR** ($CDCl_3$, 300 MHz, δ): 7.56 (br, 1H, NH), 6.67 (s, 2H, CH=CH), 6.58 (br, 1H, NH), 3.50 (t, *J* = 7.2 Hz, 2H, N-CH_2), 2.20 (t, *J* = 7.5 Hz, 2H, CH_2-CO), 1.76-1.51 (m, 4H, 2 CH_2), 1.45 (s, 9H, Boc), 1.30 (m, 2H, CH_2). **^{13}C NMR** ($CDCl_3$, 75 MHz, δ): 172.4 (CO), 170.5 (CO), 155.5 (CO), 133.7 (CH), 80.7 (C), 38.2 (CH_2), 37.1 (CH_2), 33.1 (CH_2), 27.7 (CH_3), 25.8 (CH_2), 24.3 (CH_2). **ESI-MS** *m/z* (%): 348.2 (100) $[M+Na]^+$, 226.2 (23) $[M+H\text{-}Boc]^+$. **HRMS (ESI)** *m/z*: $[M+H]^+$ calculated for $C_{15}H_{24}N_3O_5$: 326.1710, found 326.1710. The characterization data are consistent with those reported in the literature.[1]

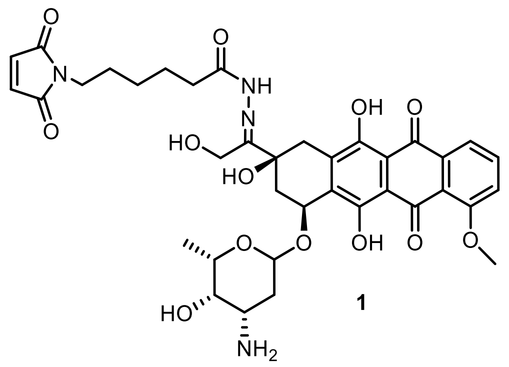

(6-maleimidocaproyl)hydrazone derivative of doxorubicin (1). A solution of tert-butyl 2-(6 (2,5-dihydro-2,5-dioxo-1*H*-pyrrol-1-yl)hexanoyl)hydrazinecarboxylate (411 mg, 1.26 mmol) in CH_2Cl_2 (10 mL) was treated with TFA (10 mL) and stirred for 15 minutes. After this time, the solvent was removed under reduced pressure and the resulting oil was redissolved in CH_2Cl_2 and further evaporated, this process was repeated three times. Then, Et_2O was added three times, concentrating after each time, and dried at high vacuum for 2 hours. The residue was dissolved in dry MeOH (140 mL) and treated with doxorubicin hydrochloride (244 mg, 0.420 mmol). The reaction mixture was stirred for 24 hours under argon atmosphere, room temperature and protected from light using aluminum foil. Once the reaction was completed, the volume was reduced at the rotary evaporator to approximately 1-2 mL and poured over ethyl acetate (40 mL). The red precipitate obtained was collected by centrifugation and washed with ethyl acetate (3 x 40 mL), centrifuging after each time. Finally, it was dried under high vacuum to obtain the product as a red solid [290 mg, 92%]. **^{1}H NMR** (DMSO-d_6, 500 MHz, δ): 14.08 (s, 1H, OH), 13.31 (s, 1H, OH), 10.29 (s, 1H, -N-NH-CO), 7.91 (m, 2H, H_{Ar}), 7.81 (bs, 2H, NH_2), 7.66 (m, 1H, H_{Ar}), 6.97 (s, 2H, CH=CH), 5.75 (t, *J* = 4.8 Hz, 1H, -O-CH-O- of sugar), 5.48 (s, 2H, CH_2-N=), 4.98 (m, 1H, CH), 4.42 (m, 2H, CH_2 sugar), 4.24-4.02 (m, 1H, CH sugar), 4.00 (s, 3H, OCH_3), 3.58 (m, 1H, CH sugar), 3.40 (m, 2H, CH_2 aliphatic ring), 3.31 (m, 1H, CH sugar), 3.25 (m, 2H, CH_2 aliphatic chain), 2.30-1.85 (m, 4H, CH_2 aliphatic chain and CH_2 aliphatic ring), 1.80-1.45 (m, 2H, CH_2 aliphatic chain), 1.32 (m, 2H, CH_2 aliphatic chain), 1.18 (m, 3H, CH_3 sugar), 1.04 (m, 2H, CH_2 aliphatic chain). **ESI-MS** *m/z* (%): 773.3 (10) $[M+Na]^+$, 751.3 (100) $[M+H]^+$. **HRMS (ESI)** *m/z*: $[M+H]^+$ calculated for $C_{37}H_{43}N_4O_{13}$: 751.2821, found 751.2821. The characterization data are consistent with those reported in the literature.[1]

2.2. Synthesis of the NBD-N3 tail. The synthetic route to obtain the azide derivative for the incorporation of the NBD chromophore follows the next scheme:

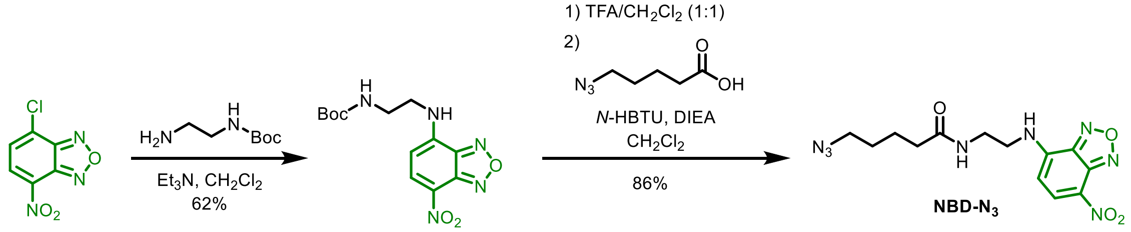

Figure S14. Synthetic strategy to obtain the **NBD-N3** tail.

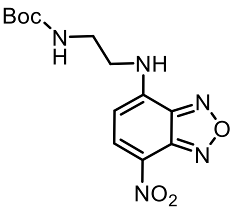

tert-butyl *N*-[2-(7-nitrobenzofuran-4-ylamino)ethyl]carbamate. A solution of *N*-Boc-ethylenediamine (194 mg, 1.21 mmol) and Et_3N (168 µL, 1.21 mmol) in CH_2Cl_2 (2 mL) was added to a solution of 4-chloro-7-nitrobenzofuran (219 mg, 1.10 mmol) in CH_2Cl_2 (6 mL). The reaction mixture was protected from light using aluminum foil and stirred for 18 hours under argon atmosphere. Then, the mixture was diluted with CH_2Cl_2 and washed with $NaHCO_3$ (sat., 3 x 20 mL). The organic phase was dried over anhydrous $MgSO_4$, filtered and concentrated under reduced pressure. The resulting residue was purified by flash chromatography (1-2% MeOH/CH_2Cl_2) and the product was obtained as a brown foam [221 mg, 62%, R_f = 0.55 (5% MeOH/CH_2Cl_2)]. **^{1}H NMR** ($CDCl_3$, 300 MHz, δ): 8.43 (d, *J* = 8.7 Hz, 1H, H_{Ar}), 7.78 (br, 1H, NH), 6.15 (d, *J* = 8.7 Hz, 1H, H_{Ar}), 5.26 (br, 1H, NH), 3.61 (m, 4H, 2 CH_2), 1.44 (s, 9H, Boc). **^{13}C NMR** ($CDCl_3$, 75 MHz, δ): 157.8 (CO), 144.5 (C), 144.4 (C), 144.3 (C), 144.1 (C), 136.4 (CH), 98.6 (CH), 81.1 (C), 46.1 (CH_2), 39.4 (CH_2), 28.4 (CH_3). **ESI-MS** *m/z* (%): 346.1 (100) $[M+Na]^+$, 324.1 (44) $[M+H]^+$. **HRMS (ESI)** *m/z*: $[M+H]^+$ calculated for $C_{13}H_{18}N_5O_5$: 324.1302, found 324.1304. The characterization data are consistent with those reported in the literature.[3]

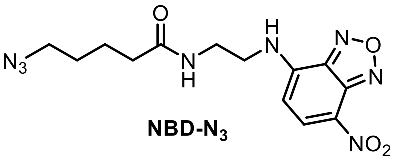

5-azido-N-(2-((7-nitrobenzo[c][1,2,5]oxadiazol-4-yl)amino)ethyl)pentanamide. A solution of tert-butyl *N*-[2-(7-nitrobenzofuran-4-ylamino)ethyl]carbamate (96 mg, 0.297 mmol) in CH_2Cl_2 (2.5 mL) was treated with TFA (2.5 mL) and stirred for 15 min. After the solvent was removed under reduced pressure, the oil obtained was redissolved in CH_2Cl_2 and further evaporated, the process was repeated three times. The residue was dried at high vacuum for 2 hours. The resulting TFA salt was dissolved under argon atmosphere in dry CH_2Cl_2 (10 mL) and treated with DIEA (204 µL, 1.19 mmol), *N*-HBTU (141 mg, 0.371 mmol) and 5-azidopentanoic acid (47 mg, 0.327 mmol). The reaction mixture was stirred for 2 hours under argon atmosphere. Then, it was washed with aqueous solutions of HCl (5%, 3 x 5 mL) and $NaHCO_3$ (sat., 3 x 5 mL). The organic phase was dried over anhydrous $MgSO_4$, filtered and concentrated under vacuum. The resulting residue was purified by flash chromatography (1-2% MeOH/CH_2Cl_2) and the product was obtained as an orange solid [89 mg, 86%, R_f = 0.77 (10% MeOH/CH_2Cl_2)]. **^{1}H NMR** ($CDCl_3$, 300 MHz, δ): 8.40 (d, *J* = 8.5 Hz, 1H, H_{Ar}), 7.80 (br, 1H, NH), 6.41 (br, 1H, NH), 6.15 (d, *J* = 8.5 Hz, 1H, H_{Ar}), 3.70 (m, 2H, CH_2), 3.29 (t, *J* = 6.4 Hz, 2H, CH_2), 2.34 (t, *J* = 7.1 Hz, 2H, CH_2), 1.76 (m, 2H, CH_2), 1.63 (m, 2H, CH_2), 1.30 (m, 2H, CH_2). **^{13}C NMR** ($CDCl_3$, 75 MHz, δ): 175.3 (CO), 144.7 (C), 144.4 (C), 144.3 (C), 144.0 (C), 136.7 (CH), 98.8 (CH), 51.2 (CH_2), 38.8 (CH_2), 36.0 (CH_2), 35.8 (CH_2), 28.3 (CH_2), 22.7 (CH_2). **ESI-MS** *m/z* (%): 387.1 (32) $[M+K]^+$, 372.1 (100) $[M+Na]^+$, 349.1 (81) $[M+H]^+$. **HRMS (ESI)** *m/z*: $[M+H]^+$ calculated for $C_{13}H_{17}N_8O_4$: 349.1367, found 349.1355.

2.3. Solid-phase peptide synthesis

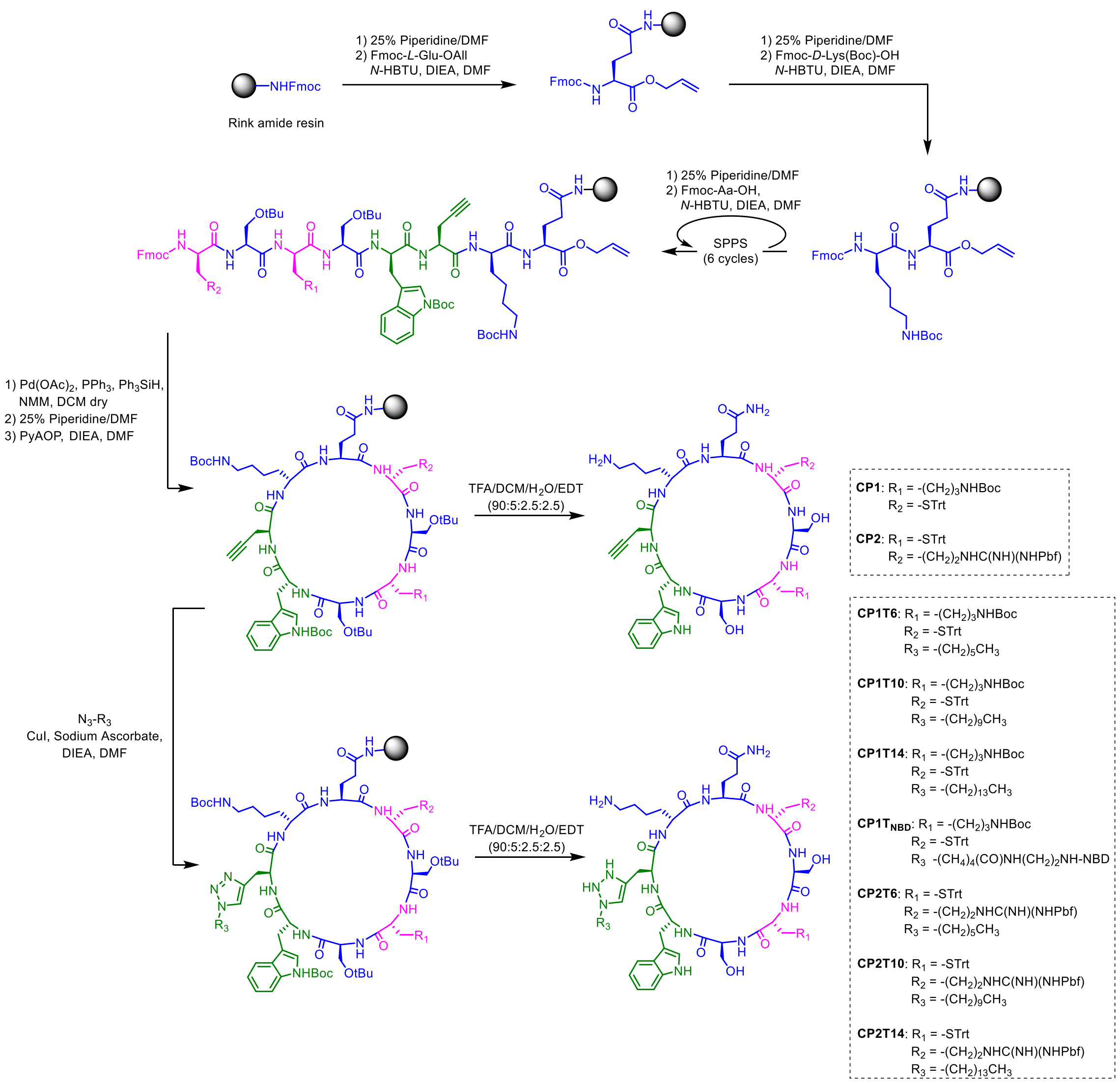

Figure S15. Synthetic strategy to obtain the cyclic peptide scaffolds.

General protocol of solid-phase synthesis: The synthesis of CPs was performed on a Rink Amide resin (100-200 mesh; 0.64 mmol·g^{-1}) using manual solid-phase synthesis according to the Fmoc-based strategy.[4] Briefly, Fmoc-Rink Amide resin (400 mg, 256 µmol) was swelled in DCM (3 mL) for 30 min in a peptide synthesis vessel and washed with DMF (6 x 4 mL) and DCM (6 x 4 mL). Fmoc group was removed by treating the resin with a solution of piperidine in DMF (25%, 4 mL) for 20 min. After DMF washings (3 x 4 mL), the coupling of the next amino acid was carried out using a solution of Fmoc-protected amino acid (3 equiv.), *N*-HBTU (3 equiv.), DIEA (6 equiv.) in DMF (4 mL) under orbital shaking for 40 min at room temperature. Once the reaction was completed, the resin was washed with DMF (3 x 4 mL) and DCM (3 x 4 mL). Cycles of deprotection and coupling were repeated until the desired linear peptide was achieved. Deprotection of the allyl protecting group was performed by adding to the resin a

degassed mixture of $Pd(OAc)_2$ (0.25 equiv.), PPh_3 (1.25 equiv.), *N*-methylmorpholine (6 equiv.) and phenylsilane (6 equiv.) in dry DCM (4 mL). The reaction was shaken for 2 hours at room temperature. Then, the resin was filtered and washed with DCM (3 x 4 mL), DMF (3 x 4 mL), sodium diethyldithiocarbamate in DMF (0.5%, 2 x 4 mL, 20 min), DMF (2 x 4 mL), DIEA in DMF (10%, 2 x 4 mL) and DMF (3 x 4 mL). To remove the *N*-terminal Fmoc group, the resin was treated with a solution of piperidine in DMF (25%, 4 mL, 30 min) and, then, washed with DMF (6 x 4 mL), DIEA in DMF (5%, 3 x 4 mL), LiCl in DMF (0.8 M, 3 x 4 mL) and DMF (3 x 4 mL). Cyclization was carried out by treating the resin with a solution of PyAOP (4 equiv.) and DIEA (6 equiv.) in DMF (4 mL) for 2 hours under orbital shaking. After filtration, the resin was washed with DMF (3 x 4 mL) and DCM (3 x 4 mL).

Click chemistry reaction: The functionalization with an alkyl chain was carried out when necessary. For this purpose, azide derivatives were prepared previously. A solution of the corresponding bromoalkane (6 equiv.) and NaN_3 (6 equiv.) in DMF (1 mL) was stirred for 24 hours at 80°C. The resulting azide derivative was used in the following reaction without further purification. The compound **NBD-N_3** was synthesized as previously described.

Then, the resulting azido derivative (6 equiv.), DMF (0.2 mL), piperidine (0.3 mL), CuI (5 equiv.), sodium ascorbate (5 equiv.) and DIEA (10 equiv.) were added to a vial containing the resin with the CP and the mixture was purged with argon for 5 min. The reaction mixture was shaken at room temperature for 48 hours under orbital shaking. After filtration, the resin was washed with DCM (3 x 4 mL), DMF (3 x 4 mL), piperidine in DMF (25%, 2 x 4 mL, 15 min), DMF (3 x 4 mL), MilliQ H_2O (2 x 4 mL, 10 min), DMF (3 x 4 mL) and DCM (3 x 4 mL).

Cleavage and purification: The CPs were cleaved from the resin and side-chain deprotected by shaking with a freshly prepared solution of TFA/DCM/H_2O/EDT (90:5:2.5:2.5, 1 mL per 100 mg resin) for 2 hours. The mixture was collected by filtration, concentrated with an argon steam and added dropwise to cold Et_2O (10 mL of Et_2O per 100 mg of resin). The precipitate was centrifuged (4000 rpm, 5 min) and the supernatant was discarded. After washings with diethyl ether (x3), the residue was dried under vacuum and solubilized in MilliQ-H_2O with 0.1% TFA. This solution was purified by semipreparative RP-HPLC using a Phenomenex Luna C18 (2) 100Å column. The proper fractions were freeze-dried to provide the desired CP. The pure product was stored at -20°C until further use.

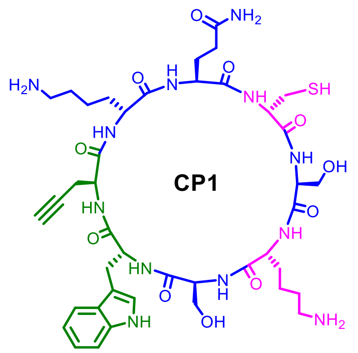

CP1: The peptide was synthesized according to the general protocol for solid-phase synthesis (400 mg of resin, 0.64 mmol·g^{-1}). Then, it was cleaved from the resin and purified by semipreparative RP-HPLC using a Phenomenex Luna C18 (2) 100Å column. [gradient: H_2O (0.1% TFA)/MeCN (0.1% TFA), 100:0 → 100:0 (5 min); 100:0 → 60:40 (40 min), R_t = 31 min]. The proper fractions were freeze-dried to provide **CP1** as a white solid with an overall yield of 14% (34 mg). **^{1}H NMR** (500 MHz, D_2O/H_2O): 10.18 (s, 1H, NH_{Indole}), 8.60 (d, J = 7.1 Hz, 1H, NH_{Gln}), 8.46 (d, J = 6.6 Hz, 1H, NH_{Ser}), 8.41 (d, J = 7.0 Hz, 1H, NH_{Cys}), 8.33 (d, J = 7.6 Hz, 1H, NH_{Lys}), 8.28 (d, J = 6.6 Hz, 1H, NH_{Trp}), 8.20 (d, J = 8.0 Hz, 1H, NH_{Prg}), 8.18 (d, J = 8.2 Hz, 1H, NH_{Lys}), 8.07 (d, J = 7.8 Hz, 1H, NH_{Ser}), 7.63 (d, J = 8.0 Hz, 1H, H_{Ar}), 7.58-7.42 (m, 5H, H_{Ar} and 2 $NH_{2\text{-}Lys}$), 7.25 (m, 1H, H_{Ar}), 7.23 (s, 1H, CH_{Indole}), 7.18 (m, 1H, H_{Ar}), 6.80 (br, 2H, $NH_{2\text{-}Gln}$), 4.72-4.24 (8 αCH), 3.89 (m, 2H, $CH_{2\text{-}Ser}$), 3.73 (m, 2H, $CH_{2\text{-}Ser}$), 3.30 (m, 2H, $CH_{2\text{-}Trp}$), 2.96 (m, 6H, 2 $CH_{2\text{-}Lys}$ and $CH_{2\text{-}Gln}$), 2.41 (m, 2H, $CH_{2\text{-}Cys}$), 2.30 (t, J = 8.0 Hz, 1H, CH_{Prg}), 2.18-1.88 (m, 2H, $CH_{2\text{-}Prg}$), 1.88-1.55 (m, 10 H, 4 $CH_{2\text{-}Lys}$ and $CH_{2\text{-}Gln}$), 1.36 (m, 4H, 2 $CH_{2\text{-}Lys}$). **ESI-MS** *m/z* (%): 943.5 (31) $[M+H]^+$, 472.4 (100) $[M+2H]^{2+}$. **HRMS (ESI)** *m/z*: $[M+H]^+$ calculated for $C_{42}H_{63}N_{12}O_{11}S$: 943.4454, found 943.4450. **FTIR (neat)**: ν = 3276.2 (amide A), 1666.9 (amide I_b), 1623.5 (amide I_a), 1534.7 (amide II) cm^{-1}.

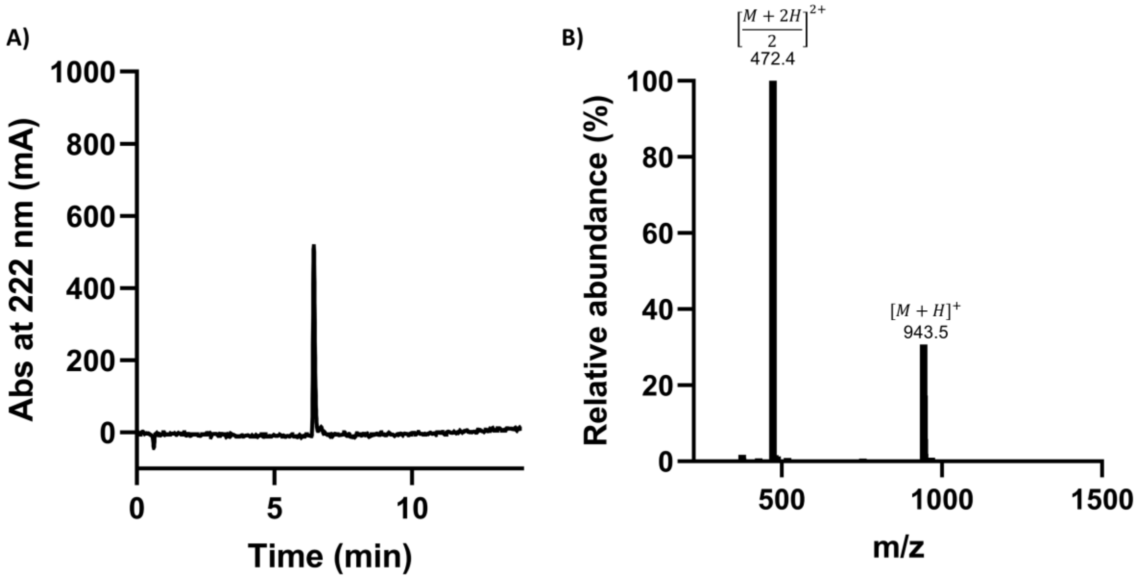

Figure S16. (A) **RP-uHPLC** of **CP1:** [Agilent SB-C18 column, H_2O (0.1% TFA)/MeCN (0.1% TFA), 100:0 →100:0 (2 min) and 100:0 → 50:50 (12 min)] R_t = 6.5 min. (B) ESI-MS of the main peak.

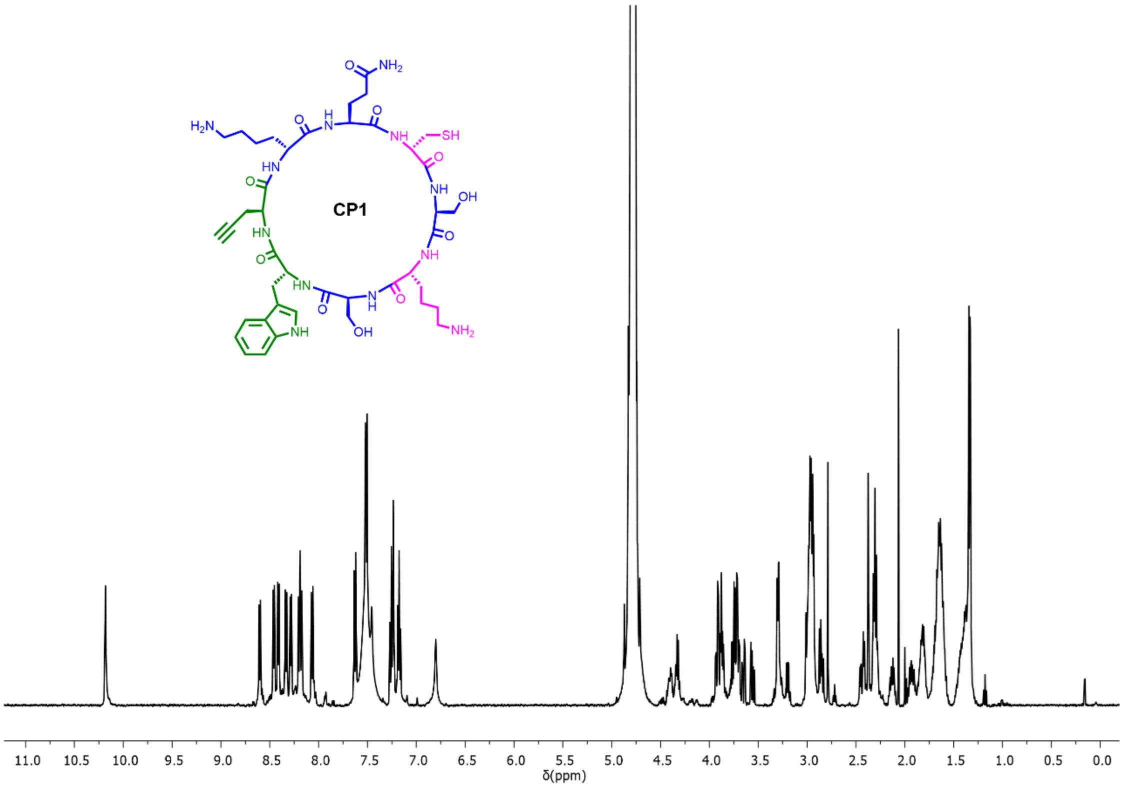

Figure S17. ^{1}H NMR of **CP1** at 5.1 mM (500 MHz, D_2O/H_2O, 298 K)

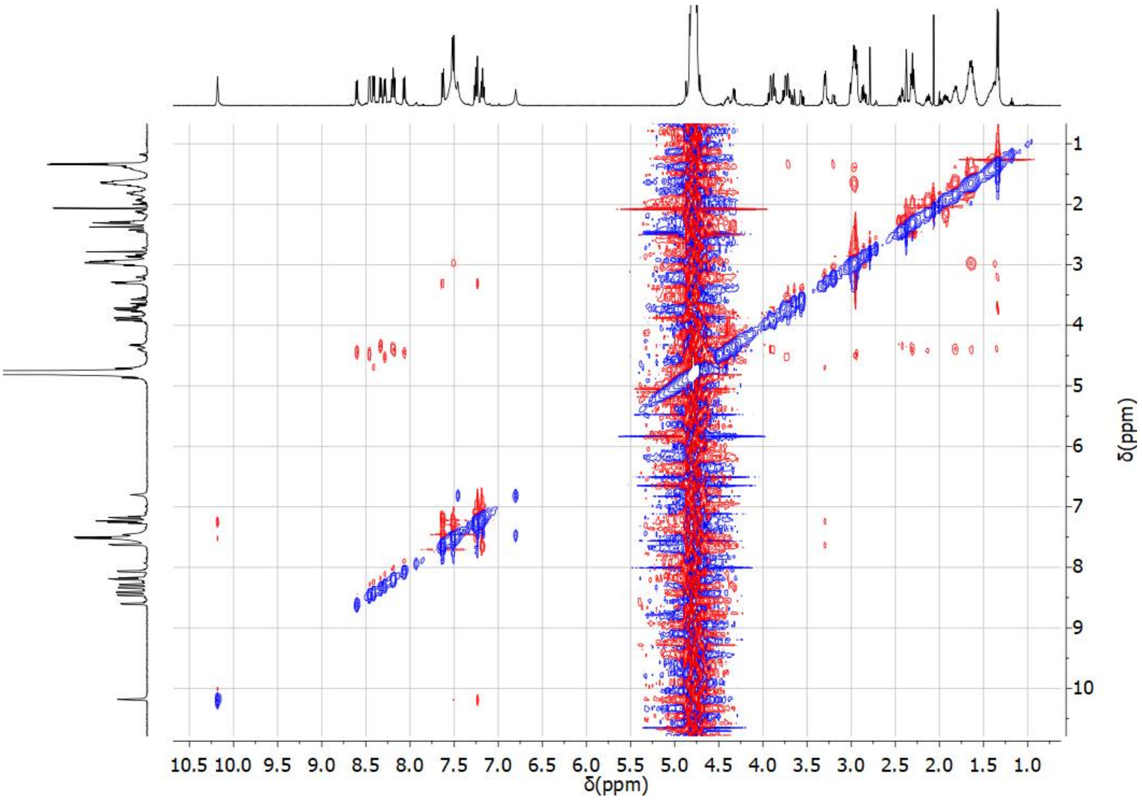

Figure S18. COSY of **CP1** at 5.1 mM (500 MHz, D_2O/H_2O, 298 K).

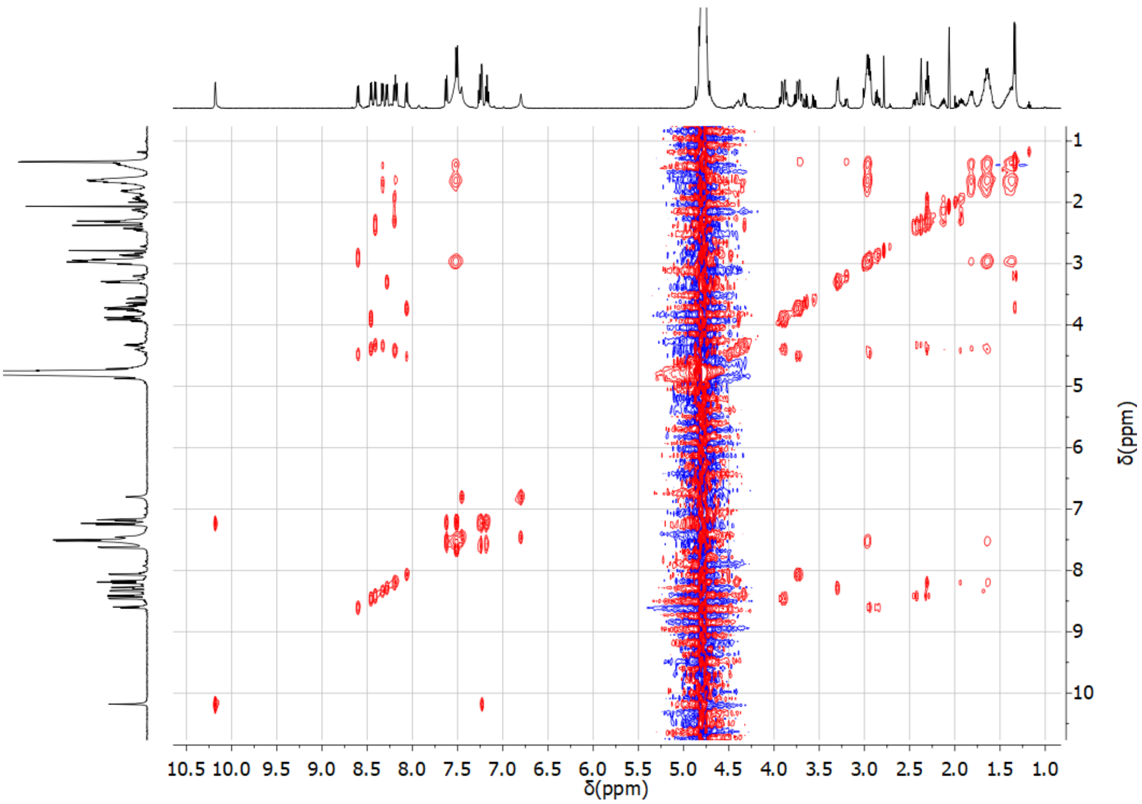

Figure S19. TOCSY of **CP1** at 5.1 mM (500 MHz, D_2O/H_2O, 298 K).

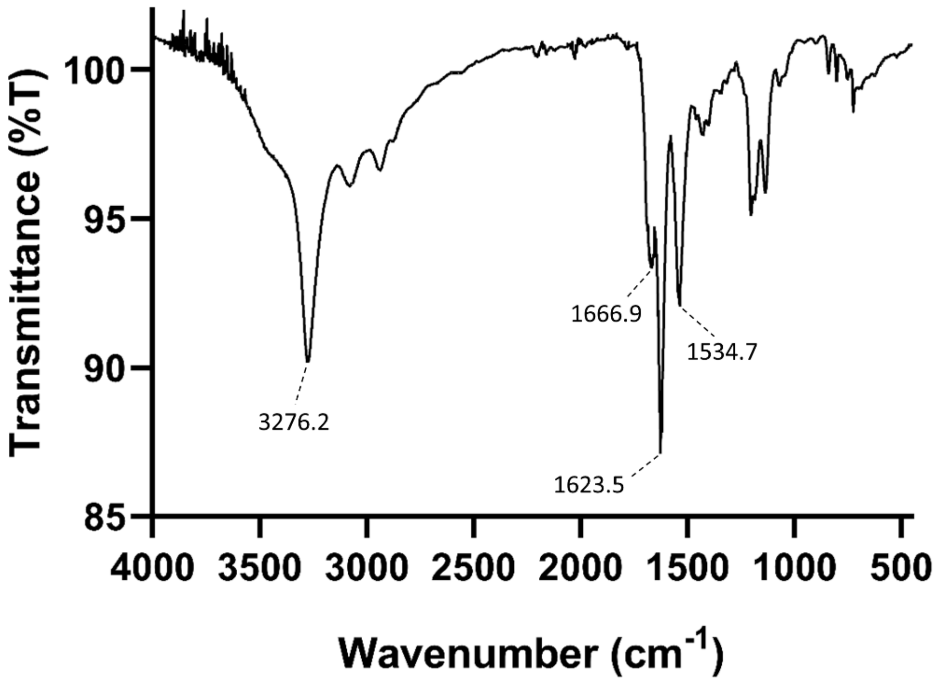

Figure S20. FT-IR spectrum of **CP1** (solid, 298 K).

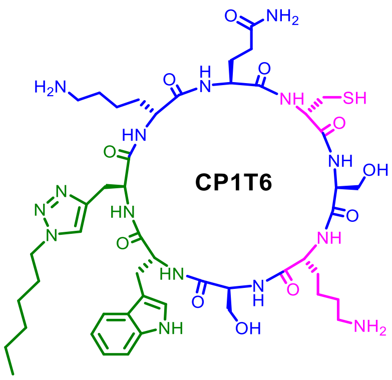

CP1T6: The peptide was synthesized according to the general protocol for solid-phase synthesis (150 mg of resin, 0.64 mmol·g^{-1}). Then, the click chemistry reaction was performed and the peptide was cleaved and purified by semipreparative RP-HPLC using a Phenomenex Luna C18 (2) 100Å column. [gradient: H_2O (0.1% TFA)/MeCN (0.1% TFA), 90:10 → 90:10 (2 min); 40:60 → 40:60 (40 min), R_t = 26.5 min]. The proper fractions were freeze-dried to provide **CP1T6** as a white solid with an overall yield of 4% (4.4 mg). **ESI-MS** *m/z* (%): 1070.5 (12) $[M+H]^+$, 535.9 (100) $[M+2H]^{2+}$, 357.6 (5) $[M+3H]^{3+}$. **HRMS (ESI)** *m/z*: $[M+H]^+$ calculated for $C_{48}H_{76}N_{15}O_{11}S$: 1070.5569, found 1070.5495. **FTIR (neat)**: ν = 3274.2 (amide A), 1662.9 (amide I_b), 1629.4 (amide I_a), 1534.7 (amide II) cm^{-1}.

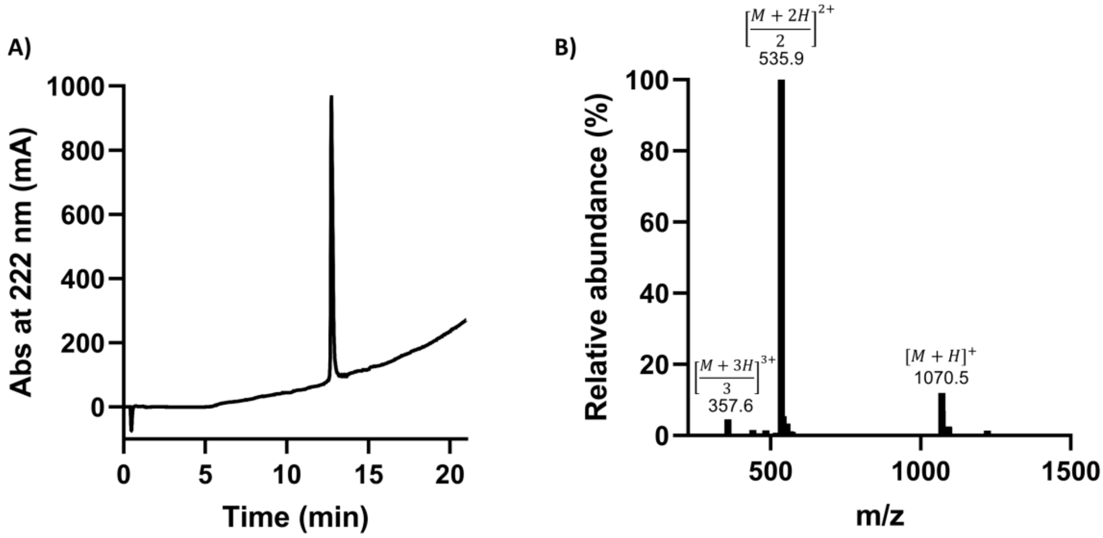

Figure S21. (A) **RP-uHPLC** of **CP1T6:** [Agilent SB-C18 column, H_2O (0.1% TFA)/MeCN (0.1% TFA), 100:0 → 100:2 (2 min) and 100:0 → 25:75 (19 min)] R_t = 12.8 min. (B) ESI-MS of the main peak.

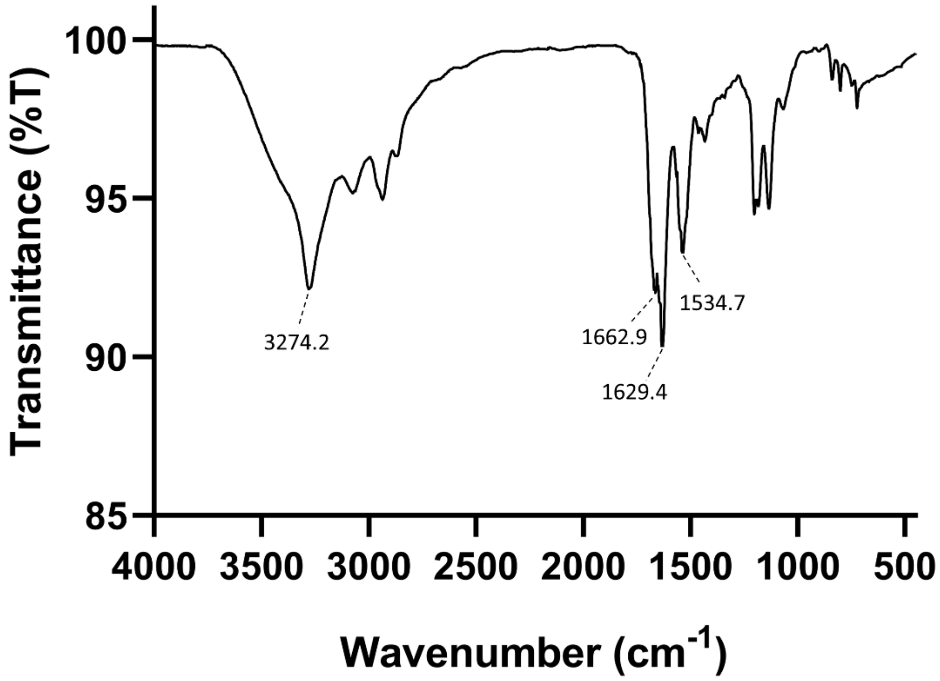

Figure S22. FT-IR spectrum of **CP1T6** (solid, 298 K).

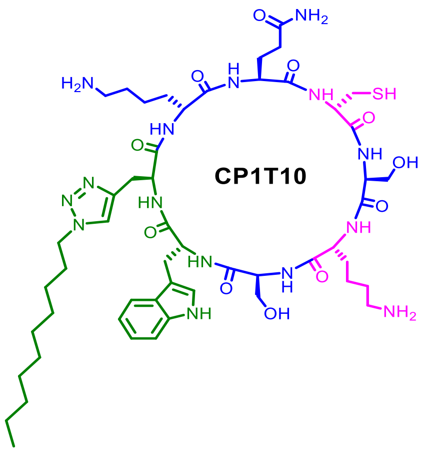

CP1T10: The peptide was synthesized according to the general protocol for solid-phase synthesis (150 mg of resin, 0.64 mmol·g^{-1}). Then, the click chemistry reaction was performed and the peptide was cleaved and purified by semipreparative RP-HPLC using a Phenomenex Luna C18 (2) 100Å column. [gradient: H_2O (0.1% TFA)/MeCN (0.1% TFA), 85:15 → 85:15 (5 min); 85:15 → 25:75 (40 min), R_t = 28.5 min]. The proper fractions were freeze-dried to provide **CP1T10** as a white solid with an overall yield of 20% (22.1 mg). **ESI-MS** *m/z* (%): 1126.7 (11) $[M+H]^+$, 564.0 (100) $[M+2H]^{2+}$, 376.3 (3) $[M+3H]^{3+}$. **HRMS (ESI)** *m/z*: $[M+2H]^{2+}$ calculated for $C_{52}H_{85}N_{15}O_{11}S$: 563.8131, found 563.8139. **FTIR (neat)**: ν = 3276.2 (amide A), 1670.8 (amide I_b), 1627.4 (amide I_a), 1540.6 (amide II) cm^{-1}.

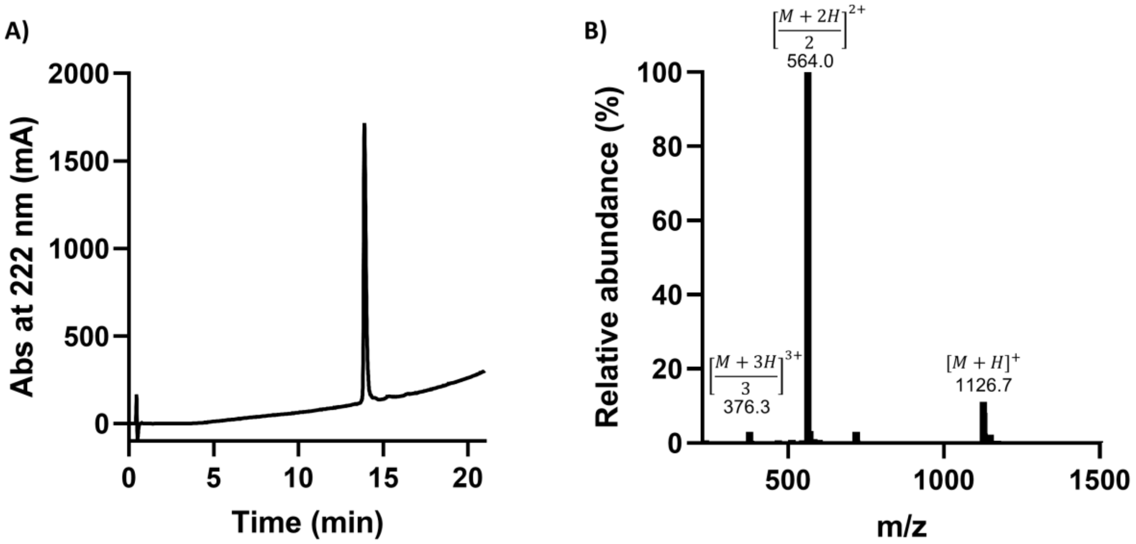

Figure S23. (A) **RP-uHPLC** of **CP1T10:** [Agilent SB-C18 column, H_2O (0.1% TFA)/MeCN (0.1% TFA), 100:0 → 100:2 (2 min) and 100:0 → 25:75 (19 min)] R_t = 13.8 min. (B) ESI-MS of the main peak.

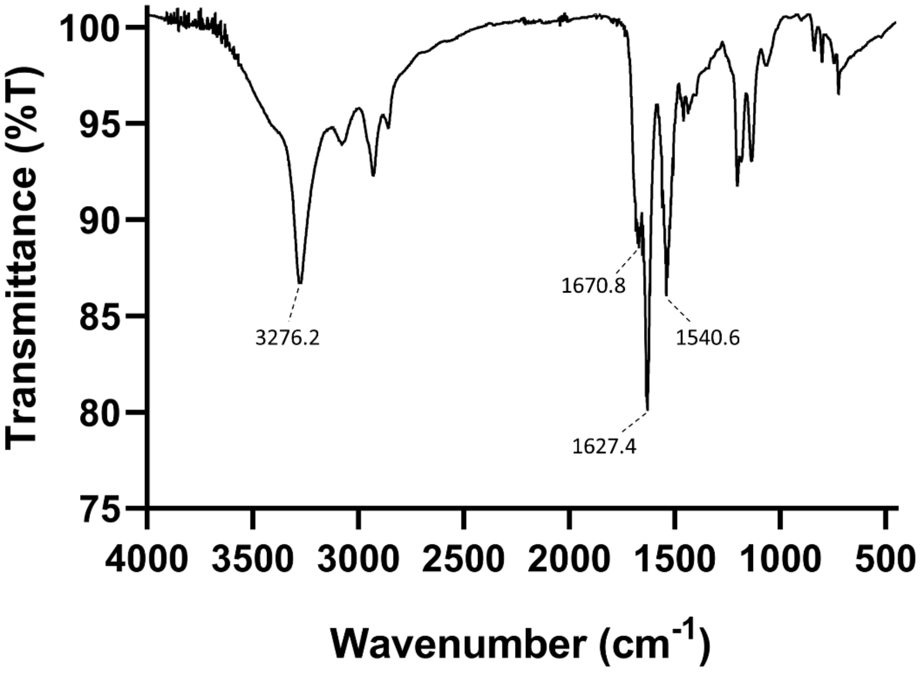

Figure S24. FT-IR spectrum of **CP1T10** (solid, 298 K).

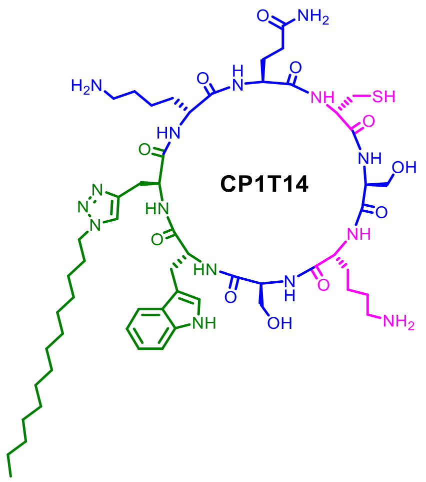

CP1T14: The peptide was synthesized according to the general protocol for solid-phase synthesis (150 mg of resin, 0.64 mmol·g^{-1}). Then, the click chemistry reaction was performed and the peptide was cleaved and purified by semipreparative RP-HPLC using a Phenomenex Luna C18 (2) 100Å column. [gradient: H_2O (0.1% TFA)/MeCN (0.1% TFA), 90:10 → 90:10 (5 min); 90:10 → 25:75 (40 min), R_t = 35.0 min]. The proper fractions were freeze-dried to provide **CP1T14** as a white solid with an overall yield of 5% (6.1 mg). **ESI-MS** *m/z* (%): 1182.7 (6) $[M+H]^+$, 591.9 (100) $[M+2H]^{2+}$, 394.9 (4) $[M+3H]^{3+}$. **HRMS (ESI)** *m/z*: $[M+H]^+$ calculated for $C_{56}H_{92}N_{15}O_{11}S$: 1182.6816, found 1182.6789. **FTIR (neat)**: ν = 3274.2 (amide A), 1670.8 (amide I_b), 1627.4 (amide I_a), 1538.7 (amide II) cm^{-1}.

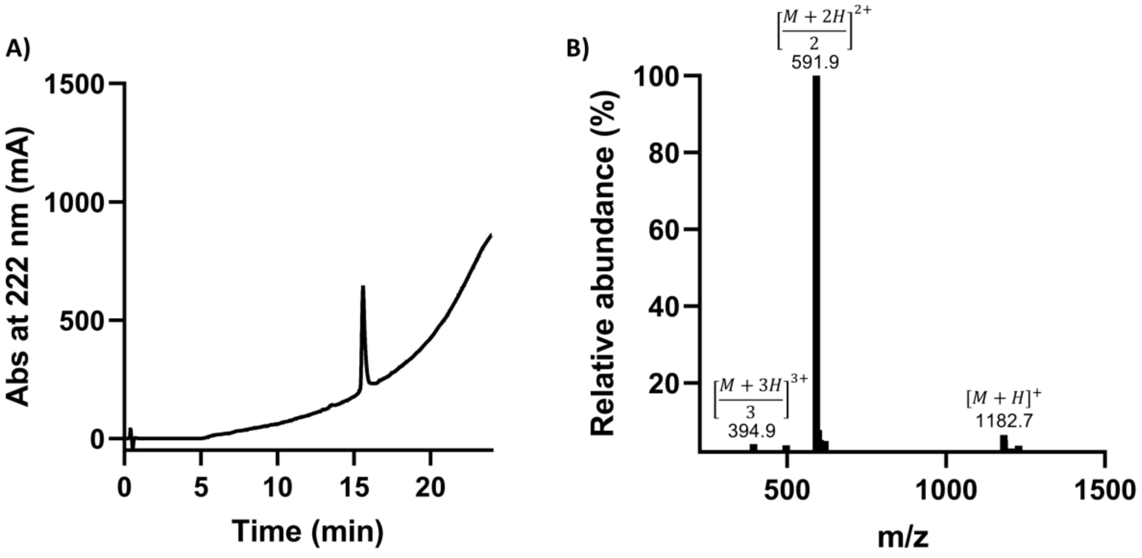

Figure S25. (A) **RP-uHPLC** of **CP1T14:** [Agilent SB-C18 column, H_2O (0.1% TFA)/MeCN (0.1% TFA), 100:0 → 100:2 (2 min) and 100:0 → 5:95 (19 min)] R_t = 15.6 min. (B) ESI-MS of the main peak.

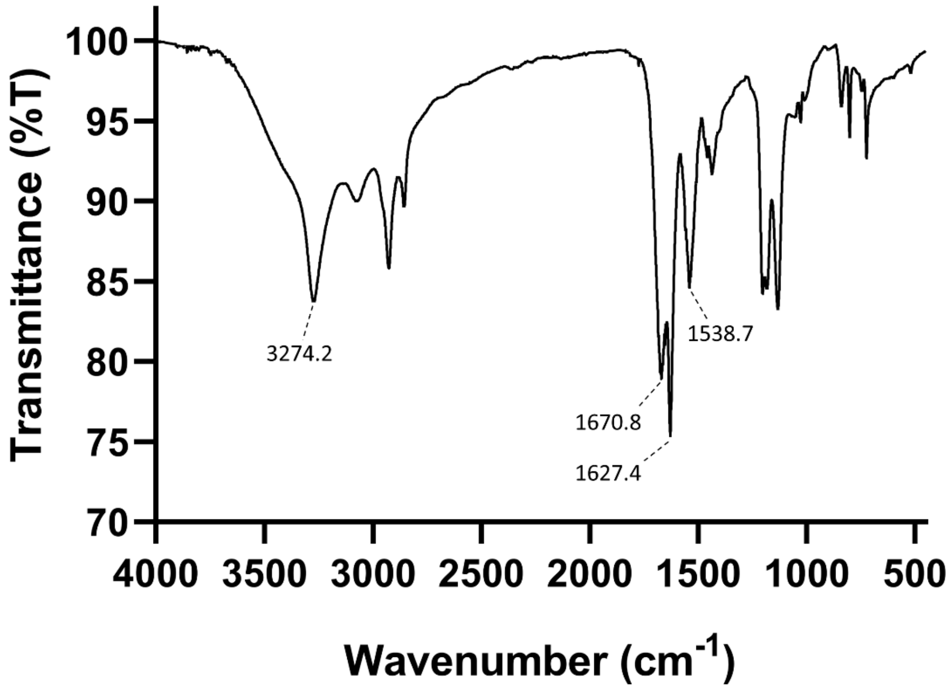

Figure S26. FT-IR spectrum of **CP1T14** (solid, 298 K).

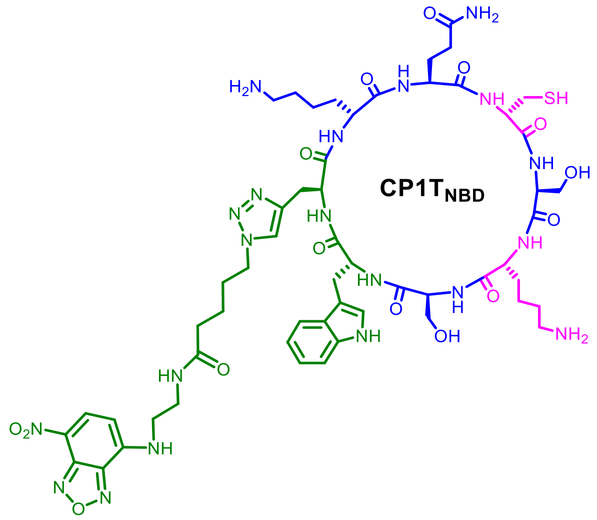

CP1T$_{NBD}$: The peptide was synthesized according to the general protocol for solid-phase synthesis (75 mg of resin, 0.64 mmol·g^{-1}). Then, the click chemistry reaction was performed and the peptide was cleaved and purified by semipreparative RP-HPLC using a Phenomenex Luna C18 (2) 100Å column. [gradient: H_2O (0.1% TFA)/MeCN (0.1% TFA), 100:0 → 100:0 (5 min); 100:0 → 50:50 (40 min), R_t= 25.0 min]. The proper fractions were freeze-dried to provide **CP1T$_{NBD}$** as an orange solid with an overall yield of 7% (4.3 mg). **ESI-MS** *m/z* (%): 1291.6 (5) $[M+H]^+$, 646.3 (100) $[M+2H]^{2+}$, 431.5 (7) $[M+3H]^{3+}$. **HRMS (ESI)** *m/z*: $[M+H]^+$ calculated for $C_{55}H_{79}N_{20}O_{15}S$: 1291.5749, found 1291.5741. **FTIR (neat)**: ν = 3272.3 (amide A), 1672.8 (amide I_b), 1623.5 (amide I_a), 1538.7 (amide II) cm^{-1}.

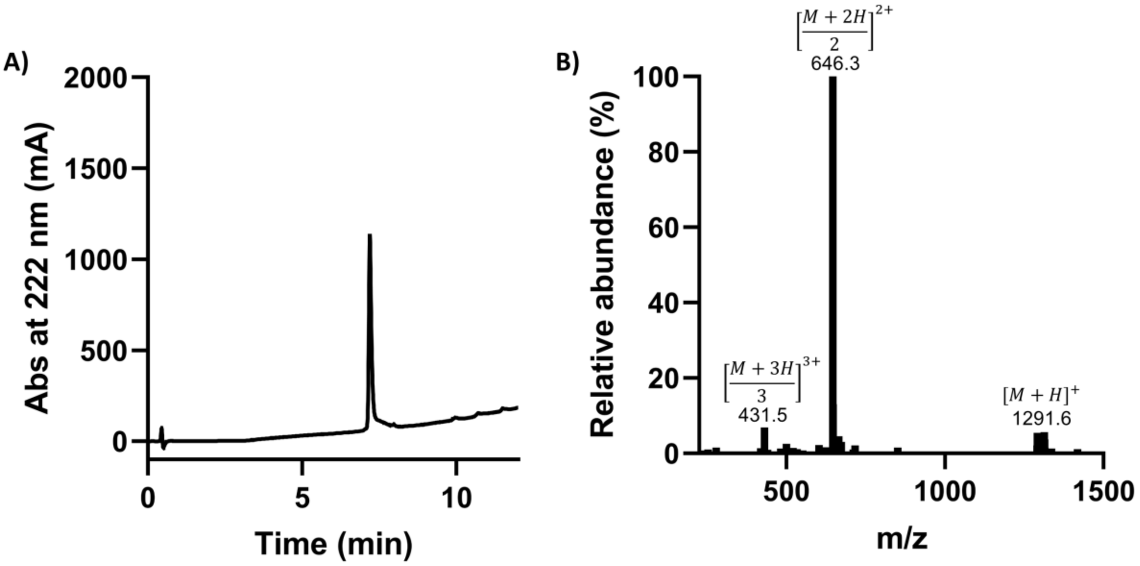

Figure S27. (A) **RP-uHPLC** of **CP1T$_{NBD}$:** [Agilent SB-C18 column, H_2O (0.1% TFA)/MeCN (0.1% TFA), 100:0 →100:0 (2 min) and 100:0 → 50:50 (12 min)], R_t = 7.2 min. (B) ESI-MS of the main peak.

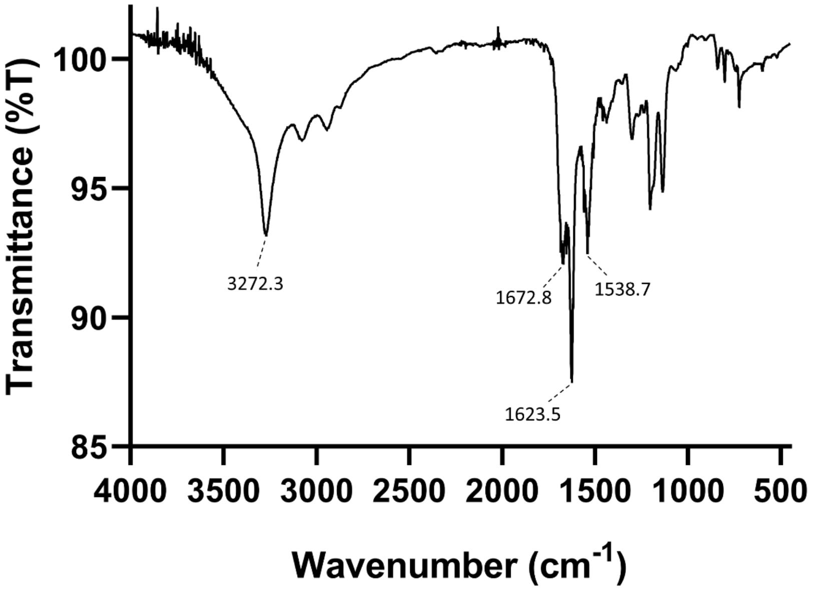

Figure S28. FT-IR spectrum of **CP1T$_{NBD}$** (solid, 298 K).

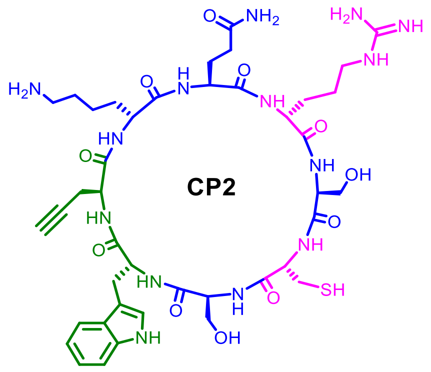

CP2: The peptide was synthesized according to the general protocol for solid-phase synthesis (400 mg of resin, 0.64 mmol·g^{-1}). Then, it was cleaved from the resin and purified by semipreparative RP-HPLC using a Phenomenex Luna C18 (2) 100Å column. [gradient: H_2O (0.1% TFA)/MeCN (0.1% TFA), 90:10 → 90:10 (5 min); 90:10 → 60:40 (40 min), R_t = 25.9 min]. The proper fractions were freeze-dried to provide **CP2** as a white solid with an overall yield of 3 % (8 mg). **^{1}H NMR** (500 MHz, D_2O/H_2O): 10.09 (s, 1H, NH_{Indole}), 8.39 (m, 2H, NH_{Gln} and NH_{Ser}), 8.35 (d, J = 7.4 Hz, 1H, NH_{Cys}), 8.26 (m, 3H, NH_{Prg}, NH_{Lys} and NH_{Trp}), 8.22 (d, J = 7.4 Hz, 1H, NH_{Arg}), 8.17 (d, J = 7.5 Hz, 1H, NH_{Ser}), 7.56 (d, J = 8.0 Hz, 1H, H_{Ar}), 7.44 (m, 3H, $H_{Ar,}$ $NH_{2\text{-}Lys}$), 7.20 (m, 1H, H_{Ar}), 7.17 (s, 1H, CH_{Indole}), 7.11 (m, 1H, H_{Ar}), 7.07-6.70 (m, 2H, $NH_{2\text{-}Gln}$), 4.46-4.17 (8 αCH), 3.83 (m, 2H, $CH_{2\text{-}Ser}$), 3.71 (m, 2H, $CH_{2\text{-}Ser}$), 3.21 (m, 4H, $CH_{2\text{-}Arg}$ and $CH_{2\text{-}Trp}$), 3.05 (m, 2H, $CH_{2\text{-}Gln}$), 2.92 (m, 2H, $CH_{2\text{-}Lys}$), 2.82 (m, 2H, $CH_{2\text{-}Cys}$), 2.34 (m, 1H, CH_{Prg}), 2.24 (m, 4H, $CH_{2\text{-}Arg}$ and $CH_{2\text{-}Prg}$), 2.09-1.81 (m, 2H, $CH_{2\text{-}Arg}$), 1.70-1.44 (m, 6H, 2 $CH_{2\text{-}Lys}$ and $CH_{2\text{-}Gln}$), 1.34 (m, 2H, $CH_{2\text{-}Lys}$). **ESI-MS** *m/z* (%): 971.5 (21) $[M+H]^+$, 486.3 (100) $[M+2H]^{2+}$. **HRMS (ESI)** *m/z*: $[M+2H]^{2+}$ calculated for $C_{42}H_{64}N_{14}O_{11}S$: 486.2294, found 486.2296. **FTIR (neat)**: ν = 3270.3 (amide A), 1664.9 (amide I_b), 1625.4 (amide I_a), 1536.7 (amide II) cm^{-1}.

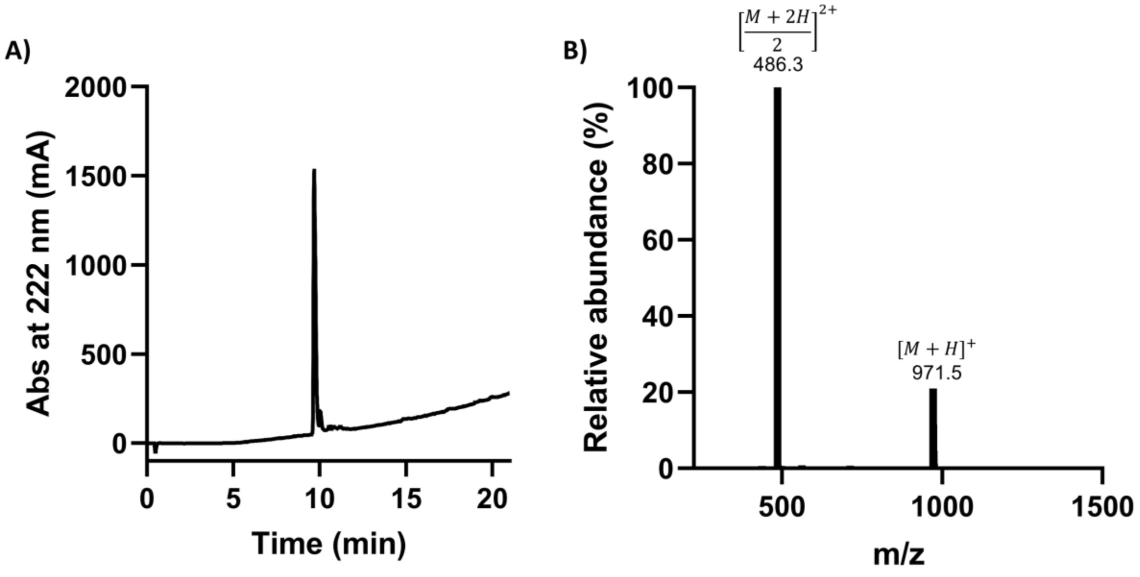

Figure S29. (A) **RP-uHPLC** of **CP2:** [Agilent SB-C18 column, H_2O (0.1% TFA)/MeCN (0.1% TFA), 100:0 → 100:2 (2 min) and 100:0 → 25:75 (19 min)], R_t = 9.6 min. (B) ESI-MS of the main peak.

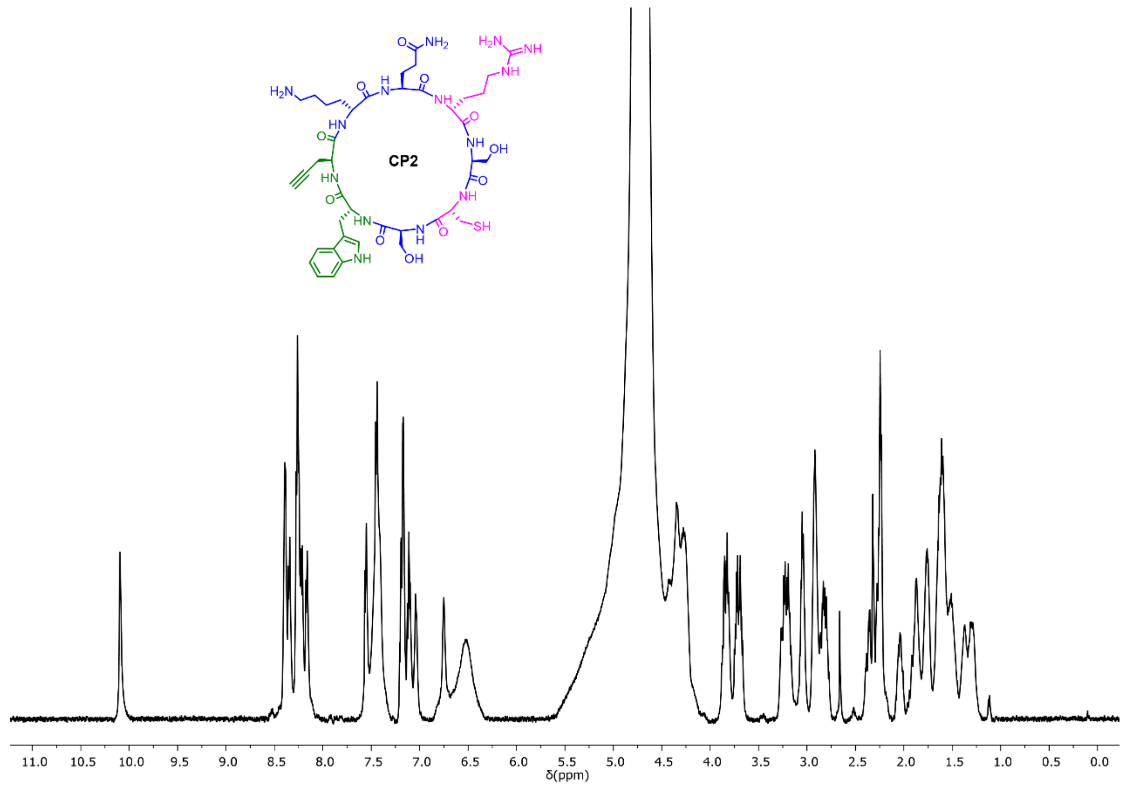

Figure S30. ^{1}H NMR of **CP2** at 5.5 mM (500 MHz, D_2O/H_2O, 298 K)

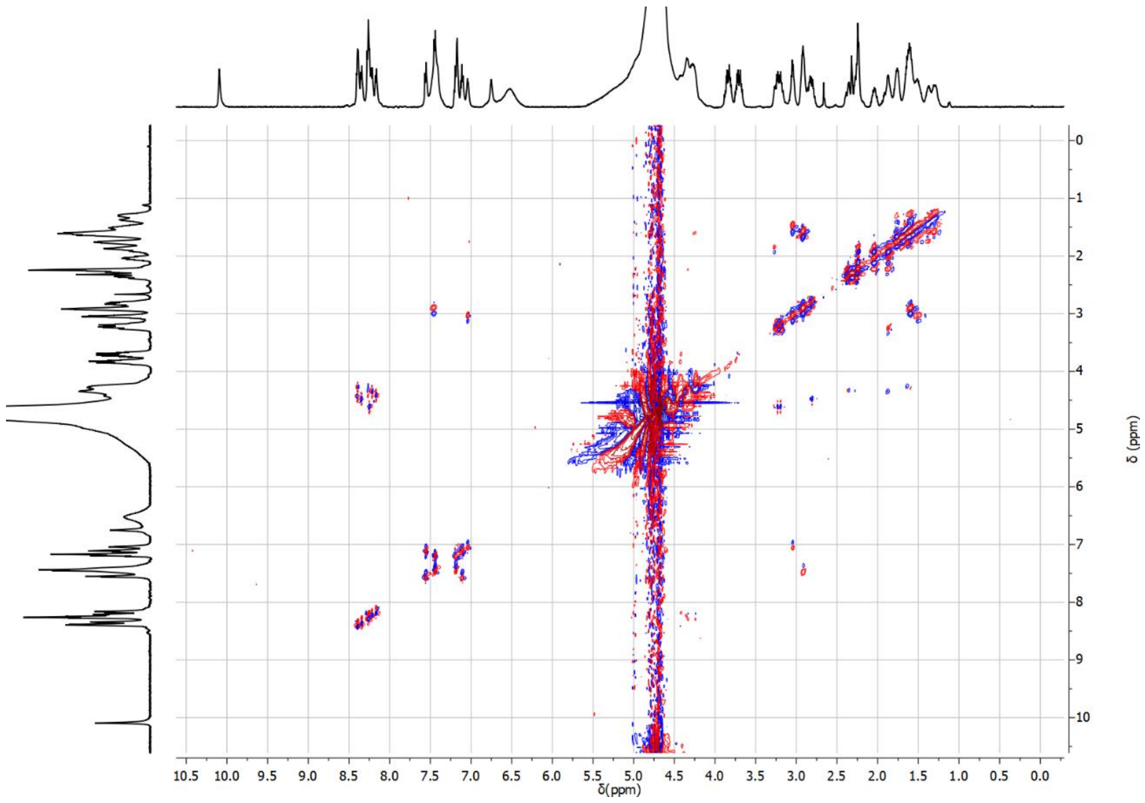

Figure S31. COSY of **CP2** at 5.5 mM (500 MHz, D_2O/H_2O, 298 K).

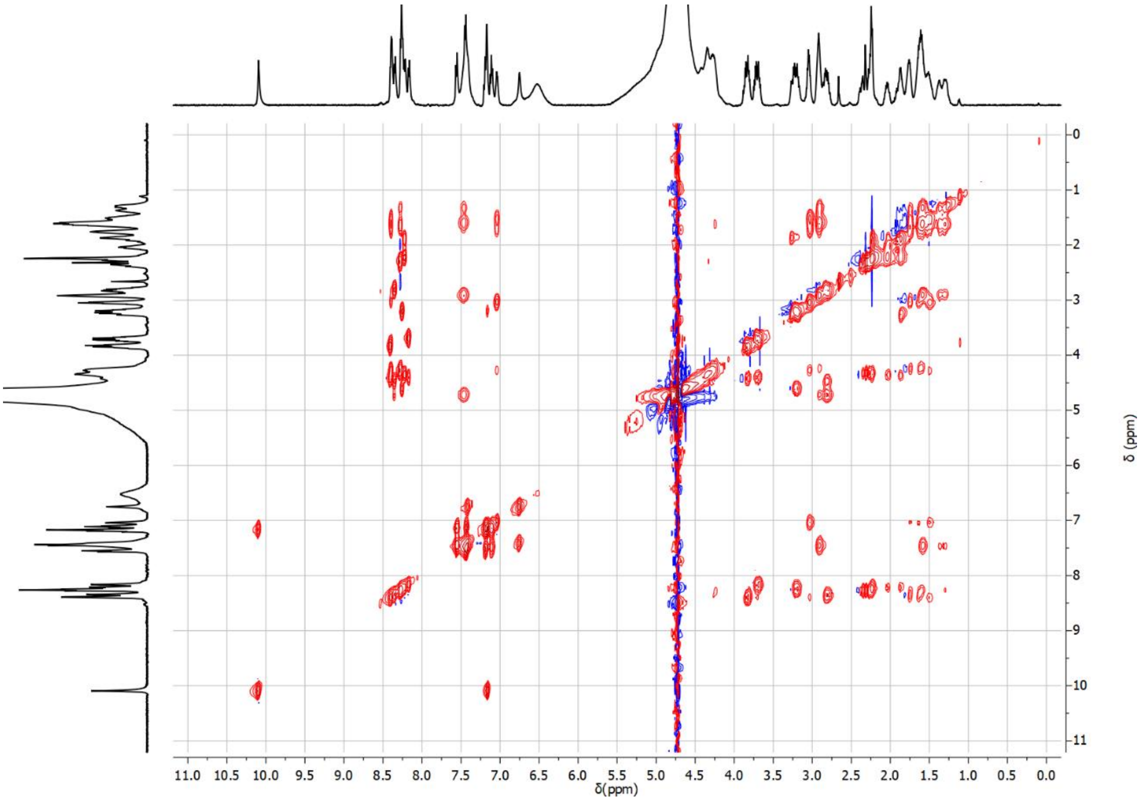

Figure S32. TOCSY of **CP2** at 5.5 mM (500 MHz, D_2O/H_2O, 298 K).

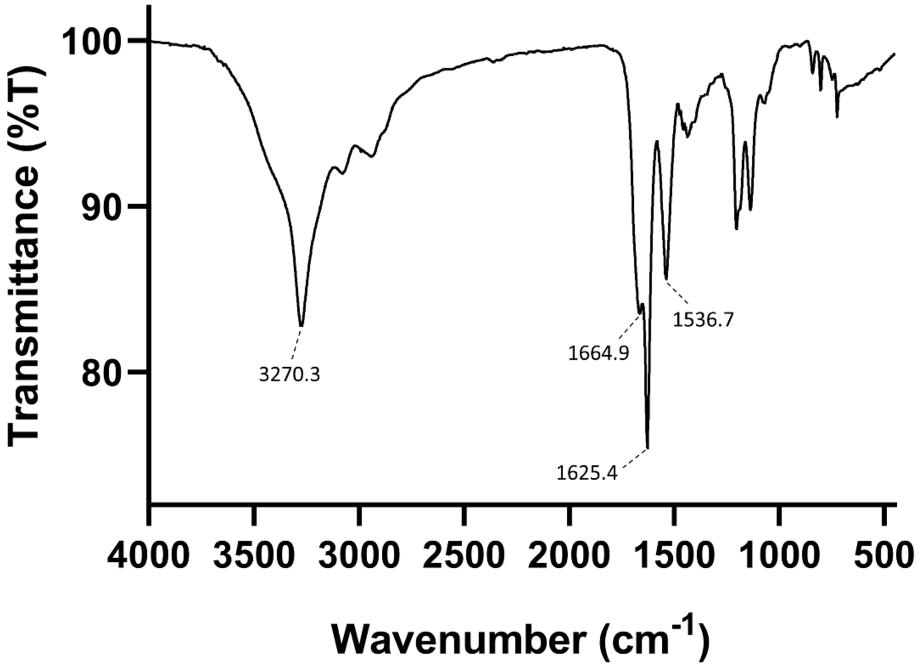

Figure S33. FT-IR spectrum of **CP2** (solid, 298 K).

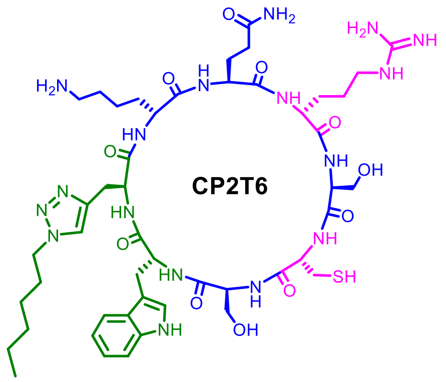

CP2T6: The peptide was synthesized according to the general protocol for solid-phase synthesis (188 mg of resin, 0.64 mmol·g^{-1}). Then, the click chemistry reaction was performed and the peptide was cleaved and purified by semipreparative RP-HPLC using a Phenomenex Luna C18 (2) 100Å column. [gradient: H_2O (0.1% TFA)/MeCN (0.1% TFA), 90:10 → 90:10 (5 min); 90:10 → 40:60 (40 min), R_t = 27.4 min]. The proper fractions were freeze-dried to provide **CP2T6** as a white solid with an overall yield of 4% (5.6 mg). **ESI-MS** *m/z* (%): 1098.6 (16) $[M+H]^+$, 549.9 (100) $[M+2H]^{2+}$, 366.9 (5) $[M+3H]^{3+}$. **HRMS (ESI)** *m/z*: $[M+2H]^{2+}$ calculated for $C_{48}H_{77}N_{17}O_{11}S$: 549.7849, found 549.7851. **FTIR (neat)**: ν = 3274.2 (amide A), 1660.9 (amide Ib), 1625.4 (amide Ia), 1538.7 (amide II) cm^{-1}.

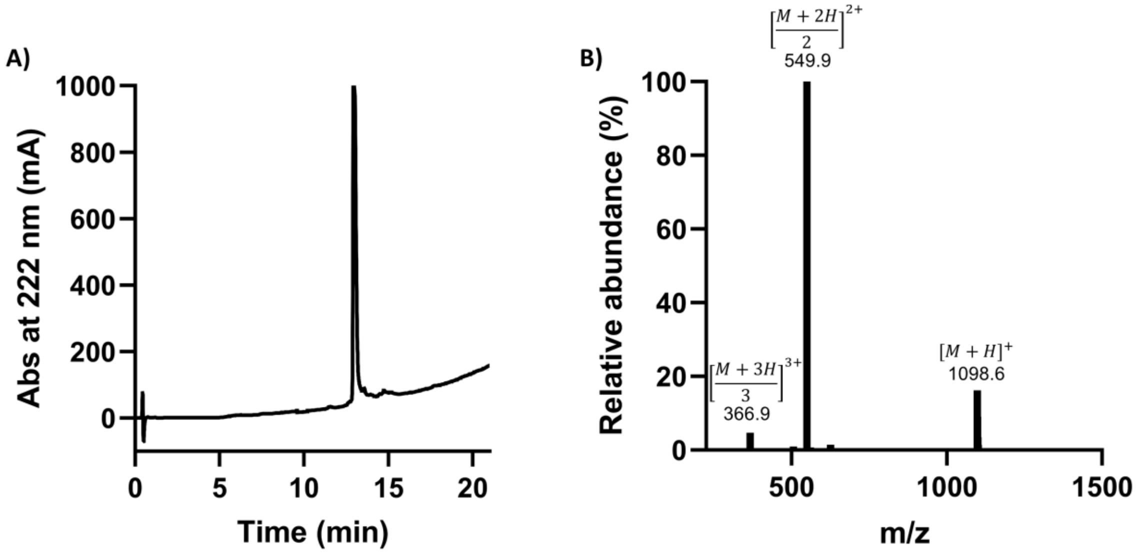

Figure S34. (A) **RP-uHPLC** of **CP2T6:** [Agilent SB-C18 column, H_2O (0.1% TFA)/MeCN (0.1% TFA), 100:0 → 100:2 (2 min) and 100:0 → 25:75 (19 min)], R_t = 12.3 min. (B) ESI-MS of the main peak.

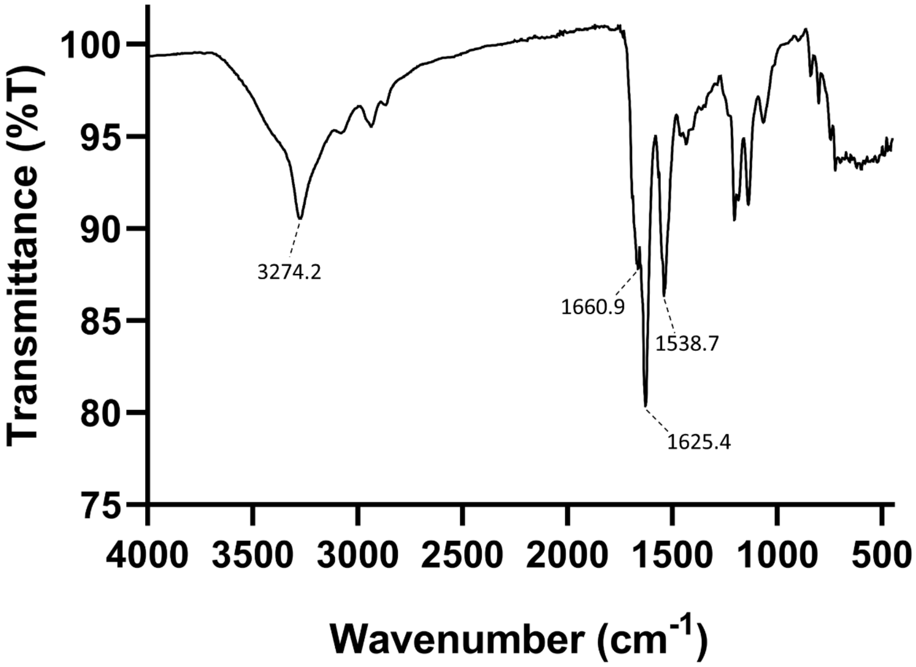

Figure S35. FT-IR spectrum of **CP2T6** (solid, 298 K).

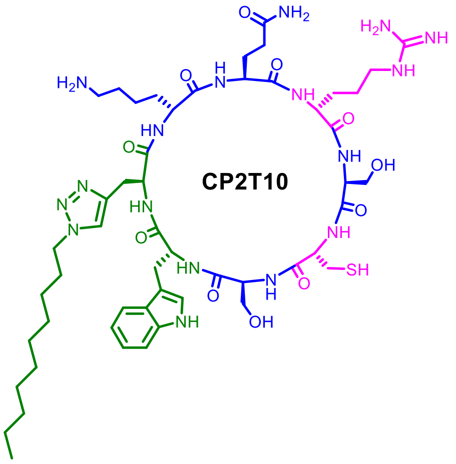

CP2T10: The peptide was synthesized according to the general protocol for solid-phase synthesis (100 mg of resin, 0.64 mmol·g^{-1}). Then, the click chemistry reaction was performed and the peptide was cleaved and purified by semipreparative RP-HPLC using a Phenomenex Luna C18 (2) 100Å column. [gradient: H_2O (0.1% TFA)/MeCN (0.1% TFA), 85:15 → 85:15 (5 min); 85:15 → 25:75 (40 min), R_t= 29.2 min]. The proper fractions were freeze-dried to provide **CP2T10** as a white solid with an overall yield of 12% (8.5 mg). **ESI-MS** *m/z* (%): 1154.6 (13) $[M+H]^+$, 578.0 (100) $[M+2H]^{2+}$, 385.8 (5) $[M+3H]^{3+}$. **HRMS (ESI)** *m/z*: $[M+2H]^{2+}$ calculated for $C_{52}H_{85}N_{17}O_{11}S$: 577.8162, found 577.8163. **FTIR (neat)**: ν = 3274.2 (amide A), 1668.8 (amide I_b), 1627.4 (amide I_a), 1538.7 (amide II) cm^{-1}.

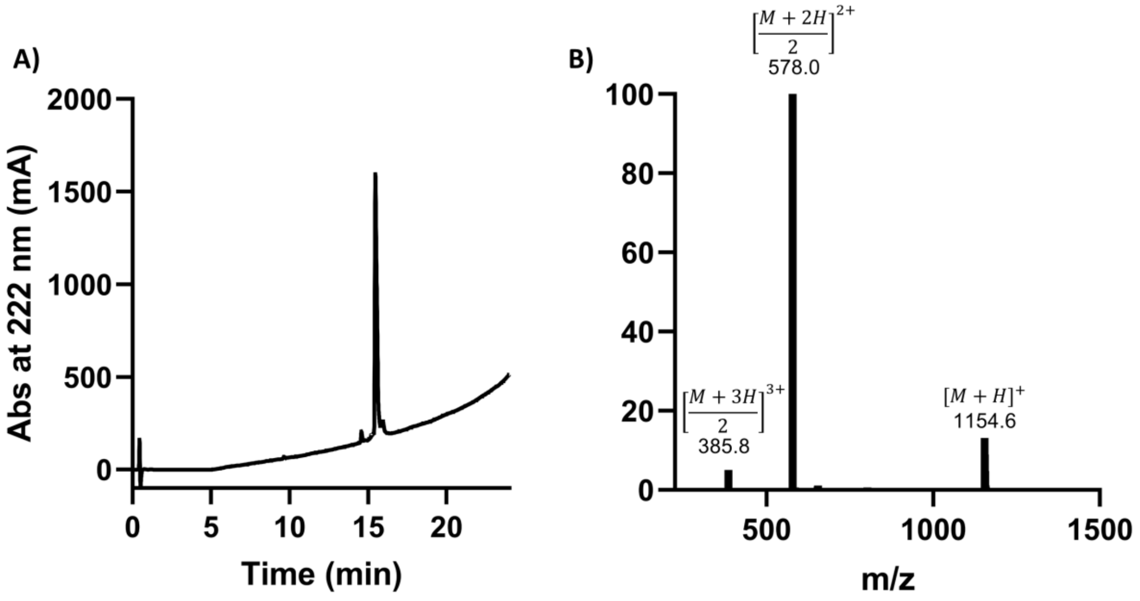

Figure S36. (A) **RP-uHPLC** of **CP2T10:** [Agilent SB-C18 column, H_2O (0.1% TFA)/MeCN (0.1% TFA), 100:0 → 100:2 (2 min) and 100:0 → 25:75 (19 min)], R_t = 15.4 min. (B) ESI-MS of the main peak.

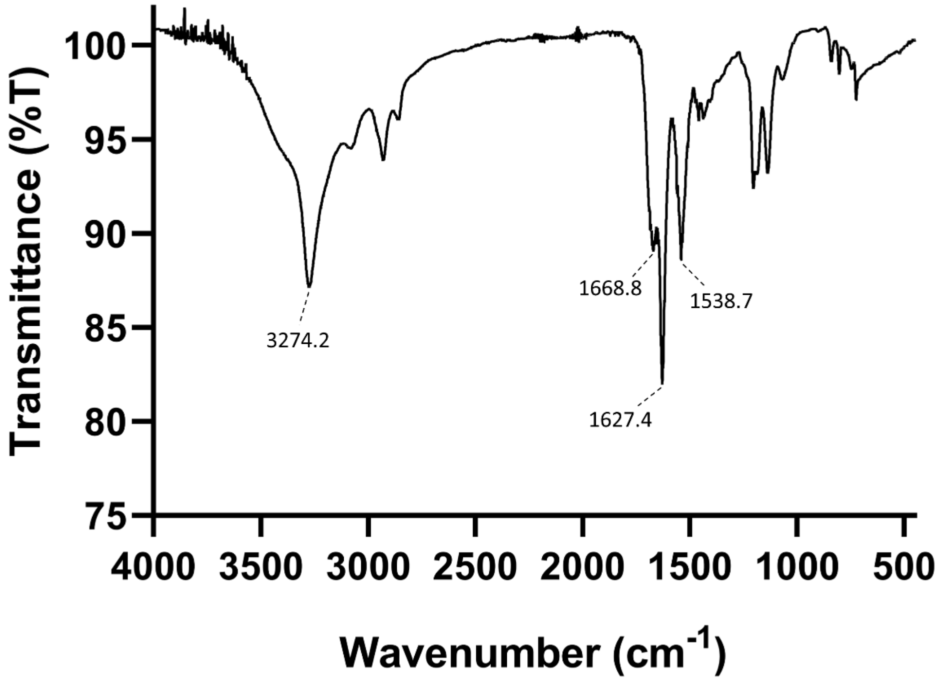

Figure S37. FT-IR spectrum of **CP2T10** (solid, 298 K).

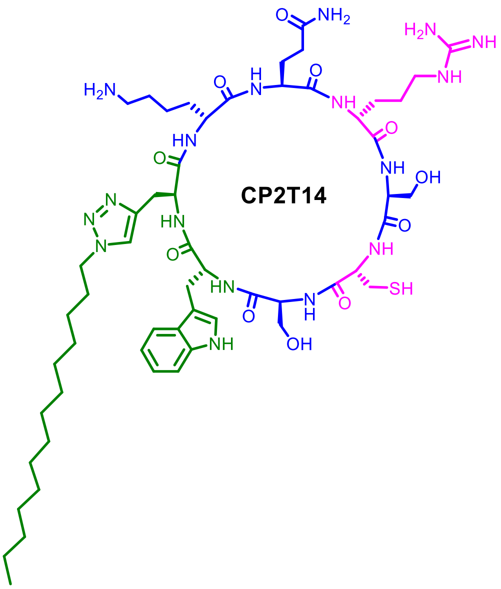

CP2T14: The peptide was synthesized according to the general protocol for solid-phase synthesis (145 mg of resin, 0.64 mmol·g^{-1}). Then, the click chemistry reaction was performed and the peptide was cleaved and purified by semipreparative RP-HPLC using a Phenomenex Luna C18 (2) 100Å column. [gradient: H_2O (0.1% TFA)/MeCN (0.1% TFA), 70:30 → 70:30 (5 min); 70:30 → 15:85 (40 min), R_t = 24.9 min]. The proper fractions were freeze-dried to provide **CP2T14** as a white solid with an overall yield of 2% (2.5 mg). **ESI-MS** *m/z* (%): 1210.7 (13) $[M+H]^+$, 605.6 (100) $[M+2H]^{2+}$, 404.2 (4) $[M+3H]^{3+}$. **HRMS (ESI)** *m/z*: $[M+H]^+$ calculated for $C_{56}H_{92}N_{17}O_{11}S$: 1210.6877, found 1210.6901. **FTIR (neat)**: ν = 3268.3 (amide A), 1668.8 (amide I_b), 1627.4 (amide I_a), 1538.7 (amide II) cm^{-1}.

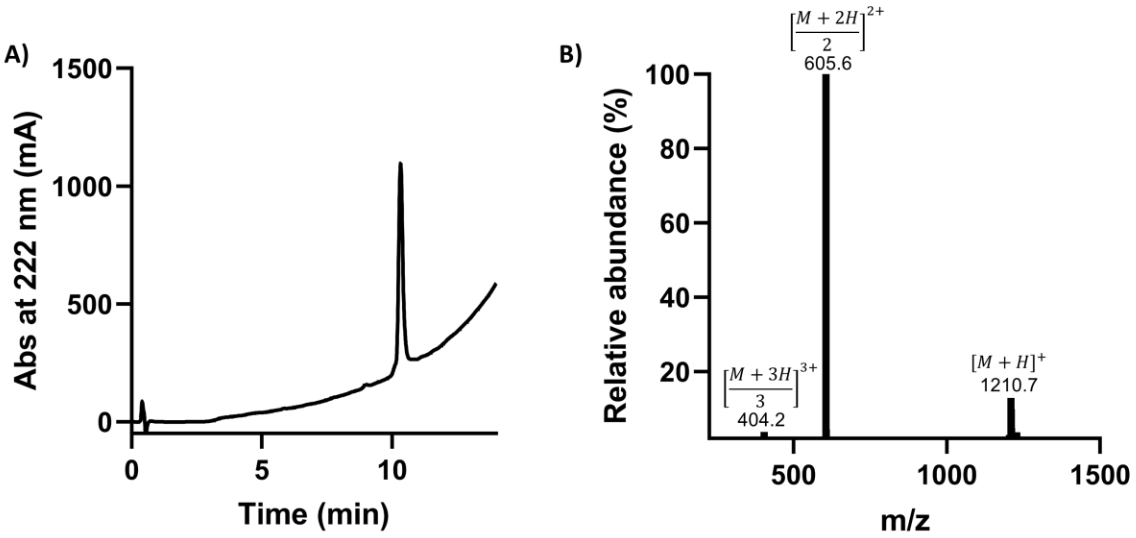

Figure S38. (A) **RP-uHPLC** of **CP2T14:** [Agilent SB-C18 column, H_2O (0.1% TFA)/MeCN (0.1% TFA), 100:0 → 100:2 (2 min) and 100:0 → 25:75 (12 min)], R_t = 10.3 min. (B) ESI-MS of the main peak.

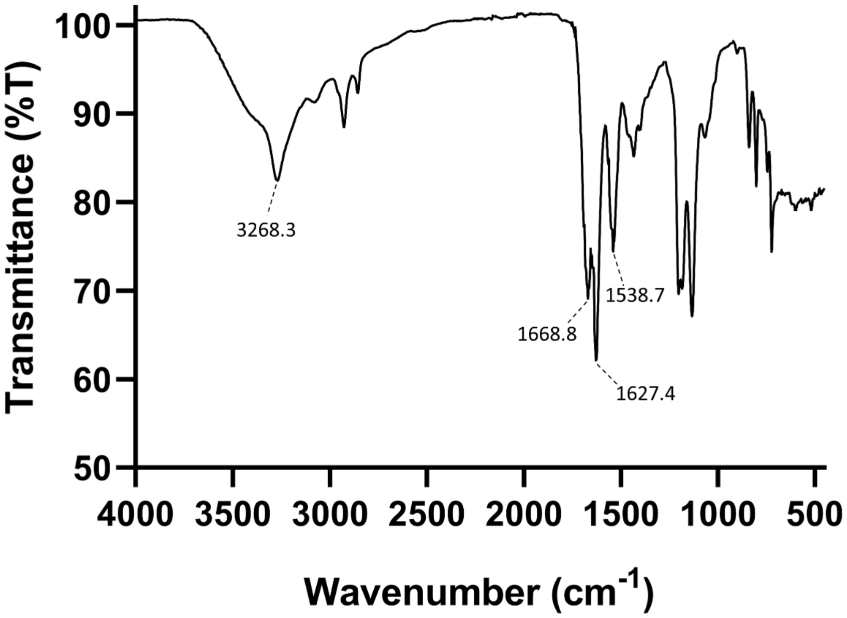

Figure S39. FT-IR spectrum of **CP2T14** (solid, 298 K).

2.4. Maleimide-thiol doxorubicin conjugation

General protocol:

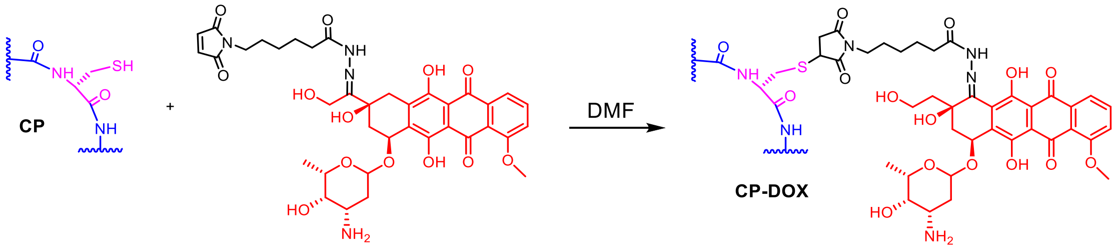

Figure S40. Procedure to obtain **CP-DOX** conjugates.

General protocol:

The corresponding CP (1 equiv.) and **1** (1 equiv.) were dissolved in 1 mL of DMF (dry). The resulting solution was stirred for 12 hours at room temperature and protected from light using aluminum foil. Then, the reaction mixture was poured over cold Et_2O (40 mL) and the red solid formed was collected by centrifugation (4000 rpm, 10 min). The product was purified by Sephadex LH-20 column (Eluent: MeOH). The pure fractions with the product were concentrated without reaching dryness, poured over cold Et_2O (40 mL), centrifuged (4000 rpm, 10 min) and dried under vacuum. The product was obtained as a red solid in all cases.[1,5]

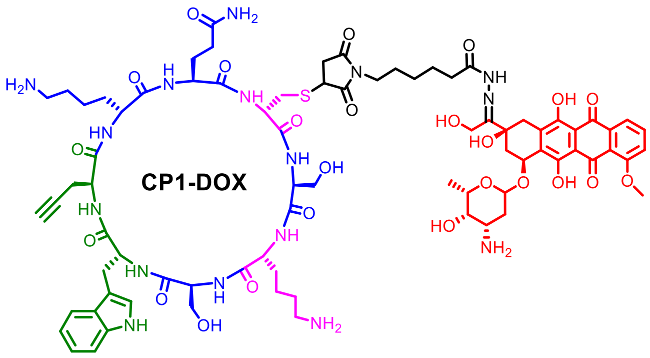

CP1-DOX: The thiol-maleimide Michael addition click reaction between **CP1** (6.0 mg, 6.4 µmol) and **8** (4.8 mg, 6.4 µmol) was performed according to the general protocol. **CP1-DOX** was obtained as a red solid with an overall yield of 70% (7.6 mg). **ESI-MS** *m/z* (%): 847.4 (100) $[M+2H]^{2+}$. **HRMS (ESI)** *m/z*: $[M+2H]^{2+}$ calculated for $C_{79}H_{106}N_{16}O_{24}S$: 847.3638, found 847.3637. **FTIR (neat)**: ν = 3270.3 (amide A), 1670.8 (amide I_b), 1629.4 (amide I_a), 1540.6 (amide II) cm^{-1}.

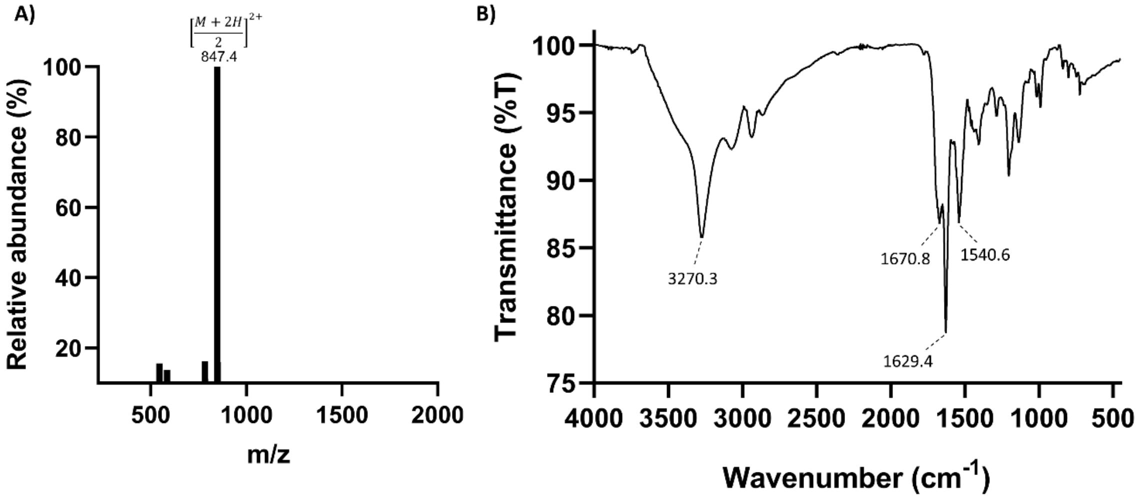

Figure S41. (A) ESI-MS of **CP1-DOX**. (B) FT-IR spectrum of **CP1-DOX** (solid, 298 K).

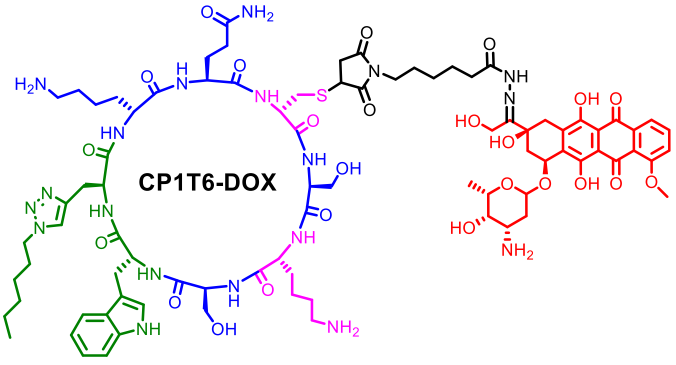

CP1T6-DOX: The thiol-maleimide Michael addition click reaction between **CP1T6** (2.1 mg, 2.0 µmol) and **1** (1.5 mg, 2.0 µmol) was performed according to the general protocol. **CP1T6-DOX** was obtained as a red solid with an overall yield of 94% (3.4 mg). **ESI-MS** *m/z* (%): 911.2 (100) $[M+2H]^{2+}$, 608.0 (25) $[M+3H]^{3+}$. **HRMS (ESI)** *m/z*: $[M+H]^{+}$ calculated for $C_{85}H_{118}N_{19}O_{24}S$: 1820.8312, found 1820.8312. **FTIR (neat)**: ν = 3270.3 (amide A), 1670.8 (amide I_b), 1625.4 (amide I_a), 1536.7 (amide II) cm^{-1}.

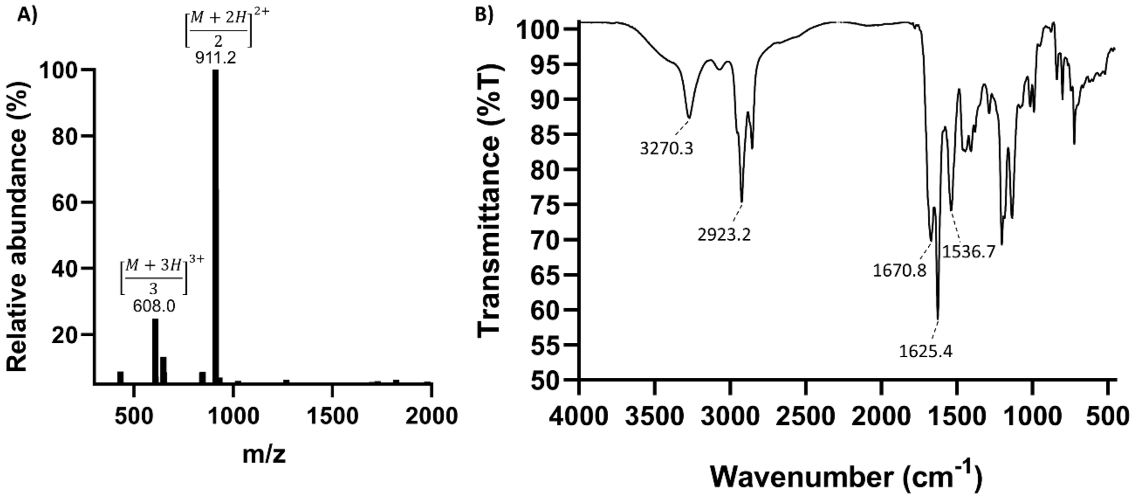

Figure S42. (A) ESI-MS of **CP1T6-DOX**. (B) FT-IR spectrum of **CP1T6-DOX** (solid, 298 K).

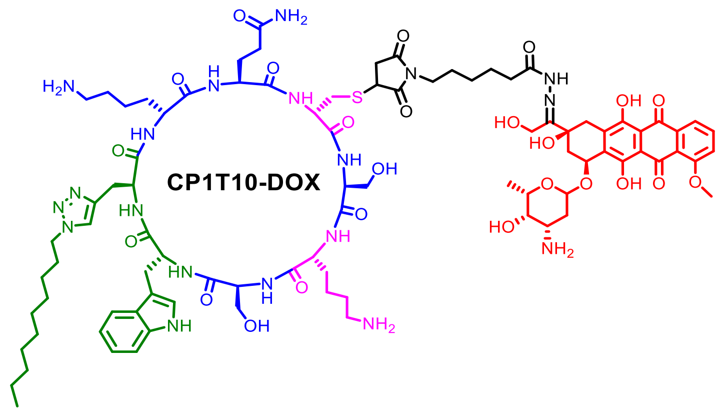

CP1T10-DOX: The thiol-maleimide Michael addition click reaction between **CP1T10** (3.0 mg, 2.7 µmol) and **1** (2.0 mg, 2.7 µmol) was performed according to the general protocol. **CP1T10-DOX** was obtained as a red solid with an overall yield of 94% (4.7 mg). **ESI-MS** *m/z* (%): 939.4 (100) $[M+2H]^{2+}$, 626.5 (36) $[M+3H]^{3+}$. **HRMS (ESI)** *m/z*: $[M+2H]^{2+}$ calculated for $C_{89}H_{127}N_{19}O_{24}S$: 938.9506, found 938.9500. **FTIR (neat)**: ν = 3274.2 (amide A), 1672.8 (amide I_b), 1627.4 (amide I_a), 1540.6 (amide II) cm^{-1}.

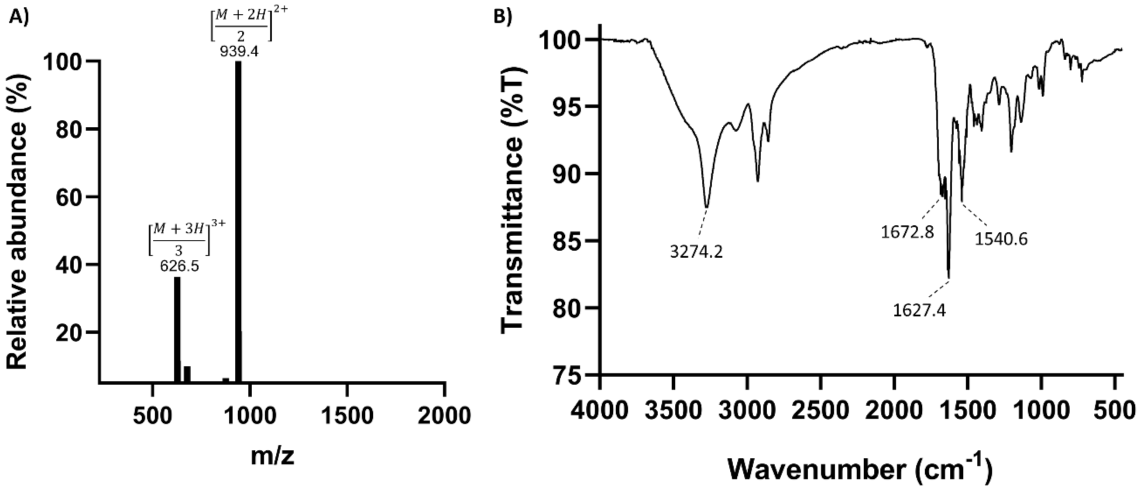

Figure S43. (A) ESI-MS of **CP1T10-DOX**. (B) FT-IR spectrum of **CP1T10-DOX** (solid, 298 K).

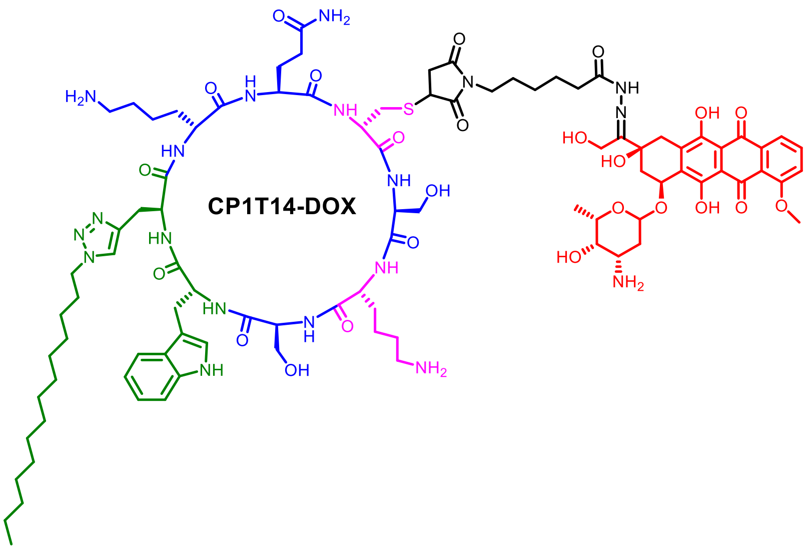

CP1T14-DOX: The thiol-maleimide Michael addition click reaction between **CP1T14** (3.5 mg, 3.0 µmol) and **1** (2.2 mg, 3.0 µmol) was performed according to the general protocol. **CP1T14-DOX** was obtained as a red solid with an overall yield of 81% (4.6 mg). **ESI-MS** *m/z* (%): 967.4 (100) $[M+2H]^{2+}$, 645.0 (26) $[M+3H]^{3+}$. **HRMS (ESI)** *m/z*: $[M+2H]^{2+}$ calculated for $C_{93}H_{135}N_{19}O_{24}S$: 966.9819, found 966.9822. **FTIR (neat)**: ν = 3274.2 (amide A), 1670.8 (amide I_b), 1629.4 (amide I_a), 1536.7 (amide II) cm^{-1}.

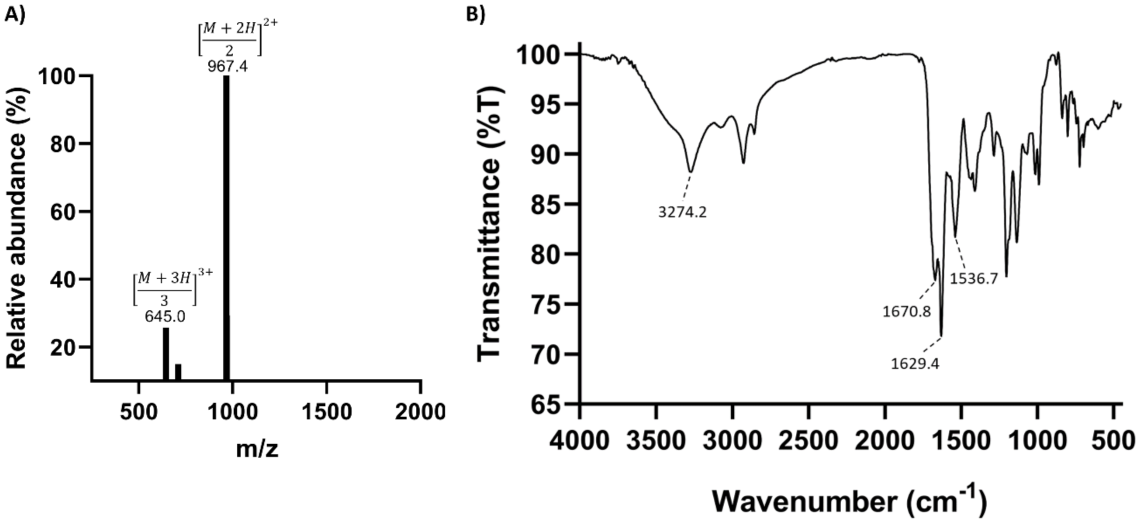

Figure S44. (A) ESI-MS of **CP1T14-DOX**. (B) FT-IR spectrum of **CP1T14-DOX** (solid, 298 K).

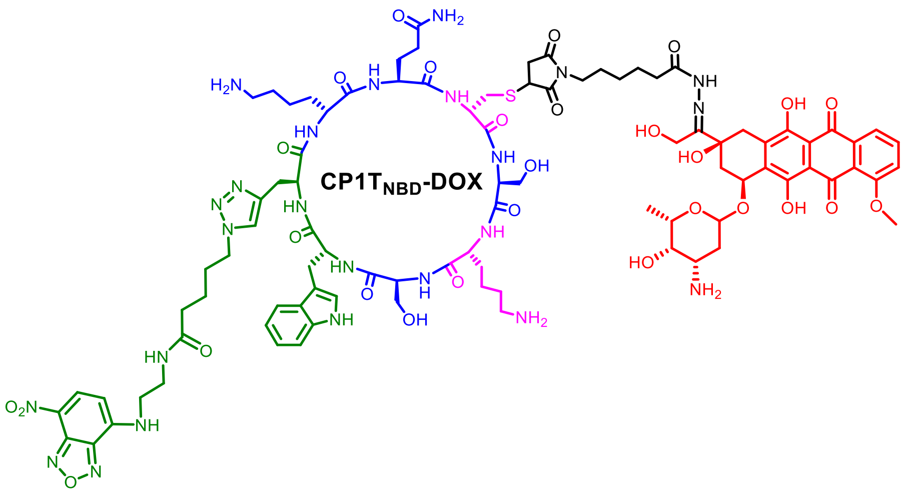

CP1T$_{NBD}$-DOX: The thiol-maleimide Michael addition click reaction between **CP1T$_{NBD}$** (3.3 mg, 2.6 µmol) and **1** (1.9 mg, 2.6 µmol) was performed according to the general protocol. **CP1T$_{NBD}$-DOX** was obtained as a red solid with an overall yield of 98% (5.1 mg). **ESI-MS** *m/z* (%): 1021.5 (100) $[M+2H]^{2+}$, 681.8 (68) $[M+3H]^{3+}$. **HRMS (ESI)** *m/z*: $[M+2H]^{2+}$ calculated for $C_{92}H_{122}N_{24}O_{28}S$: 1021.4285, found 1021.4303. **FTIR (neat)**: ν = 3274.2 (amide A), 1672.8 (amide I$_b$), 1627.4 (amide I$_a$), 1536.7 (amide II) cm^{-1}.

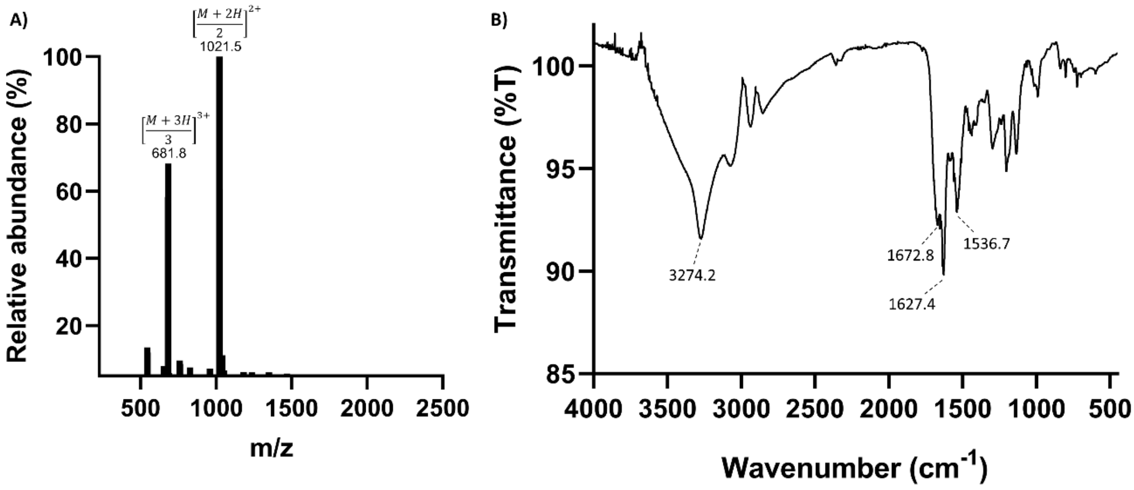

Figure S45. (A) ESI-MS of **CP1T$_{NBD}$-DOX**. (B) FT-IR spectrum of **CP1T$_{NBD}$-DOX** (solid, 298 K).

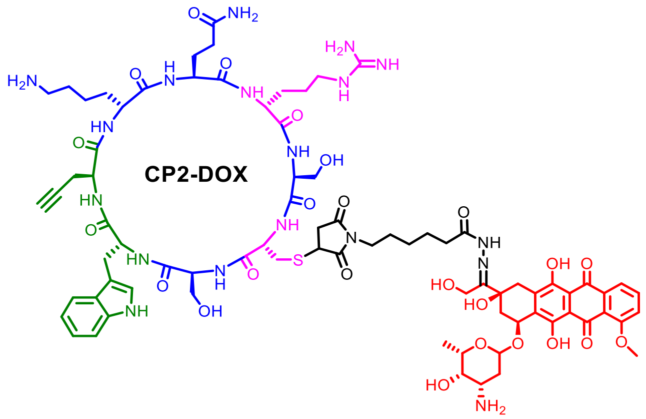

CP2-DOX: The thiol-maleimide Michael addition click reaction between **CP2** (2.6 mg, 2.7 µmol) and **1** (2.0 mg, 2.7 µmol) was performed according to the general protocol. **CP2-DOX** was obtained as a red solid with an overall yield of 78% (3.6 mg). **ESI-MS** *m/z* (%): 861.5 (100) $[M+2H]^{2+}$. **HRMS (ESI)** *m/z*: $[M+2H]^{2+}$ calculated for $C_{79}H_{106}N_{18}O_{24}S$: 861.3669, found 861.3666. **FTIR (neat)**: ν = 3276.2 (amide A), 1670.8 (amide I_b), 1627.4 (amide I_a), 1538.7 (amide II) cm^{-1}.

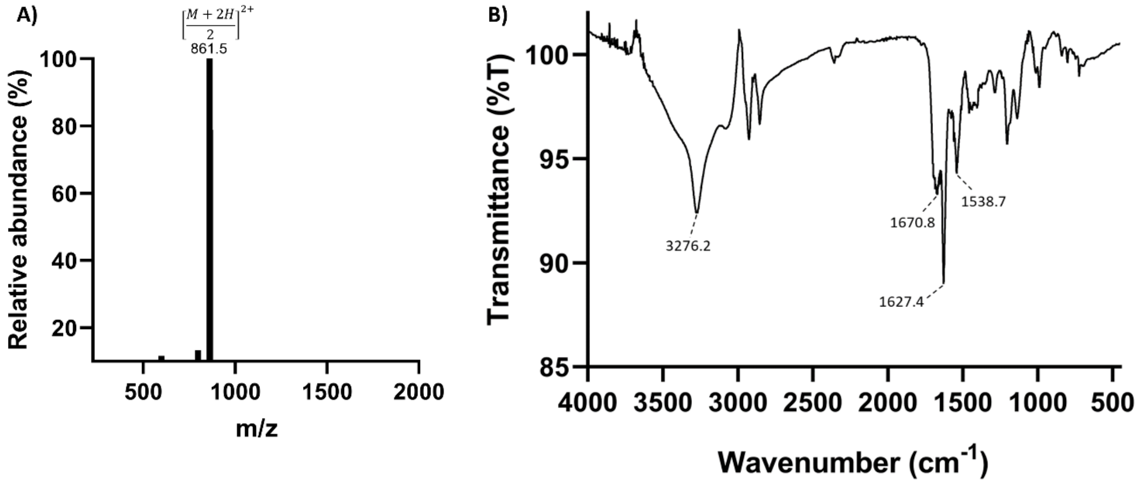

Figure S46. (A) ESI-MS of **CP2-DOX**. (B) FT-IR spectrum of **CP2-DOX** (solid, 298 K).

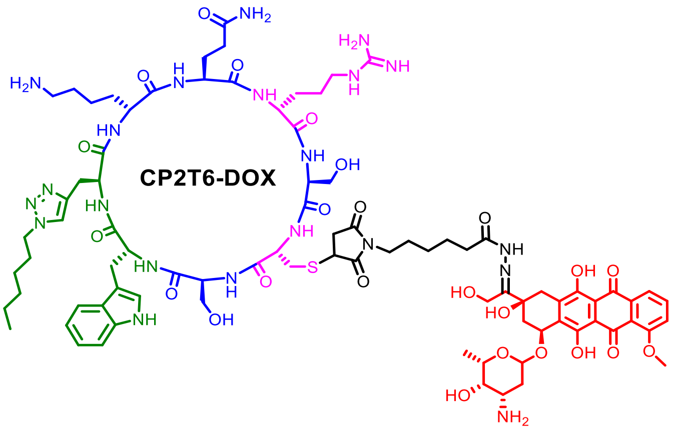

CP2T6-DOX: The thiol-maleimide Michael addition click reaction between **CP2T6** (2.5 mg, 2.3 µmol) and **1** (1.7 mg, 2.3 µmol) was performed according to the general protocol. **CP2T6-DOX** was obtained as a red solid with an overall yield of 86% (3.6 mg). **ESI-MS** *m/z* (%): 1848.6 (4) $[M+H]^+$, 925.3 (100) $[M+2H]^{2+}$, 617.1 (35) $[M+3H]^{3+}$. **HRMS (ESI)** *m/z*: $[M+2H]^{2+}$ calculated for $C_{85}H_{119}N_{21}O_{24}S$: 924.9223, found 924.9225. **FTIR (neat)**: ν = 3272.3 (amide A), 1668.8 (amide I_b), 1627.4 (amide I_a), 1538.7 (amide II) cm^{-1}.

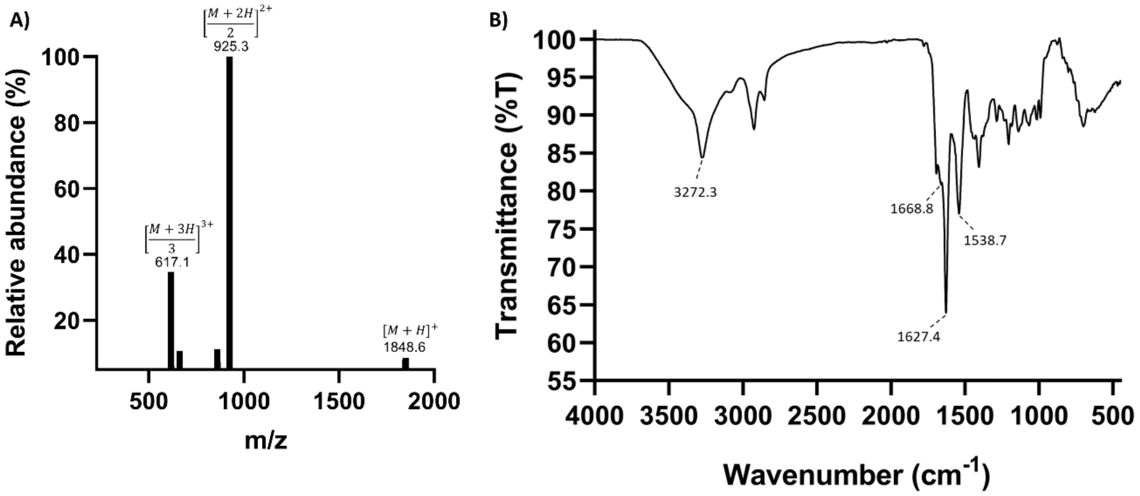

Figure S47. (A) ESI-MS of **CP2T6-DOX**. (B) FT-IR spectrum of **CP2T6-DOX** (solid, 298 K).

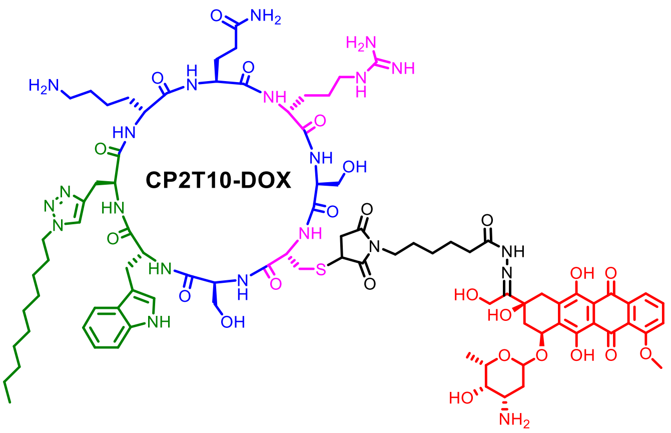

CP2T10-DOX: The thiol-maleimide Michael addition click reaction between **CP2T10** (3.0 mg, 2.6 µmol) and **1** (2.0 mg, 2.6 µmol) was performed according to the general protocol. **CP2T10-DOX** was obtained as a red solid with an overall yield of 92% (4.6 mg). **ESI-MS** *m/z* (%): 953.0 (100) $[M+2H]^{2+}$, 636.0 (50) $[M+3H]^{3+}$. **HRMS (ESI)** *m/z*: $[M+2H]^{2+}$ calculated for $C_{89}H_{127}N_{21}O_{24}S$: 952.9536, found 952.9538. **FTIR (neat)**: ν = 3272.3 (amide A), 1670.8 (amide I_b), 1625.4 (amide I_a), 1538.7 (amide II) cm^{-1}.

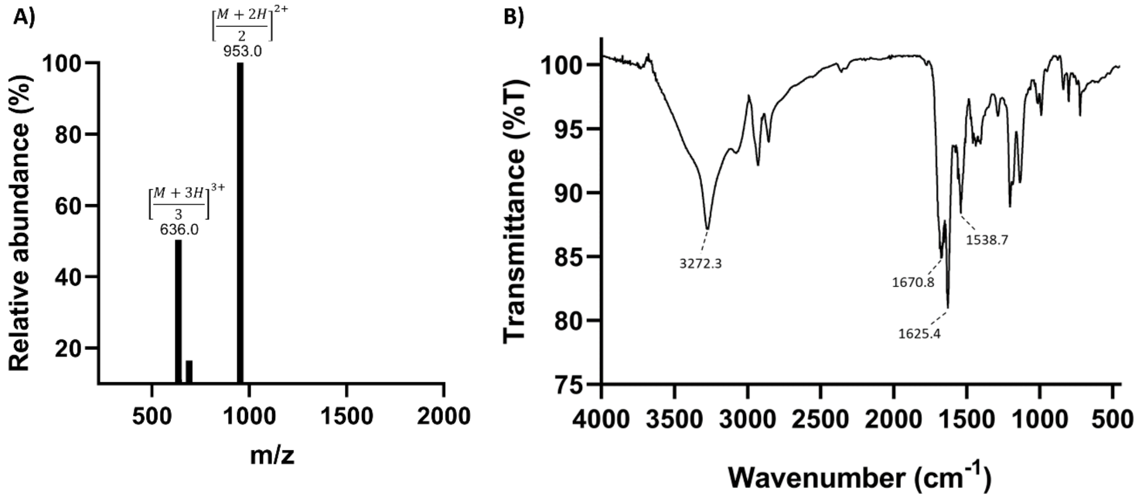

Figure S48. (A) ESI-MS of **CP2T10-DOX**. (B) FT-IR spectrum of **CP2T10-DOX** (solid, 298 K).

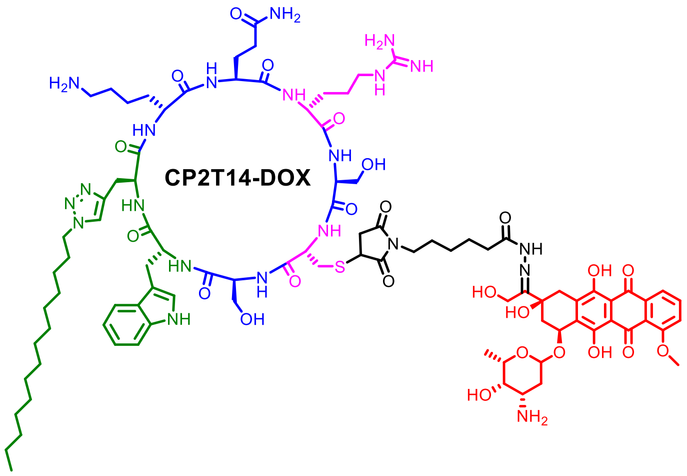

CP2T14-DOX: The thiol-maleimide Michael addition click reaction between **CP2T14** (1.5 mg, 1.2 µmol) and **1** (0.9 mg, 1.2 µmol) was performed according to the general protocol. **CP2T14-DOX** was obtained as a red solid with an overall yield of 58% (1.4 mg). **ESI-MS** *m/z* (%): 980.8 (100) $[M+2H]^{2+}$. **HRMS (ESI)** *m/z*: $[M+2H]^{2+}$ calculated for $C_{93}H_{135}N_{21}O_{24}S$: 980.9849, found 980.9850. **FTIR (neat):** ν = 3270.3 (amide A), 1668.8 (amide I_b), 1629.4 (amide I_a), 1534.7 (amide II) cm^{-1}.

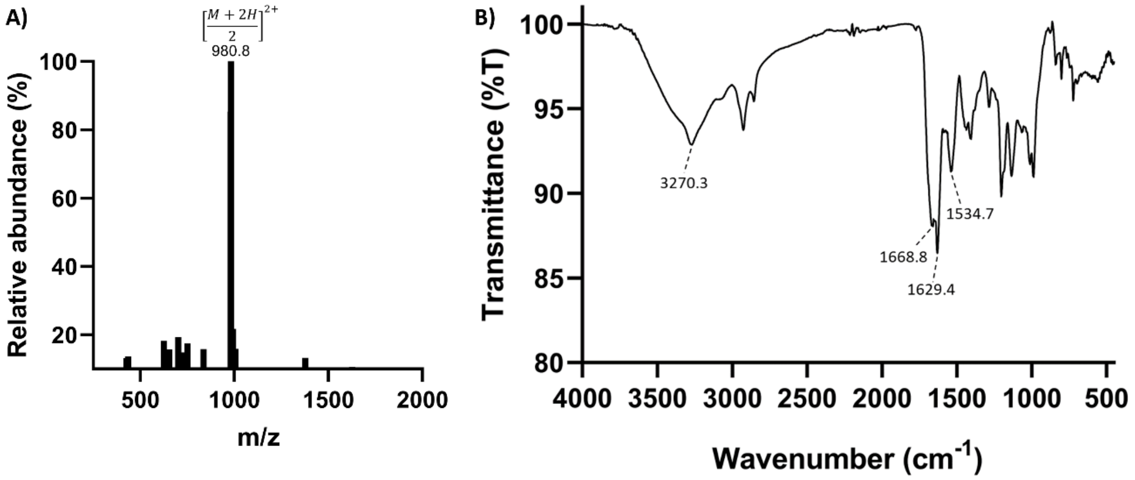

Figure S49. (A) ESI-MS of **CP2T14-DOX**. (B) FT-IR spectrum of **CP2T14-DOX** (solid, 298 K).

3. References

[1] a) D. Willner, P. A. Trail, S. J. Hofstead, H. D. King, S. J. Lasch, G. R. Braslawsky, R. S. Greenfield, T. Kaneko, R. A. Firestone, *Bioconjugate Chem.* **1993**, *4*, 521-527; b) Z. Jia, L. Wong, T. P. Davis, V. Bulmus, *Biomacromolecules* **2008**, *9*, 3106-3113; c) T. Cai, Y. Chen, Y. Wang, H. Wang, X. Liu, Q. Jin, S. Agarwal, J. Ji, *Polym. Chem.* **2014**, *5*, 4061-4068.

[2] D. Xiao, L. Luo, J. Li, Z. Wang, L. Liu, F. Xie, J. Feng, X. Zhou, *Bioorg. Chem.* **2021**, *116*, 105366.

[3] a) S. Otake, K. Okuro, D. Bochicchio, G. M. Pavan, T. Aida, *Bioconjug. Chem.* **2018**, *29*, 2068-2073; b) S. Taliani, F. Simorini, V. Sergianni, C. La Motta, F. Da Settimo, B. Cosimelli, E. Abignente, G. Greco, E. Novellino, L. Rossi, V. Gremigni, F. Spinetti, B. Chelli, C. Martini, *J. Med. Chem.* **2007,** *50*, 404-407; c) X. Xu, Z. Kuang, J. Han, Y. Meng, L. Li, H. Luan, P. Xu, J. Wang, C. Luo, H. Ding, Z. Li, J. Bian, *J. Med. Chem.* **2019**, *62*, 9642-9657; d) M. Ciba, B. Dibnah, B. D. Hudson, E. R. Ulven, *J. Med. Chem.* **2023**, *66*, 8951-8974.

[4] a) E. González-Freire, F. Novelli, A. Pérez-Estévez, R. Seoane, M. Amorín, J. R. Granja, *Chem. Eur. J.* **2021**, *27*, 3029-3038; b) J. M. Palomo, *RSC Adv.* **2014**, *4*, 32658-32672; c) P. Rovero, L. Quartara, G. Fabbri, *Tetrahedron Lett.* **1991**, *32*, 2639-2642; d) A. El-Faham, F. Albericio, *Chem. Rev.* **2011**, *111*, 6557-6602; e) F. Albericio, *Biopolymers* **2000**, *55*, 123-139.

[5] a) B. H. Northrop, S. H. Frayne, U. Choudhary, *Polym. Chem.* **2015**, *6*, 3415-3430; b) S. Li, L. Liu, H. Jia, W. Qiu, L. Rong, H. Cheng, X. Zhang, *Chem. Commun.* **2014**, *50*, 11852-11855; c) J. Wang, M. Chen, S. Li, R. D. Ye, *Mol. Pharmaceutics* **2019**, *16*, 2636-2647.
